# Evaluation of Population-Level Tobacco Control Interventions and Health Outcomes: A Systematic Review and Meta-Analysis

**DOI:** 10.1001/jamanetworkopen.2023.22341

**Published:** 2023-07-07

**Authors:** Shamima Akter, Md. Rashedul Islam, Md. Mizanur Rahman, Thomas Rouyard, Raïssa Shiyghan Nsashiyi, Fahima Hossain, Ryota Nakamura

**Affiliations:** 1 Research Center for Health Policy and Economics, Hitotsubashi Institute for Advanced Study, Hitotsubashi University, Tokyo, Japan; 2 Institute for Nature, Health, and Agricultural Research (INHAR), Yaoundé, Cameroon; 3 Global Public Health Research Foundation, Dhaka, Bangladesh; 4 Graduate School of Economics, Hitotsubashi University, Tokyo, Japan

## Abstract

**Importance:**

Smoking causes considerable noncommunicable diseases, perinatal morbidity, and mortality.

**Objective:**

To investigate the associations of population-level tobacco-control policies with health outcomes.

**Data Sources:**

PubMed, EMBASE, Web of Science, Cumulated Index to Nursing and Allied Health Literature, and EconLit were searched from inception to March 2021 (updated on 1 March 2022). References were manually searched.

**Study Selection:**

Studies reporting on associations of population-level tobacco control policies with health-related outcomes were included. Data were analyzed from May to July 2022.

**Data Extraction and Synthesis:**

Data were extracted by 1 investigator and cross-checked by a second investigator. Analyses were conducted using the Preferred Reporting Items for Systematic Reviews and Meta-analyses reporting guideline.

**Main Outcomes and Measures:**

The primary outcomes were respiratory system disease (RSD), cardiovascular disease (CVD), cancer, mortality, hospitalization, and health care utilization. The secondary outcomes were adverse birth outcomes, such as low birth weight and preterm birth. Random-effects meta-analysis was used to estimate pooled odds ratios (ORs) and 95% CIs.

**Results:**

Of 4952 records identified, 144 population-level studies were included in the final analysis; 126 studies (87.5%) were of high or moderate quality. The most frequently reported policies were smoke-free legislation (126 studies), followed by tax or price increases (14 studies), multicomponent tobacco control programs (12 studies), and a minimum cigarette purchase age law (1 study). Smoke-free legislation was associated with decreased risk of all CVD events (OR, 0.90; 95% CI, 0.86-0.94), RSD events (OR, 0.83; 95% CI, 0.72-0.96), hospitalization due to CVD or RSD (OR, 0.91; 95% CI, 0.87-0.95), and adverse birth outcomes (OR, 0.94; 95% CI, 0.92-0.96). These associations persisted in all sensitivity and subgroup analyses, except for the country income category, for which a significant reduction was only observed in high-income countries. In meta-analysis, there was no clear association of tax or price increases with adverse health outcomes. However, for the narrative synthesis, all 8 studies reported statistically significant associations between tax increases and decreases in adverse health events.

**Conclusions and Relevance:**

In this systematic review and meta-analysis, smoke-free legislation was associated with significant reductions in morbidity and mortality related to CVD, RSD, and perinatal outcomes. These findings support the need to accelerate the implementation of smoke-free laws to protect populations against smoking-related harm.

## Key Points

**Question:**

Are population-level tobacco control interventions associated with lower tobacco-linked adverse health outcomes?

**Findings:**

In this systematic review and meta-analysis including 144 population-level studies, smoke-free legislation was associated with beneficial cardiovascular and respiratory health outcomes as well as birth outcomes. Health outcomes were not clearly associated with tax or price increases or other policies; however, the lack of relevant studies could explain this.

**Meaning:**

These studies support the use of smoke-free legislation to improve population-level cardiovascular, respiratory, and birth outcomes.

## Teaser

This systematic review and meta-analysis assessed associations of population-level tobacco control policy with cardiovascular, respiratory and perinatal health outcomes.

## Introduction

The global health burden associated with tobacco use is high. Despite the implementation of numerous tobacco control policies to change smoking behaviors over the past decades,^1^ tobacco use remains the second leading risk factor of mortality, with 8.7 million attributable deaths worldwide in 2019.^2^ Exposure to tobacco smoke, including secondhand smoke (SHS), has been linked to adverse health outcomes in children and adults, particularly chronic noncommunicable diseases, such as cardiac, cerebrovascular, and respiratory diseases and cancers.^3,4,5,6,7,8,9^ Over 2 decades, to reduce smoking-related morbidity and mortality, various evidence-based tobacco control policies have been proposed, such as the Framework Convention on Tobacco Control by the World Health Organization.^10^ Framework Convention on Tobacco Control policies, including taxations, regulations, and nicotine replacement therapies, have been found to be associated with lowering the incidence of adverse health outcomes in multiple settings.^11,12^

To our knowledge, 12 systematic reviews and meta-analyses have synthesized evidence of the associations of population-level tobacco control interventions with health outcomes (eTable 1 in Supplement 1).^13,14,15,16,17,18,19,20,21,22,23,24,25^ The evidence suggests that regulatory policies, particularly those prohibiting smoking, such as local or public smoking bans, were associated with reduced risks of cardiovascular, cerebrovascular, and respiratory diseases^16,24^ and improved perinatal outcomes.^13,15^ However, these syntheses focused on the associations of single or select interventions separately with specific outcomes or populations (children^13,15,25^ or adults^14,16,17,18,19,20,21,22,23,24^). In a 2012 meta-analysis, Tan and Glantz^24^ assessed the pooled risk estimation for the association between smoke-free legislation and hospitalization due to cardiovascular disease (CVD) or respiratory system diseases (RSD) but did not focus on the incidence or prevalence of these conditions. Moreover, associations with other types of population-level policies, such as advertisement campaigns, health warnings on product packages, and taxation of tobacco products, have not been fully explored. While tobacco taxation has been found to be associated with reductions in neonatal and infant mortality,^26^ there is no systematic review and meta-analysis of its associations with other health outcomes, to our knowledge. Despite these gaps, many countries have continued implementing the tobacco control policies in their endeavors to improve population health and reduce health care costs.^16^ Systematic reviews and meta-analyses are crucial to quantify the extent to which each tobacco control policy is associated with improving health outcomes and would inform the design and roll-out of more cost-effective policies.

This study provides a comprehensive synthesis of the associations between population-level tobacco control policies and a range of health-related outcomes. Through systematic review and meta-analysis, we aimed to summarize the associations of implementation of all available population-level tobacco control policies with health-related outcomes and estimate a pooled effect size for each relevant combination of policies with outcomes of interest.

## Methods

### Search Strategy

This study followed the Preferred Reporting Items for Systematic Reviews and Meta-analyses (PRISMA) reporting guideline. The protocol was registered with PROSPERO (registration No. CRD42022340141). A global search was conducted for studies that report on associations of population-level tobacco control policies with health-related outcomes. We searched for peer-reviewed journal articles and gray literature published from inception to March 2021 (updated on March 1, 2022) using 5 electronic databases: PubMed, Embase, Cumulated Index to Nursing and Allied Health Literature, Web of Science, and EconLit. Complementary searches included reference lists of primary studies and systematic reviews, Google Scholar, and leading organizational websites. Letters, case series, systematic or scoping reviews, commentaries, and editorials without original research findings were excluded. Details of the search strategy and results are presented in eTables 2 through 6 in Supplement 1.

### Eligibility Criteria

We summarize eligibility criteria using the PICOS (population, intervention, comparator, outcome, study type) framework. No restriction was applied on population type, condition, or age. Included interventions were population-level policies aimed at reducing tobacco use, ie, interventions or programs implemented outside controlled settings. All policies implemented by governments and nongovernmental organizations were included, including smoking bans, taxation, and minimum cigarette purchase age. Outcomes of interest were respiratory symptoms and related diseases (asthma, chronic obstructive pulmonary disease, pneumonia, bronchitis, spontaneous pneumothorax, and lung cancer), cardiovascular symptoms and related diseases (acute myocardial infarction, ischemic heart disease, acute coronary syndrome, sudden circulatory arrest, angina, congestive heart failure, hypertension), cerebrovascular diseases (stroke, transient ischemic attack), sudden cardiac death, cancers, as well as overall mortality and mortality associated with these conditions. Also, hospitalization, health care utilization, and attendance at health check-ups due to CVD, RSD, or related symptoms were assessed. Finally, we considered adverse perinatal outcomes, such as low birth weight, preterm birth, small for gestational age, still birth, infant mortality, and neonatal mortality. No restriction was applied to the type of comparator.

### Study Design

Included studies were observational studies of any design, such as cross-sectional, case-control, cohort studies, and quasi-experimental studies (eg, interrupted time series, before and after, and controlled before and after). Studies that used a controlled environment to estimate intervention effects (eg, randomized clinical trials) and simulation or modeling studies were excluded.

### Study Selection

Two assessors independently screened the titles and abstracts (first stage) and critically reviewed the full texts of the selected studies (second stage) to assess eligibility. Disagreements at either stage were resolved through discussion with S.A. and R.N. Study authors were contacted if relevant information on eligibility appeared to be missing at the full-text screening stage.

### Data Extraction

A preconceived and standardized data extraction form was used to collect information on the first author’s last name, study country, publication year, survey year, study design, sample size, age range, type of intervention or policy, outcome variable, and quantitative estimates of the associations between policy interventions and the outcomes of interest (eg, percentage, prevalence, odds ratio [OR], risk ratio, hazard ratio [HR]) (eAppendix 1 in Supplement 1). One investigator independently extracted data from the selected primary studies. The extracted data were then cross-checked by a second investigator (S.A. or R.S.N.). Disagreements were resolved consensually.

Estimates for each disease or health measure in studies that reported multiple outcomes (eg, CVDs, cancers, and RSDs) were identified and separately listed. Regression coefficients were reported using logistic regression, and ORs were calculated using exponentiation and *P* values. Where regression coefficients were reported using linear regression, we calculated the pooled regression coefficient using each coefficient value and the SE or *P* value. A study that provided multiple ORs, relative risks (RRs), or HRs due to the use of categorical exposure data, such as by sex, geographic area, or age group, were combined into a single overall estimate by running a meta-analysis. Whenever a study provided multiple effect size estimates from statistical models adjusted for different covariates, we selected the estimate that had been adjusted for the greatest number of variables. Finally, when a study provided a percentage change or mean difference with or without *P* values, we listed those values and summarized the evidence.

### Study Quality Assessment

The Newcastle-Ottawa Scale was used to assess the quality of observational studies.^27^ The quality of controlled before and after, interrupted time series, and other quasi-experimental studies were coded using the Cochrane Effective Practice and Organisation of Care Tools^28^ (eAppendix 2 in Supplement 2).

### Statistical Analysis

A meta-analysis was performed for studies that presented complete data with statistical testing. A narrative synthesis was performed for studies without statistical testing or with high heterogeneity across measures. For meta-analysis, to summarize effect sizes, we performed fixed- or random-effects meta-analyses depending on the degree of heterogeneity. For dichotomous outcomes, ORs, RRs, or HRs with 95% CIs were used to calculate pooled estimates. Regression coefficients with SDs or 95% CIs were used for continuous outcome variables. In line with a previous study,^29^ we treated ORs as equal to RRs (the most commonly reported type of estimate) each time the incidence of the outcome of interest was low (<10%) in the study population. Funnel plots and Egger test^30^ were used to assess publication bias. We performed trim and fill procedures to further evaluate possible effects of publication bias.^31^ We conducted subgroup analyses according to study designs, country-income categories, study quality, and comprehensiveness of smoke-free legislations. Sensitivity analyses were performed by excluding highly influential studies with large sample sizes. Data analyses were performed using Stata software version 16.1 MP (StataCorp) and R software version 3.6.4 (R Project for Statistical Computing). *P* values were 2-sided, and statistical significance was set at *P* < .05. Data were analyzed from May to July 2022.

## Results

### Study Selection

A total of 4952 studies were identified from the initial search, of which 192 studies were added after a manual search and the gray literature. After removal of duplicates, 4408 unique citations were screened for title and abstract, and 515 full-text articles were screened for eligibility. Of these, 144 population-level studies^32,33,34,35,36,37,38,39,40,41,42,43,44,45,46,47,48,49,50,51,52,53,54,55,56,57,58,59,60,61,62,63,64,65,66,67,68,69,70,71,72,73,74,75,76,77,78,79,80,81,82,83,84,85,86,87,88,89,90,91,92,93,94,95,96,97,98,99,100,101,102,103,104,105,106,107,108,109,110,111,112,113,114,115,116,117,118,119,120,121,122,123,124,125,126,127,128,129,130,131,132,133,134,135,136,137,138,139,140,141,142,143,144,145,146,147,148,149,150,151,152,153,154,155,156,157,158,159,160,161,162,163,164,165,166,167,168,169,170,171,172,173,174,175,176^ met the eligibility criteria and were included in analysis (eFigure 1 in Supplement 1).

### Study Characteristics

eTable 7 in Supplement 1 shows characteristics of the included studies. Of 144 studies, 26 studies^39,46,51,52,55,71,75,97,99,104,106,107,108,110,126,131,135,138,141,142,146,153,154,156,158,173^ were cohort or longitudinal, 66 studies^32,34,36,37,38,43,44,45,48,49,50,53,54,56,58,59,61,65,66,67,69,73,74,78,79,80,81,82,83,84,85,87,88,89,91,92,93,94,98,111,112,113,114,115,117,119,120,121,122,124,127,137,140,143,144,147,148,150,151,152,164,165,170,171,172,173,174^ were interrupted time series, 25 studies^40,42,62,63,72,76,77,90,96,102,116,123,125,130,133,134,145,149,155,157,159,162,167,168,175^ were controlled before and after, and 27 studies^33,35,41,47,52,57,60,64,69,70,86,95,100,101,105,109,118,129,132,136,139,160,161,163,166,176^ were cross-sectional. The plurality of the studies (72 studies^33,40,41,42,46,47,49,51,53,54,56,57,59,60,61,62,63,65,66,67,69,72,74,75,76,77,79,80,90,91,93,95,98,99,105,108,109,110,114,115,117,119,120,121,122,123,124,125,126,128,130,132,133,136,137,143,144,145,146,149,151,155,157,158,159,160,161,162,168,172,173,175^ [50.0%]) were conducted in the United States, 45 studies^32,35,37,38,39,48,50,52,55,58,64,68,70,71,82,83,84,85,86,92,94,96,100,101,102,103,104,106,107,111,112,113,116,118,129,142,150,152,154,156,163,166,167,170,171^ (31.2%) were conducted in Europe, 14 studies^34,43,44,45,73,78,97,138,139,140,141,148,153,164^ (9.7%) were conducted in the United Kingdom, and 13 studies^36,81,87,88,89,127,131,134,135,147,165,174,176^ (9.0%) were conducted elsewhere (mostly in Asia and Latin America). Most studies evaluated smoke-free legislation (126 studies^32,33,34,35,36,37,38,39,40,41,42,43,44,45,47,48,49,50,51,52,53,54,55,56,58,59,61,62,63,64,65,66,67,68,69,70,71,72,73,74,75,77,78,79,80,81,82,83,84,85,86,87,88,89,90,91,92,93,94,96,97,99,100,101,102,103,104,105,106,107,108,110,111,112,113,114,115,116,117,118,119,120,121,122,123,125,128,129,130,131,133,134,135,136,138,139,140,141,142,144,145,146,147,148,149,150,153,154,155,156,157,158,160,161,162,163,164,165,166,167,168,170,171,172,173,176^ [87.5%]), 14 studies^6,60,61,63,66,81,109,121,122,137,144,145,151,159^ (9.7%) evaluated tax or price increases, 12 studies^53,57,76,95,98,124,126,127,132,143,152,174^ (8.3%) assessed multicomponent tobacco control programs, and 1 study^175^ (0.7%) focused only on minimum cigarette purchase age. The included studies were published between 1998 and 2021. In terms of quality, 86 studies^33,39,40,41,43,44,47,49,51,55,57,58,59,60,61,62,63,64,65,66,67,68,69,70,71,72,73,74,75,77,80,81,82,83,85,86,89,90,91,96,99,100,103,105,109,111,112,115,117,118,119,120,121,122,127,128,129,130,132,134,136,137,139,140,141,143,145,146,147,150,151,152,153,155,156,159,160,161,162,163,164,166,167,171,174,176^ were rated high, 40 studies^32,34,35,36,37,38,45,46,48,50,76,78,79,87,88,92,93,94,95,97,98,102,104,108,110,114,123,124,125,131,133,135,138,142,144,148,149,157,158,173^ were rated moderate, and 18 studies^42,52,53,54,56,84,101,106,107,113,116,126,154,165,168,170,172,175^ were rated poor. Of 144 studies, 60 studies^32,33,34,35,36,37,38,39,40,41,42,43,44,45,46,47,48,49,50,51,52,53,54,55,56,57,58,59,60,61,62,63,64,65,66,67,68,69,70,71,72,73,74,75,76,77,78,79,80,81,82,83,84,85,86,87,88,89,90,91,92^ were included in the meta-analysis, 84 studies^33,93,94,95,96,97,98,99,100,101,102,103,104,105,106,107,108,109,110,111,112,113,114,115,116,117,118,119,120,121,122,123,124,125,126,127,128,129,130,131,132,133,134,135,136,137,138,139,140,141,142,143,144,145,146,147,148,149,150,151,152,153,154,155,156,157,158,159,160,161,162,163,164,165,166,167,168,169,170,171,172,173,174,175,176^ were included in the narrative summary, and 1 study^33^ was included in both the meta-analysis and narrative summary.

### Meta-Analysis

Only 60 studies^32,33,34,35,36,37,38,39,40,41,42,43,44,45,46,47,48,49,50,51,52,53,54,55,56,57,58,59,60,61,62,63,64,65,66,67,68,69,70,71,72,73,74,75,76,77,78,79,80,81,82,83,84,85,86,87,88,89,90,91,92^ reported quantitative information (ie, OR, RR, HR, or coefficient) suitable for calculating pooled estimates. Effect sizes reported in the other 84 studies^33,93,94,95,96,97,98,99,100,101,102,103,104,105,106,107,108,109,110,111,112,113,114,115,116,117,118,119,120,121,122,123,124,125,126,127,128,129,130,131,132,133,134,135,136,137,138,139,140,141,142,143,144,145,146,147,148,149,150,151,152,153,154,155,156,157,158,159,160,161,162,163,164,165,166,167,168,169,170,171,172,173,174,175,176^ were summarized narratively. A standard meta-analysis (ie, with >2 studies) was possible for studies assessing smoke-free legislation. Studies on other types of policies were too heterogeneous in terms of outcomes measured or types of data used. Findings are presented in the form of forest plots in eFigures 2 through 6 in Supplement 1 to provide an overview.

**Figure 1.**
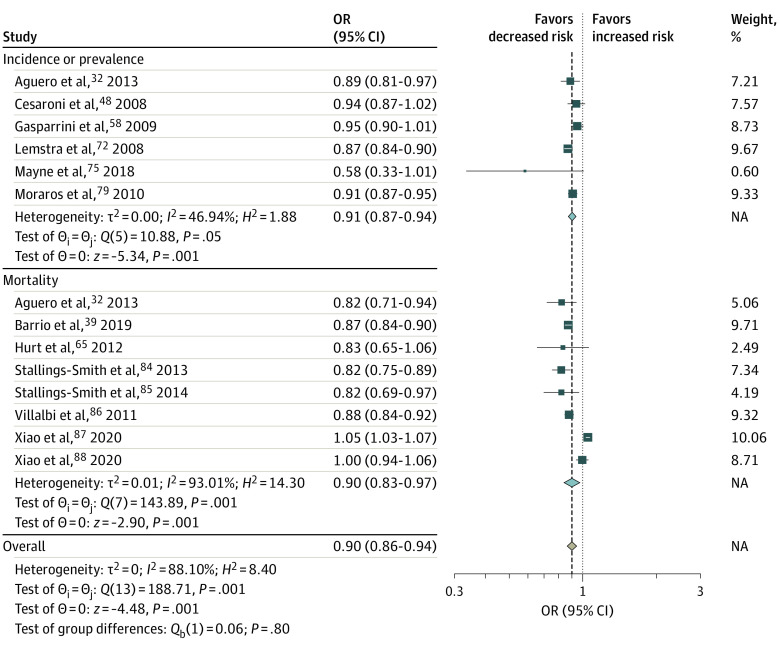
Meta-Analysis of the Associations of Smoke-Free Legislation With Cardiovascular Events Cardiovascular events included the incidence, prevalence, and mortality due to acute myocardial infarction, heart attack, sudden cardiac death, coronary heart disease, and cerebrovascular disease. Squares indicate estimates; size of squares, study weights; whiskers, 95% CIs; diamond, mean estimate; NA, not applicable; OR, odds ratio.

Of 60 studies^32,33,34,35,36,37,38,39,40,41,42,43,44,45,46,47,48,49,50,51,52,53,54,55,56,57,58,59,60,61,62,63,64,65,66,67,68,69,70,71,72,73,74,75,76,77,78,79,80,81,82,83,84,85,86,87,88,89,90,91,92^ included in the meta-analysis, 15 studies^32,39,48,50,58,65,72,75,79,84,85,86,87,88,92^ assessed the association of smoke-free legislation with incidence or prevalence of CVD or CVD mortality (Figure 1; eFigure 2 in Supplement 1). We found that smoke-free legislation was associated with significant reductions in the incidence or prevalence of CVD (OR, 0.91; 95% CI, 0.87-0.94), CVD mortality (OR, 0.90; 95% CI, 0.83-0.97), and occurrence of any type of CVD event (OR, 0.90; 95% CI, 0.86-0.94) (Figure 1).

**Figure 2.**
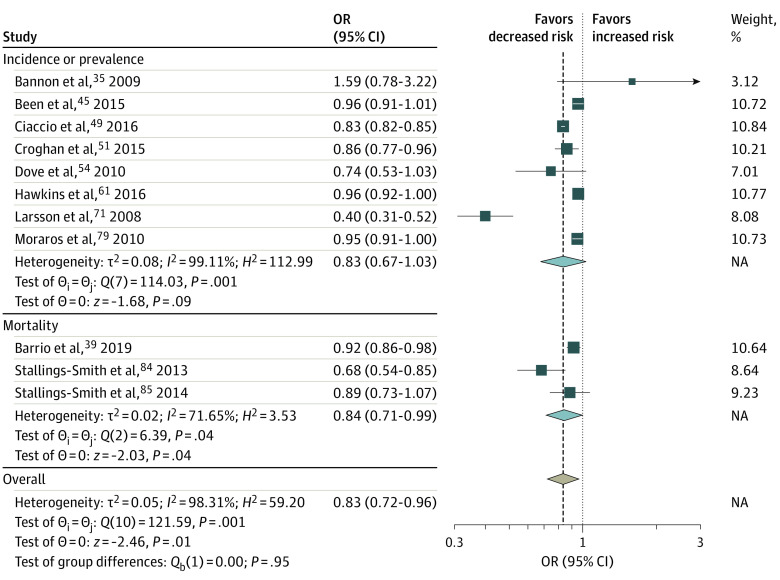
Meta-Analysis of the Associations of Smoke-Free Legislation With Respiratory Disease or Respiratory Symptoms Events Respiratory disease or respiratory symptoms included the prevalence and mortality of lung cancer, respiratory symptoms, chronic obstructive pulmonary disease, asthma, and bronchitis. Squares indicate estimates; size of squares, study weights; whiskers, 95% CIs; diamond, mean estimate; NA, not applicable; OR, odds ratio.

**Figure 3.**
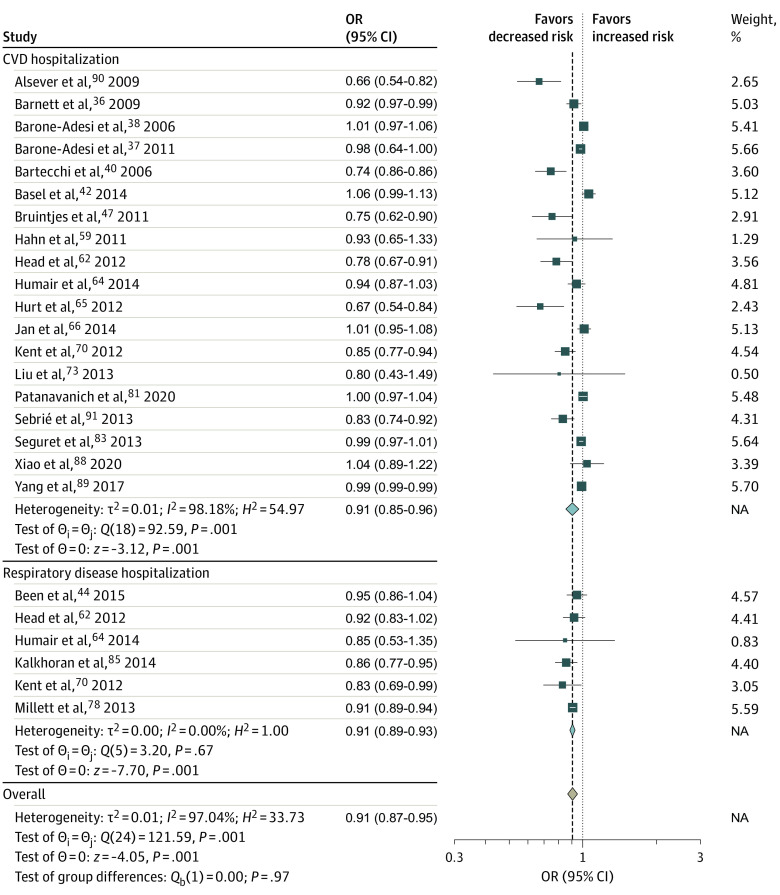
Meta-Analysis of the Associations of Smoke-Free Legislation With Hospital Admission Rate Due to Cardiovascular Disease and Respiratory Disease and Symptoms Cardiovascular disease hospital admission rates included admissions due to cardiovascular disease, ischemic heart disease, angina, acute coronary syndrome, coronary heart disease, acute myocardial infarction, heart attack, cerebrovascular disease, and stroke. Hospitalization due to respiratory disease or respiratory symptoms included admission due to lung cancer, respiratory symptoms, chronic obstructive pulmonary disease, asthma, and bronchitis. Squares indicate estimates; size of squares, study weights; whiskers, 95% CIs; diamond, mean estimate; NA, not applicable; OR, odds ratio.

Twelve studies^35,39,45,49,51,55,61,71,79,84,85,105^ assessed the association of smoke-free legislation with the prevalence or incidence of RSD, RSD symptoms, or RSD mortality (Figure 2; eFigure 2 in Supplement 1). Smoke-free legislation was not found to be significantly associated with prevalence of RSD or RSD symptoms (OR, 0.83; 95% CI, 0.67-1.03) (Figure 2) but was significantly associated with reductions in RSD mortality (OR, 0.91; 95% CI, 0.85-0.96) and occurrence of any RSD, RSD symptoms, or RSD mortality (OR, 0.83; 95% CI, 0.72-0.96) (Figure 2). The association of smoke-free legislation with hospitalizations induced by CVD was investigated in 24 studies,^36,37,38,42,47,52,53,59,62,63,64,65,66,67,70,73,74,81,83,87,89,90,91,99^ and the association of smoke-free legislation with hospitalizations induced by RSD or RSD symptoms was investigated in 9 studies^44,52,53,55,62,63,64,69,70,78^ (Figure 3; eFigure 2 in Supplement 1). In both analyses, the smoke-free legislation was found to be associated with significant reductions in hospitalizations (Figure 3).

**Figure 4.**
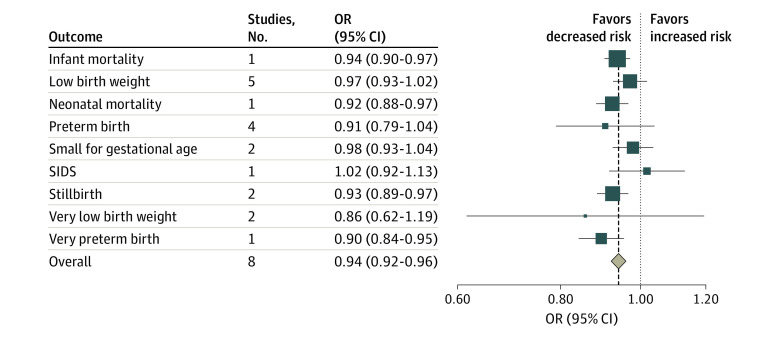
Meta-Analysis of the Associations of Smoke-Free Legislation With Perinatal Mortality and Adverse Birth Outcomes Squares indicate estimates; size of squares, study weights; whiskers, 95% CIs; diamond, mean estimate; OR, odds ratio; SIDS, sudden infant death syndrome.

Eight studies^33,34,41,43,68,77,80,82^ estimated the associations between smoke-free legislation and adverse birth outcomes, including infant mortality, neonatal mortality, stillbirth, preterm birth, very preterm birth, small for gestational age, low birth weight, very low birth weight, and sudden infant death syndrome (Figure 4; eFigure 3 in Supplement 1). Although the pooled estimates for low birth weight, preterm birth, and small for gestational age were not statistically significant, significant associations were found for stillbirth (OR, 0.93; 95% CI, 0.890.97) and overall birth (OR, 0.94; 95% CI, 0.92-0.96) outcomes (Figure 4).

Limited research has been conducted on the associations of tobacco tax policies with health-related outcomes. The available data suffer from heterogeneity in terms of measured outcomes (eg, hospitalization, incidence, or mortality for various health conditions) or data types used (eg, some studies presented data as ORs while others as regression coefficient), making a standardized meta-analysis unfeasible. For instance, 4 studies^53,63,66,81^ examined CVD-related hospitalizations, 2 studies^60,61^ focused on the prevalence or incidence of RSD and RSD symptoms, 2 studies^53,63^ explored RSD- and RSD symptom–related hospitalizations, and 1 study^46^ investigated RSD-related mortality (eFigure 4 and eFigure 5 in Supplement 1). No significant associations were found for any of these outcomes. Additionally, 2 studies^46,53^ focused on cancer incidence or mortality, and another study^46^ investigated total mortality (eFigure 4 and eFigure 5 in Supplement 1). While tax policies demonstrated a significant association with reduced cancer mortality and total mortality, no significant association was observed for cancer incidence.

Finally, 3 studies^53,57,76^ assessed the association of multicomponent tobacco control programs with CVD mortality, CVD-related hospitalizations, RSD- or RSD symptom–related hospitalizations, and cancer incidence (eFigure 6 in Supplement 1). Mixed results have been reported regarding reductions in CVD-related mortality risks.^57,76^ One study^76^ found multicomponent tobacco control programs to be significantly associated with decreased CVD mortality, while another study reported a significant increase in CVD mortality.^57^ The other study^53^ found that multicomponent tobacco control programs were significantly associated with reduced CVD-related hospitalizations but not cancer incidence or respiratory disease-related hospitalizations.

We found evidence of large heterogeneity in the association between smoke-free legislation and CVD events (*I*^2^ = 88.1%; P < .001) (Figure 1), RSD and RSD symptom events (*I*^2^ = 98.3%; P < .001) (Figure 2), hospitalization due to CVD and RSD and RSD symptom (*I*^2^ = 97.0%; P < .001) (Figure 3), and adverse birth outcomes (*I*^2^ = 76% to 97%; *P* < .01). However, results of the main meta-analysis were consistent in the sensitivity (eAppendix 3, eFigures 7-9, and eTable 8 in Supplement 1) and stratified (eAppendix 4, eTable 9, and eTable 10 in Supplement 1) analyses, except for country income category, for which a significant reduction was observed in high-income countries.

### Narrative Summary

The Table presents a narrative summary of the associations of population-level tobacco control policies with health-related outcomes. More details are provided in eTables 11 through 14 in Supplement 1. The narrative summary included 84 studies.^33,93,94,95,96,97,98,99,100,101,102,103,104,105,106,107,108,109,110,111,112,113,114,115,116,117,118,119,120,121,122,123,124,125,126,127,128,129,130,131,132,133,134,135,136,137,138,139,140,141,142,143,144,145,146,147,148,149,150,151,152,153,154,155,156,157,158,159,160,161,162,163,164,165,166,167,168,169,170,171,172,173,174,175,176^ After the implementation of smoke-free legislation, risk reductions were reported in 13 of 17 studies^3,17,27,30,40,55,67,76,90,111,119,120,126,130,134,135,139^ (76.4%) for CVD events, 9 of 17 studies^94,104,111,112,113,123,130,136,146,147,149,154,165,170,171^ (52.9%) for CVD-related hospitalizations, 14 of 18 studies^54,94,104,111,112,113,123,130,136,146,147,149,153,154,165,170,171^ (77.8%) for RSD and RSD symptoms, 8 of 16 studies^90,96,106,107,108,110,116,131,135,138,142,147,148,149,158,170,171,173^ (50.0%) for RSD-related hospitalizations, and 10 of 14 studies^101,111,112,113,115,123,124,139,147,149,165,168^ (71.4%) for risk of adverse birth outcomes. One study^161^ reported an improvement in self-reported health. After the implementation of tax or price increases, all 8 studies^45,62,64,65,100,101,114,125^ found significant reductions in CVD- and RSD-related hospitalizations, RSD and RSD symptoms, sudden infant death syndrome, and adverse birth outcomes. Regarding multicomponent tobacco control policies, both studies^14,141^ assessing risk of CVD-related hospitalizations and RSD and RSD symptoms reported reductions, 2 studies^124,127^ found reductions in mortality (cancer or smoking-attributable), and 1 study^152^ reported a reduction in small for gestational age but not for other birth outcomes, such as perinatal mortality rate, preterm birth, very preterm birth, still birth, neonatal mortality, low birth weight, and very low birth weight. The narrative summary of the associations of free or discounted nicotine replacement therapy, minimum cigarette purchase age law, and tobacco retailer density are presented in eTable 14 in Supplement 1.

**Table.** Narrative Summary of Adverse Health Outcomes Following the Implementation of Different Tobacco Policies

Outcome	Tobacco policy studies, No. (N = 80)								
	Multicomponent tobacco law^a^			Tax or price increase			Smoking-free legislation		
	Positive	Negative	Null	Positive	Negative	Null	Positive	Negative	Null
Cardiovascular event^b^	0	0	0	0	0	0	13	0	4
Hospital admission rates due to cardiovascular diseases^c^	1	0	0	1	0	0	9	0	8
Lung cancer, SIDS, respiratory symptoms and diseases^d^	2	0	0	3	0	0	14	0	4
Hospital admission and discharge rates due to lung diseases^e^	0	0	0	1	0	0	8	2	6
Birth outcomes^f^	1	0	1	3	0	0	10	1	3
Cancer^g^	2	0	0	0	0	0	0	0	0
Health status^h^	0	0	0	0	0	0	1	0	0

Abbreviation: SIDS, sudden infant death syndrome.

^a^ Multicomponent tobacco law means a combination of several policies, such as education on smoking dangers, increases in cigarette taxes, smoke-free air laws, media campaigns, marketing and sales restrictions, lawsuits, cessation treatment programs, and bans on advertising.

^b^ Incidence, prevalence, and mortality of acute myocardial infarction, heart attack, sudden cardiac death, coronary heart disease, stroke, cardiovascular disease, and cerebrovascular disease.

^c^ Hospital admission rates for ischemic heart disease, cardiovascular disease, angina, acute coronary syndrome, coronary heart disease, acute myocardial infarction, heart attack, sudden cardiac death, cerebrovascular disease, and stroke.

^d^ Prevalence and mortality of lung cancer, sudden infant death syndrome, respiratory symptoms and diseases.

^e^ Hospital admission and discharge rates for chronic obstructive pulmonary disease, lower respiratory tract infection, asthma, and bronchitis.

^f^ Risk and rates of infant, neonatal, perinatal, early neonatal, stillbirth, low birth weight, very low birth weight, preterm, very preterm, early term, and small-for-gestational-age births.

^g^ Rates of smoking-attributable mortality and mortality due to cancer.

^h^ Self-reported health status.

## Discussion

To our knowledge, this is the first systematic review and meta-analysis to evaluate the associations of all types of population-level tobacco control policies with health-related outcomes. We found evidence that smoke-free legislation was significantly associated with reductions in the risk of CVD events, RSD events or symptoms, CVD- and RSD-related hospitalizations, and adverse perinatal outcomes. However, the associations of other types of tobacco control policies remain unclear, primarily due to a limited number of primary studies available.

Our analysis showed that smoke-free legislation was associated with approximately 9% to 10% reduced odds of overall CVD events, including CVD incidence, prevalence, and mortality due to acute myocardial infarction, coronary heart disease, cerebrovascular disease (stroke, transient ischemic attack), and sudden cardiac death. In previous meta-analyses investigating the associations with more specific outcomes, smoke-free legislation was found to be associated with reductions in AMI mortality between 8%^17^ and 13%.^20^ Our findings confirm the positive associations of smoke-free legislation with acute myocardial infarction mortality and generally positive outcomes in overall CVD event risk reduction.

Smoke-free legislation was also found to be associated with a 9% reduction in CVD-related hospitalizations. Previous meta-analyses also reported positive associations between the policy and hospitalizations; however, they focused on specific CVD events: a reduction by 15% for coronary events, 16% for cerebrovascular accidents, and 39% for other heart diseases.^24^ Regarding respiratory diseases or symptoms, we found that smoke-free legislation was associated with a 16% to 17% reduction in mortality and a 9% reduction in related hospitalizations, corroborating the findings of a 24% reduction in respiratory disease^24^ and a 19% reduction in respiratory symptoms from previous meta-analyses.^23^ Our results also support the use of smoking bans to improve birth outcomes. We found that smoke-free legislation was associated with a 4% to 9% reduction in the odds of adverse perinatal outcomes. Previous studies have reported mixed findings. One study by Been et al^13^ found evidence of reductions in the risk of preterm birth by10% but not low birth weight.^13^ Another study by Faber et al^15^ reported significant improvements for a range of birth outcomes (eg, 2% reduction in small for gestational age and 10% reduction in the risk of very preterm birth), but not for other birth outcomes. Although tobacco tax policies are significantly associated with reductions in smoking prevalence,^1,11,12,15^ we did not find any significant associations of tax policies with improvements in the health outcomes of interest. At the primary study level, we observed a large heterogeneity in the size and direction of policy outcomes across studies.^60,61^ Recent systematic reviews have found that marketing restrictions and warning labels were associated with decreased tobacco consumption,^1,177,178^ which may in turn be associated with positive health benefits. A 2019 study by Jiang et al^127^ found that a cigarette advertisement ban was associated with reduced tobacco consumption and overall cancer mortality by −1.43 and −1.24 per 100 000 population, respectively.

Enforcement of smoke-free legislation may be associated with a reduction of the risk of adverse health outcomes. Rapid declines in CVD conditions may be associated with decreases in exposure to SHS after the implementation of smoke-free laws.^18^ Even low doses of exposure to toxins in tobacco smoke have been found to increase the risk of CVD conditions through various channels, such as activation of blood platelets, increased arterial stiffness, and others.^98,179,180^ Current evidence for establishing a causal link between tobacco smoke exposure and RSD is only suggestive.^84^ For instance, SHS exposure has been associated with exacerbations among individuals with chronic obstructive pulmonary disease.^181,182^ Moreover, a dose-response association has been demonstrated in relation to the comprehensiveness of smoke-free legislation and the incidence of respiratory disease.^24^ For birth outcomes, active maternal smoking and SHS exposure during pregnancy are known risk factors associated with preterm birth, low birth weight, small for gestational age, stillbirth, and others.^1^ Maternal smoking and exposure to SHS during pregnancy can pose severe developmental health risks to the fetus due to toxic constituents of tobacco smoke that readily penetrate the placenta.^183^

### Limitations

This study has some limitations. First, we found significant between-study heterogeneity. To minimize its influence, we used a random-effects meta-analysis and stratified by individual health outcomes. In our stratified analyses, we found summary ORs consistently less than 1 across several study-level characteristics, except for the country income category. Second, smoke-free legislation policies are implemented to varying degrees (eg, workplaces only, workplaces and restaurants only, or workplaces, restaurants, and bars). Thus, the potential for differential- or dose-response associations is dependent on the comprehensiveness of interventions.^13^ Thus, data were analyzed separately according to whether the bans were comprehensive or partial, but we did not find any significant differences. Third, funnel plot analysis showed some asymmetry for all outcomes, except for mortality due to RSD, suggesting the possibility of publication bias and missing gray literature. To address this issue, we used a trim-and-fill method that can capture all unpublished and gray literature. The results did not vary, suggesting that the association was unaffected by unpublished studies. Fourth, as studies included in the meta-analyses were mainly from high-income countries, the findings might not be generalizable to low- and middle-income countries. We performed a stratified analysis according to income category and found a consistent pooled OR greater than 1 in low- and middle-income countries for all outcomes related to CVD. Fifth, emerging trends in tobacco use, such as the increase in the use of alternative tobacco products (eg, electronic cigarettes) and changing levels of air pollution, may impact the effectiveness of tobacco policies on health outcomes. Additionally, an increasing prevalence of cannabis use, which is often associated with tobacco consumption,^184^ is not fully reflected in this study.

## Conclusions

In this systematic review and meta-analysis, we found that implementation of smoke-free legislation was followed by a significant decrease in multiple adverse health outcomes. The findings support the need to accelerate the uptake of laws restricting smoking in public spaces in efforts to protect people from related cardiovascular, respiratory, and birth health hazards.

## Notes

Supplement 1. **eTable 1.** Synthesis Past Evidence **eTable 2.** PubMed Search Results **eTable 3.** EMBASE Search Results **eTable 4.** Web of Science Search Results **eTable 5.** CINAHL Search Results **eTable 6.** EconLit Search Results **eAppendix 1.** Data Extraction Form **eAppendix 2.** Study Quality Assessment **eFigure 1.** Study Selection Flowchart **eTable 7.** Study Characteristics **eFigure 2.** Forest Plot of Meta-analysis of the Studies Examining the Associations of Smoke-free Legislation With Cardiovascular Disease (CVD), CVD Hospitalization, Respiratory Disease Hospitalization, CVD Mortality, Cancer, Respiratory Symptoms, Birth Weight, and Gestational Age Based on Coefficient Results **eFigure 3.** Meta-analysis of the Association of Smoke-free Legislation With Perinatal Mortality and Adverse Birth Outcomes **eFigure 4.** Forest Plot of Meta-analysis of Studies Examining the Association of Tax or Price Increase With Hospitalization Due to Cardiovascular Disease or Respiratory Symptoms **eFigure 5.** Forest Plot of Meta-analysis of the Studies Examining the Association of Tax or Price Increase With Total Mortality, Cardiovascular Disease Mortality, Cancer Mortality, Respiratory Disease Mortality, Cardiovascular Disease Hospitalization, and Respiratory Disease Hospitalization Based on Coefficient Results **eFigure 6.** Forest Plot of Meta-analysis of Studies Examining the Synergistic Associations of Tobacco Control Program (Combination of Several Tobacco Control Laws) With Cardiovascular Disease Hospitalization, Cardiovascular Disease Mortality, and Cancer Based on Coefficient Results **eAppendix 3.** Sensitivity Analyses **eFigure 7.** Publication Bias Assessment for Cardiovascular Diseases **eFigure 8.** Publication Bias Assessment for Smoke-free Legislation and Respiratory Disease and Symptoms **eFigure 9.** Publication Bias Assessment for Smoke-free Legislation and Perinatal Mortality and Adverse Birth Outcomes **eTable 8.** Summary, Publication Bias, and Trim-and-Fill Estimates **eAppendix 4.** Stratified Analysis **eTable 9.** Stratified Analysis According to Study Design, Country Income Category and Quality Score **eTable 10.** Stratified Analysis According to Comprehensiveness of Smoke-free Legislation **eTable 11.** Narrative Summary of Adverse Health Outcomes Following the Implementation of the Smoking Ban **eTable 12.** Narrative Summary of Adverse Health Outcomes Following the Implementation of Tobacco Law **eTable 13.** Narrative Summary of Adverse Health Outcomes Following the Implementation of an Increase in Cigarette Prices or Cigarette Taxes **eTable 14.** Narrative Summary of Adverse Health Outcomes Following the Implementation of Miscellaneous Policies
eReferences.

Supplement 2.
Data Sharing Statement

## References

[1] Hoffman SJ, Tan C. Overview of systematic reviews on the health-related effects of government tobacco control policies. BMC Public Health. 2015;15:744. doi:10.1186/s12889-015-2041-6 26242915 PMC4526291

[2] Leigh J, Rafiee A, Oancea B, ; GBD 2019 Risk Factors Collaborators. Global burden of 87 risk factors in 204 countries and territories, 1990-2019: a systematic analysis for the Global Burden of Disease Study 2019. Lancet. 2020;396(10258):1223-1249. doi:10.1016/S0140-6736(20)30752-2 33069327 PMC7566194

[3] Jaakkola MS, Piipari R, Jaakkola N, Jaakkola JJ. Environmental tobacco smoke and adult-onset asthma: a population-based incident case-control study. Am J Public Health. 2003;93(12):2055-2060. doi:10.2105/AJPH.93.12.2055 14652334 PMC1448152

[4] Kawachi I, Colditz GA, Speizer FE, . A prospective study of passive smoking and coronary heart disease. Circulation. 1997;95(10):2374-2379. doi:10.1161/01.CIR.95.10.2374 9170399

[5] Skorge TD, Eagan TM, Eide GE, Gulsvik A, Bakke PS. The adult incidence of asthma and respiratory symptoms by passive smoking in uterus or in childhood. Am J Respir Crit Care Med. 2005;172(1):61-66. doi:10.1164/rccm.200409-1158OC 15805186

[6] Steenland K, Thun M, Lally C, Heath C Jr. Environmental tobacco smoke and coronary heart disease in the American Cancer Society CPS-II cohort. Circulation. 1996;94(4):622-628. doi:10.1161/01.CIR.94.4.622 8772680

[7] Vineis P, Airoldi L, Veglia F, . Environmental tobacco smoke and risk of respiratory cancer and chronic obstructive pulmonary disease in former smokers and never smokers in the EPIC prospective study. BMJ. 2005;330(7486):277. doi:10.1136/bmj.38327.648472.82 15681570 PMC548173

[8] Zhong L, Goldberg MS, Parent ME, Hanley JA. Exposure to environmental tobacco smoke and the risk of lung cancer: a meta-analysis. Lung Cancer. 2000;27(1):3-18. doi:10.1016/S0169-5002(99)00093-8 10672779

[9] Bonita R, Duncan J, Truelsen T, Jackson RT, Beaglehole R. Passive smoking as well as active smoking increases the risk of acute stroke. Tob Control. 1999;8(2):156-160. doi:10.1136/tc.8.2.156 10478399 PMC1759715

[10] World Health Organization. WHO Framework Convention on Tobacco Control. World Health Organization; 2003.

[11] Centers for Disease Control and Prevention. Best Practices for Comprehensive Tobacco Control Programs—2014. US Department of Health and Human Services, Centers for Disease Control and Prevention, National Center for Chronic Disease Prevention and Health Promotion, Office on Smoking and Health; 2014.

[12] Task Force on Community Preventive Services. Strategies for reducing exposure to environmental tobacco smoke, increasing tobacco-use cessation, and reducing initiation in communities and health-care systems: a report on recommendations of the Task Force on Community Preventive Services. MMWR Recomm Rep. 2000;49(RR-12):1-11. 15580784

[13] Been JV, Nurmatov UB, Cox B, Nawrot TS, van Schayck CP, Sheikh A. Effect of smoke-free legislation on perinatal and child health: a systematic review and meta-analysis. Lancet. 2014;383(9928):1549-1560. doi:10.1016/S0140-6736(14)60082-9 24680633

[14] Dinno A, Glantz S. Clean indoor air laws immediately reduce heart attacks. Prev Med. 2007;45(1):9-11. doi:10.1016/j.ypmed.2007.03.013 17499350 PMC2693058

[15] Faber T, Kumar A, Mackenbach JP, . Effect of tobacco control policies on perinatal and child health: a systematic review and meta-analysis. Lancet Public Health. 2017;2(9):e420-e437. doi:10.1016/S2468-2667(17)30144-5 28944313 PMC5592249

[16] Frazer K, Callinan JE, McHugh J, . Legislative smoking bans for reducing harms from secondhand smoke exposure, smoking prevalence and tobacco consumption. Cochrane Database Syst Rev. 2016;2(2):CD005992. doi:10.1002/14651858.CD005992.pub3 26842828 PMC6486282

[17] Gao M, Li Y, Wang F, . The effect of smoke-free legislation on the mortality rate of acute myocardial infarction: a meta-analysis. BMC Public Health. 2019;19(1):1269. doi:10.1186/s12889-019-7408-7 31533693 PMC6749716

[18] Jones MR, Barnoya J, Stranges S, Losonczy L, Navas-Acien A. Cardiovascular events following smoke-free legislations: an updated systematic review and meta-analysis. Curr Environ Health Rep. 2014;1(3):239-249. doi:10.1007/s40572-014-0020-1 25328861 PMC4198310

[19] Lightwood JM, Glantz SA. Declines in acute myocardial infarction after smoke-free laws and individual risk attributable to secondhand smoke. Circulation. 2009;120(14):1373-1379. doi:10.1161/CIRCULATIONAHA.109.870691 19770392 PMC2967202

[20] Lin H, Wang H, Wu W, Lang L, Wang Q, Tian L. The effects of smoke-free legislation on acute myocardial infarction: a systematic review and meta-analysis. BMC Public Health. 2013;13:529. doi:10.1186/1471-2458-13-529 23721370 PMC3671962

[21] Mackay DF, Irfan MO, Haw S, Pell JP. Meta-analysis of the effect of comprehensive smoke-free legislation on acute coronary events. Heart. 2010;96(19):1525-1530. doi:10.1136/hrt.2010.199026 20736203

[22] Meyers DG, Neuberger JS, He J. Cardiovascular effect of bans on smoking in public places: a systematic review and meta-analysis. J Am Coll Cardiol. 2009;54(14):1249-1255. doi:10.1016/j.jacc.2009.07.022 19778665

[23] Rando-Matos Y, Pons-Vigués M, López MJ, . Smokefree legislation effects on respiratory and sensory disorders: a systematic review and meta-analysis. PLoS One. 2017;12(7):e0181035. doi:10.1371/journal.pone.0181035 28759596 PMC5536320

[24] Tan CE, Glantz SA. Association between smoke-free legislation and hospitalizations for cardiac, cerebrovascular, and respiratory diseases: a meta-analysis. Circulation. 2012;126(18):2177-2183. doi:10.1161/CIRCULATIONAHA.112.121301 23109514 PMC3501404

[25] Faber T, Kumar A, Mackenbach JP, . Tobacco control policies and perinatal and child health: a systematic review and meta-analysis. Tob Induc Dis. 2018;16(1):157. doi:10.18332/tid/83921

[26] Radó MK, Laverty AA, Hone T, . Cigarette taxation and neonatal and infant mortality: a longitudinal analysis of 159 countries. PLOS Glob Public Health. 2022;2(3):e0000042. doi:10.1371/journal.pgph.0000042 36962262 PMC10021450

[27] Wells GSB, O’Connell D, Peterson J, . The Newcastle-Ottawa Scale (NOS) for assessing the quality of nonrandomized studies in meta-analyses. Accessed June 2, 2023. https://www.ohri.ca/programs/clinical_epidemiology/oxford.asp

[28] Wheldon MC, Raftery AE, Clark SJ, Gerland P. Reconstructing past populations with uncertainty from fragmentary data. J Am Stat Assoc. 2013;108(501):96-110. doi:10.1080/01621459.2012.737729 23579202 PMC3613971

[29] Schmidt CO, Kohlmann T. When to use the odds ratio or the relative risk? Int J Public Health. 2008;53(3):165-167. doi:10.1007/s00038-008-7068-3 19127890

[30] Sterne JAC, Egger M. Regression Methods to Detect Publication and Other Bias in Meta-analysis. In: Rothstein HR, Sutton AJ, Borenstein M, . Publication Bias in Meta-Analysis. Wiley; 2006:99-110. doi:10.1002/0470870168.ch6

[31] Duval S, Tweedie R. Trim and fill: a simple funnel-plot-based method of testing and adjusting for publication bias in meta-analysis. Biometrics. 2000;56(2):455-463. doi:10.1111/j.0006-341X.2000.00455.x 10877304

[32] Agüero F, Dégano IR, Subirana I, . Impact of a partial smoke-free legislation on myocardial infarction incidence, mortality and case-fatality in a population-based registry: the REGICOR Study. PLoS One. 2013;8(1):e53722. doi:10.1371/journal.pone.0053722 23372663 PMC3553094

[33] Amaral M. The effect of local smoking ordinances on fetal development: evidence from California. Accessed June 2, 2023. https://citeseerx.ist.psu.edu/viewdoc/download;jsessionid=9D1C3595C23F1A5F40D0475285323867?doi=10.1.1.503.5200&rep=rep1&type=pdf

[34] Bakolis I, Kelly R, Fecht D, . Protective effects of smoke-free legislation on birth outcomes in England: a regression discontinuity design. Epidemiology. 2016;27(6):810-818. doi:10.1097/EDE.0000000000000534 27428672 PMC5424880

[35] Bannon F, Devlin A, McElwee G, Gavin A. Greater gains from smoke-free legislation for non-smoking bar staff in Belfast. Eur J Public Health. 2009;19(6):638-643. doi:10.1093/eurpub/ckp087 19567658

[36] Barnett R, Pearce J, Moon G, Elliott J, Barnett P. Assessing the effects of the introduction of the New Zealand Smokefree Environment Act 2003 on acute myocardial infarction hospital admissions in Christchurch, New Zealand. Aust N Z J Public Health. 2009;33(6):515-520. doi:10.1111/j.1753-6405.2009.00446.x 20078567

[37] Barone-Adesi F, Gasparrini A, Vizzini L, Merletti F, Richiardi L. Effects of Italian smoking regulation on rates of hospital admission for acute coronary events: a country-wide study. PLoS One. 2011;6(3):e17419. doi:10.1371/journal.pone.0017419 21399685 PMC3047543

[38] Barone-Adesi F, Vizzini L, Merletti F, Richiardi L. Short-term effects of Italian smoking regulation on rates of hospital admission for acute myocardial infarction. Eur Heart J. 2006;27(20):2468-2472. doi:10.1093/eurheartj/ehl201 16940340

[39] Barrio G, Belza MJ, Carmona R, Hoyos J, Ronda E, Regidor E. The limits of single-group interrupted time series analysis in assessing the impact of smoke-free laws on short-term mortality. Int J Drug Policy. 2019;73:112-120. doi:10.1016/j.drugpo.2019.07.018 31470256

[40] Bartecchi C, Alsever RN, Nevin-Woods C, . Reduction in the incidence of acute myocardial infarction associated with a citywide smoking ordinance. Circulation. 2006;114(14):1490-1496. doi:10.1161/CIRCULATIONAHA.106.615245 17000911

[41] Bartholomew KS, Abouk R. The effect of local smokefree regulations on birth outcomes and prenatal smoking. Matern Child Health J. 2016;20(7):1526-1538. doi:10.1007/s10995-016-1952-x 26987859

[42] Basel P, Bartelson BB, Le Lait MC, Krantz MJ. The effect of a statewide smoking ordinance on acute myocardial infarction rates. Am J Med. 2014;127(1):94.e1-94.e6. doi:10.1016/j.amjmed.2013.09.014 24384105

[43] Been JV, Mackay DF, Millett C, Pell JP, van Schayck OC, Sheikh A. Impact of smoke-free legislation on perinatal and infant mortality: a national quasi-experimental study. Sci Rep. 2015;5:13020. doi:10.1038/srep13020 26268789 PMC4534797

[44] Been JV, Millett C, Lee JT, van Schayck CP, Sheikh A. Smoke-free legislation and childhood hospitalisations for respiratory tract infections. Eur Respir J. 2015;46(3):697-706. doi:10.1183/09031936.00014615 26022951

[45] Been JV, Szatkowski L, van Staa TP, . Smoke-free legislation and the incidence of paediatric respiratory infections and wheezing/asthma: interrupted time series analyses in the four UK nations. Sci Rep. 2015;5:15246. doi:10.1038/srep15246 26463498 PMC4604467

[46] Bowser D, Canning D, Okunogbe A. The impact of tobacco taxes on mortality in the USA, 1970-2005. Tob Control. 2016;25(1):52-59. 25352561 10.1136/tobaccocontrol-2014-051666

[47] Bruintjes G, Bartelson BB, Hurst P, Levinson AH, Hokanson JE, Krantz MJ. Reduction in acute myocardial infarction hospitalization after implementation of a smoking ordinance. Am J Med. 2011;124(7):647-654. doi:10.1016/j.amjmed.2011.02.022 21683831

[48] Cesaroni G, Forastiere F, Agabiti N, Valente P, Zuccaro P, Perucci CA. Effect of the Italian smoking ban on population rates of acute coronary events. Circulation. 2008;117(9):1183-1188. doi:10.1161/CIRCULATIONAHA.107.729889 18268149

[49] Ciaccio CE, Gurley-Calvez T, Shireman TI. Indoor tobacco legislation is associated with fewer emergency department visits for asthma exacerbation in children. Ann Allergy Asthma Immunol. 2016;117(6):641-645. doi:10.1016/j.anai.2016.10.005 27979021 PMC5166981

[50] Cox B, Vangronsveld J, Nawrot TS. Impact of stepwise introduction of smoke-free legislation on population rates of acute myocardial infarction deaths in Flanders, Belgium. Heart. 2014;100(18):1430-1435. doi:10.1136/heartjnl-2014-305613 25147283

[51] Croghan IT, Ebbert JO, Hays JT, . Impact of a countywide smoke-free workplace law on emergency department visits for respiratory diseases: a retrospective cohort study. BMC Pulm Med. 2015;15:6. doi:10.1186/1471-2466-15-6 25608660 PMC4417313

[52] Carrión-Valero F, Quiles-Izquierdo J, González-Monte C, . Impact of the 2005 and 2010 Spanish smoking laws on hospital admissions for tobacco-related diseases in Valencia, Spain. Public Health. 2020;180:29-37. doi:10.1016/j.puhe.2019.10.016 31838343

[53] Dilley JA, Harris JR, Boysun MJ, Reid TR. Program, policy, and price interventions for tobacco control: quantifying the return on investment of a state tobacco control program. Am J Public Health. 2012;102(2):e22-e28. doi:10.2105/AJPH.2011.300506 22390458 PMC3484005

[54] Dove MS, Dockery DW, Connolly GN. Smoke-free air laws and asthma prevalence, symptoms, and severity among nonsmoking youth. Pediatrics. 2011;127(1):102-109. doi:10.1542/peds.2010-1532 21149426 PMC3375465

[55] Fernández E, Fu M, Pascual JA, ; Spanish Smoking Law Evaluation Group. Impact of the Spanish smoking law on exposure to second-hand smoke and respiratory health in hospitality workers: a cohort study. PLoS One. 2009;4(1):e4244. doi:10.1371/journal.pone.0004244 19165321 PMC2621339

[56] Ferrante D, Linetzky B, Virgolini M, Schoj V, Apelberg B. Reduction in hospital admissions for acute coronary syndrome after the successful implementation of 100% smoke-free legislation in Argentina: a comparison with partial smoking restrictions. Tob Control. 2012;21(4):402-406. doi:10.1136/tc.2010.042325 21602536

[57] Fichtenberg CM, Glantz SA. Association of the California Tobacco Control Program with declines in cigarette consumption and mortality from heart disease. N Engl J Med. 2000;343(24):1772-1777. doi:10.1056/NEJM200012143432406 11114317

[58] Gasparrini A, Gorini G, Barchielli A. On the relationship between smoking bans and incidence of acute myocardial infarction. Eur J Epidemiol. 2009;24(10):597-602. doi:10.1007/s10654-009-9377-0 19649714

[59] Hahn EJ, Rayens MK, Burkhart PV, Moser DK. Smoke-free laws, gender, and reduction in hospitalizations for acute myocardial infarction. Public Health Rep. 2011;126(6):826-833. doi:10.1177/003335491112600608 22043098 PMC3185318

[60] Hatoun J, Davis-Plourde K, Penti B, Cabral H, Kazis L. Tobacco control laws and pediatric asthma. Pediatrics. 2018;141(suppl 1):S130-S136. doi:10.1542/peds.2017-1026P 29292313

[61] Hawkins SS, Hristakeva S, Gottlieb M, Baum CF. Reduction in emergency department visits for children’s asthma, ear infections, and respiratory infections after the introduction of state smoke-free legislation. Prev Med. 2016;89:278-285. doi:10.1016/j.ypmed.2016.06.005 27283094 PMC8323994

[62] Head P, Jackson BE, Bae S, Cherry D. Hospital discharge rates before and after implementation of a city-wide smoking ban in a Texas city, 2004-2008. Prev Chronic Dis. 2012;9:E179. doi:10.5888/pcd9.120079 23270668 PMC3534134

[63] Ho V, Ross JS, Steiner CA, . A nationwide assessment of the association of smoking bans and cigarette taxes with hospitalizations for acute myocardial infarction, heart failure, and pneumonia. Med Care Res Rev. 2017;74(6):687-704. doi:10.1177/1077558716668646 27624634 PMC5665160

[64] Humair JP, Garin N, Gerstel E, . Acute respiratory and cardiovascular admissions after a public smoking ban in Geneva, Switzerland. PLoS One. 2014;9(3):e90417. doi:10.1371/journal.pone.0090417 24599156 PMC3944023

[65] Hurt RD, Weston SA, Ebbert JO, . Myocardial infarction and sudden cardiac death in Olmsted County, Minnesota, before and after smoke-free workplace laws. Arch Intern Med. 2012;172(21):1635-1641. doi:10.1001/2013.jamainternmed.46 23108571 PMC3615114

[66] Jan C, Lee M, Roa R, Herrera V, Politis M, Motta J. The association of tobacco control policies and the risk of acute myocardial infarction using hospital admissions data. PLoS One. 2014;9(2):e88784. doi:10.1371/journal.pone.0088784 24520421 PMC3919809

[67] Juster HR, Loomis BR, Hinman TM, . Declines in hospital admissions for acute myocardial infarction in New York state after implementation of a comprehensive smoking ban. Am J Public Health. 2007;97(11):2035-2039. doi:10.2105/AJPH.2006.099994 17901438 PMC2040364

[68] Kabir Z, Clarke V, Conroy R, McNamee E, Daly S, Clancy L. Low birthweight and preterm birth rates 1 year before and after the Irish workplace smoking ban. BJOG. 2009;116(13):1782-1787. doi:10.1111/j.1471-0528.2009.02374.x 19832830

[69] Kalkhoran S, Sebrié EM, Sandoya E, Glantz SA. Effect of Uruguay’s national 100% smokefree law on emergency visits for bronchospasm. Am J Prev Med. 2015;49(1):85-88. doi:10.1016/j.amepre.2014.12.009 25997906 PMC4476915

[70] Kent BD, Sulaiman I, Nicholson TT, Lane SJ, Moloney ED. Acute pulmonary admissions following implementation of a national workplace smoking ban. Chest. 2012;142(3):673-679. doi:10.1378/chest.11-2757 22383660

[71] Larsson M, Boëthius G, Axelsson S, Montgomery SM. Exposure to environmental tobacco smoke and health effects among hospitality workers in Sweden—before and after the implementation of a smoke-free law. Scand J Work Environ Health. 2008;34(4):267-277. doi:10.5271/sjweh.1243 18815714

[72] Lemstra M, Neudorf C, Opondo J. Implications of a public smoking ban. Can J Public Health. 2008;99(1):62-65. doi:10.1007/BF03403743 18435394 PMC6975881

[73] Liu A, Guzman Castillo M, Capewell S, Lucy J, O’Flaherty M. Reduction in myocardial infarction admissions in Liverpool after the smoking ban: potential socioeconomic implications for policymaking. BMJ Open. 2013;3(11):e003307. doi:10.1136/bmjopen-2013-003307 24282240 PMC3845049

[74] Loomis BR, Juster HR. Association of indoor smoke-free air laws with hospital admissions for acute myocardial infarction and stroke in three states. J Environ Public Health. 2012;2012:589018. doi:10.1155/2012/589018 22778759 PMC3388299

[75] Mayne SL, Widome R, Carroll AJ, . Longitudinal associations of smoke-free policies and incident cardiovascular disease: CARDIA study. Circulation. 2018;138(6):557-566. doi:10.1161/CIRCULATIONAHA.117.032302 29735485 PMC6202173

[76] McAlister AL, Huang P, Ramirez AG, Harrist RB, Fonseca VP. Reductions in cigarette smoking and acute myocardial infarction mortality in Jefferson County, Texas. Am J Public Health. 2010;100(12):2391-2392. doi:10.2105/AJPH.2010.192211 20966365 PMC2978173

[77] McKinnon B, Auger N, Kaufman JS. The impact of smoke-free legislation on educational differences in birth outcomes. J Epidemiol Community Health. 2015;69(10):937-943. doi:10.1136/jech-2015-205779 25987722

[78] Millett C, Lee JT, Laverty AA, Glantz SA, Majeed A. Hospital admissions for childhood asthma after smoke-free legislation in England. Pediatrics. 2013;131(2):e495-e501. doi:10.1542/peds.2012-2592 23339216 PMC4528337

[79] Moraros J, Bird Y, Chen S, . The impact of the 2002 Delaware smoking ordinance on heart attack and asthma. Int J Environ Res Public Health. 2010;7(12):4169-4178. doi:10.3390/ijerph7124169 21318001 PMC3037047

[80] Page RL II, Slejko JF, Libby AM. A citywide smoking ban reduced maternal smoking and risk for preterm births: a Colorado natural experiment. J Womens Health (Larchmt). 2012;21(6):621-627. doi:10.1089/jwh.2011.3305 22401497

[81] Patanavanich R, Glantz SA. Association between tobacco control policies and hospital admissions for acute myocardial infarction in Thailand, 2006-2017: a time series analysis. PLoS One. 2020;15(12):e0242570. doi:10.1371/journal.pone.0242570 33264315 PMC7710088

[82] Polus S, Burns J, Hoffmann S, . Interrupted time series study found mixed effects of the impact of the Bavarian smoke-free legislation on pregnancy outcomes. Sci Rep. 2021;11(1):4209. doi:10.1038/s41598-021-83774-0 33603103 PMC7892567

[83] Séguret F, Ferreira C, Cambou JP, Carrière I, Thomas D. Changes in hospitalization rates for acute coronary syndrome after a two-phase comprehensive smoking ban. Eur J Prev Cardiol. 2014;21(12):1575-1582. doi:10.1177/2047487313500569 23918841

[84] Stallings-Smith S, Goodman P, Kabir Z, Clancy L, Zeka A. Socioeconomic differentials in the immediate mortality effects of the national Irish smoking ban. PLoS One. 2014;9(6):e98617. doi:10.1371/journal.pone.0098617 24887027 PMC4041857

[85] Stallings-Smith S, Zeka A, Goodman P, Kabir Z, Clancy L. Reductions in cardiovascular, cerebrovascular, and respiratory mortality following the national irish smoking ban: interrupted time-series analysis. PLoS One. 2013;8(4):e62063. doi:10.1371/journal.pone.0062063 23637964 PMC3634756

[86] Villalbí JR, Sánchez E, Benet J, ; Barcelona Group for Smoking Regulation Policies Evaluation. The extension of smoke-free areas and acute myocardial infarction mortality: before and after study. BMJ Open. 2011;1(1):e000067. doi:10.1136/bmjopen-2011-000067 22021746 PMC3191414

[87] Xiao H, Qi F, Jia X, . Impact of Qingdao’s smoke-free legislation on hospitalizations and mortality from acute myocardial infarction and stroke: an interrupted time-series analysis. Addiction. 2020;115(8):1561-1570. doi:10.1111/add.14970 31961014

[88] Xiao H, Zhang H, Wang D, . Impact of smoke-free legislation on acute myocardial infarction and stroke mortality: Tianjin, China, 2007-2015. Tob Control. 2020;29(1):61-67. doi:10.1136/tobaccocontrol-2018-054477 30692165 PMC6952839

[89] Yang YN, Huang YT, Yang CY. Effects of a national smoking ban on hospital admissions for cardiovascular diseases: a time-series analysis in Taiwan. J Toxicol Environ Health A. 2017;80(10-12):562-568. doi:10.1080/15287394.2017.1367085 28880815

[90] Alsever R, Thomas W, Nevin-Woods C, ; Centers for Disease Control and Prevention (CDC). Reduced hospitalizations for acute myocardial infarction after implementation of a smoke-free ordinance—City of Pueblo, Colorado, 2002-2006. MMWR Morb Mortal Wkly Rep. 2009;57(51):1373-1377. 19116606

[91] Sebrié EM, Sandoya E, Hyland A, Bianco E, Glantz SA, Cummings KM. Hospital admissions for acute myocardial infarction before and after implementation of a comprehensive smoke-free policy in Uruguay. Tob Control. 2013;22(e1):e16-e20. doi:10.1136/tobaccocontrol-2011-050134 22337557 PMC3374906

[92] de Korte-de Boer D, Kotz D, Viechtbauer W, . Effect of smoke-free legislation on the incidence of sudden circulatory arrest in the Netherlands. Heart 2012;98:995-9. Heart. 2012;98(22):1680. doi:10.1136/heartjnl-2012-302752 22668867

[93] Abe TMO, Scholz J, de Masi E, Nobre MRC, Filho RK. Decrease in mortality rate and hospital admissions for acute myocardial infarction after the enactment of the smoking ban law in São Paulo city, Brazil. Tob Control. 2017;26(6):656-662. doi:10.1136/tobaccocontrol-2016-053261 27794066

[94] Abreu D, Sousa P, Matias-Dias C, Pinto FJ. Longitudinal impact of the smoking ban legislation in acute coronary syndrome admissions. Biomed Res Int. 2017;2017:6956941. doi:10.1155/2017/6956941 28265574 PMC5318631

[95] Adams EK, Markowitz S, Dietz PM, Tong VT. Expansion of Medicaid covered smoking cessation services: maternal smoking and birth outcomes. Medicare Medicaid Res Rev. 2013;3(3):E1-E23. doi:10.5600/mmrr.003.03.a02 24753968 PMC3983727

[96] Allwright S, Paul G, Greiner B, . Legislation for smoke-free workplaces and health of bar workers in Ireland: before and after study. BMJ. 2005;331(7525):1117. doi:10.1136/bmj.38636.499225.55 16230313 PMC1283274

[97] Ayres JG, Semple S, MacCalman L, . Bar workers’ health and environmental tobacco smoke exposure (BHETSE): symptomatic improvement in bar staff following smoke-free legislation in Scotland. Occup Environ Med. 2009;66(5):339-346. doi:10.1136/oem.2008.040311 19208693

[98] Barnoya J, Glantz SA. Cardiovascular effects of secondhand smoke: nearly as large as smoking. Circulation. 2005;111(20):2684-2698. doi:10.1161/CIRCULATIONAHA.104.492215 15911719

[99] Barr CD, Diez DM, Wang Y, Dominici F, Samet JM. Comprehensive smoking bans and acute myocardial infarction among Medicare enrollees in 387 US counties: 1999-2008. Am J Epidemiol. 2012;176(7):642-648. doi:10.1093/aje/kws267 22986145 PMC3530376

[100] Bharadwaj P, Johnsen JV, Løken KV. Smoking bans, maternal smoking and birth outcomes. J Public Econ. 2014;115:72-93. doi:10.1016/j.jpubeco.2014.04.008

[101] Bianchi M, Campi R, Bonati M. Smoke-free legislation and asthma. N Engl J Med. 2011;364(1):87-88. doi:10.1056/NEJMc1011724 21208120

[102] Bonetti PO, Trachsel LD, Kuhn MU, . Incidence of acute myocardial infarction after implementation of a public smoking ban in Graubünden, Switzerland: two year follow-up. Swiss Med Wkly. 2011;141:w13206. doi:10.4414/smw.2011.13206 21623477

[103] Carrión-Valero F, Quiles-Izquierdo J, González-Monte C, . Association between a comprehensive smoking ban and hospitalization for acute myocardial infarction: an observational study in the Autonomous Community of Valencia, Spain. Rev Port Cardiol (Engl Ed). 2020;39(2):77-84. doi:10.1016/j.repc.2019.04.009 32291119

[104] Cronin EM, Kearney PM, Kearney PP, Sullivan P, Perry IJ; Coronary Heart Attack Ireland Registry (CHAIR) Working Group. Impact of a national smoking ban on hospital admission for acute coronary syndromes: a longitudinal study. Clin Cardiol. 2012;35(4):205-209. doi:10.1002/clc.21014 22278857 PMC6652533

[105] Dove MS, Dockery DW, Mittleman MA, . The impact of Massachusetts’ smoke-free workplace laws on acute myocardial infarction deaths. Am J Public Health. 2010;100(11):2206-2212. doi:10.2105/AJPH.2009.189662 20864706 PMC2951939

[106] Durham AD, Bergier S, Morisod X, . Improved health of hospitality workers after a Swiss cantonal smoking ban. Swiss Med Wkly. 2011;141:w13317. doi:10.4414/smw.2011.13317 22252843

[107] Eagan TM, Hetland J, Aarø LE. Decline in respiratory symptoms in service workers five months after a public smoking ban. Tob Control. 2006;15(3):242-246. doi:10.1136/tc.2005.015479 16728756 PMC2564667

[108] Eisner MD, Smith AK, Blanc PD. Bartenders’ respiratory health after establishment of smoke-free bars and taverns. JAMA. 1998;280(22):1909-1914. doi:10.1001/jama.280.22.1909 9851475

[109] Evans WN, Ringel JS. Can higher cigarette taxes improve birth outcomes? J Public Econ. 1999;72(1):135-154. doi:10.1016/S0047-2727(98)00090-5

[110] Farrelly MC, Nonnemaker JM, Chou R, Hyland A, Peterson KK, Bauer UE. Changes in hospitality workers’ exposure to secondhand smoke following the implementation of New York’s smoke-free law. Tob Control. 2005;14(4):236-241. doi:10.1136/tc.2004.008839 16046685 PMC1748080

[111] Galán I, Simón L, Boldo E, . Changes in hospitalizations for chronic respiratory diseases after two successive smoking bans in Spain. PLoS One. 2017;12(5):e0177979. doi:10.1371/journal.pone.0177979 28542337 PMC5443522

[112] Galán I, Simón L, Boldo E, . Impact of 2 successive smoking bans on hospital admissions for cardiovascular diseases in Spain. Rev Esp Cardiol (Engl Ed). 2018;71(9):726-734. doi:10.1016/j.rec.2017.10.055 29673904

[113] Galán I, Simón L, Flores V, . Assessing the effects of the Spanish partial smoking ban on cardiovascular and respiratory diseases: methodological issues. BMJ Open. 2015;5(12):e008892. doi:10.1136/bmjopen-2015-008892 26628524 PMC4679921

[114] Gao J, Baughman RA. Do smoking bans improve infant health: evidence from U.S. Births: 1995–2009. East Econ J. 2017;43(3):472-495. doi:10.1057/s41302-016-0010-0

[115] Gaudreau K, Sanford CJ, Cheverie C, McClure C. The effect of a smoking ban on hospitalization rates for cardiovascular and respiratory conditions in Prince Edward Island, Canada. PLoS One. 2013;8(3):e56102. doi:10.1371/journal.pone.0056102 23520450 PMC3592861

[116] Goodman P, Agnew M, McCaffrey M, Paul G, Clancy L. Effects of the Irish smoking ban on respiratory health of bar workers and air quality in Dublin pubs. Am J Respir Crit Care Med. 2007;175(8):840-845. doi:10.1164/rccm.200608-1085OC 17204724

[117] Gupta R, Luo J, Anderson RH, Ray A. Clean indoor air regulation and incidence of hospital admissions for acute coronary syndrome in Kanawha County, West Virginia. Prev Chronic Dis. 2011;8(4):A77. 21672401 PMC3136970

[118] Hajdu T, Hajdu G. Smoking ban and health at birth: evidence from Hungary. Econ Hum Biol. 2018;30:37-47. doi:10.1016/j.ehb.2018.05.003 29908431

[119] Hankins S, Tarasenko Y. Do smoking bans improve neonatal health? Health Serv Res. 2016;51(5):1858-1878. doi:10.1111/1475-6773.12451 26841359 PMC5034216

[120] Hawkins SS, Baum CF. Impact of state tobacco control policies on birth defects. Prev Med. 2019;127:105791. doi:10.1016/j.ypmed.2019.105791 31398414

[121] Hawkins SS, Baum CF. The downstream effects of state tobacco control policies on maternal smoking during pregnancy and birth outcomes. Drug Alcohol Depend. 2019;205:107634. doi:10.1016/j.drugalcdep.2019.107634 31669802

[122] Hawkins SS, Baum CF, Oken E, Gillman MW. Associations of tobacco control policies with birth outcomes. JAMA Pediatr. 2014;168(11):e142365. doi:10.1001/jamapediatrics.2014.2365 25365250 PMC4240616

[123] Herman PM, Walsh ME. Hospital admissions for acute myocardial infarction, angina, stroke, and asthma after implementation of Arizona’s comprehensive statewide smoking ban. Am J Public Health. 2011;101(3):491-496. doi:10.2105/AJPH.2009.179572 20466955 PMC3036684

[124] Holford TR, Meza R, Warner KE, . Tobacco control and the reduction in smoking-related premature deaths in the United States, 1964-2012. JAMA. 2014;311(2):164-171. doi:10.1001/jama.2013.285112 24399555 PMC4056770

[125] Hone T, Szklo AS, Filippidis FT, . Smoke-free legislation and neonatal and infant mortality in Brazil: longitudinal quasi-experimental study. Tob Control. 2020;29(3):312-319. 31152114 10.1136/tobaccocontrol-2019-054923

[126] Jarlenski M, Bleich SN, Bennett WL, Stuart EA, Barry CL. Medicaid enrollment policy increased smoking cessation among pregnant women but had no impact on birth outcomes. Health Aff (Millwood). 2014;33(6):997-1005. doi:10.1377/hlthaff.2013.1167 24889949 PMC4248559

[127] Jiang H, Livingston M, Room R, Gan Y, English D, Chenhall R. Can public health policies on alcohol and tobacco reduce a cancer epidemic: Australia’s experience. BMC Med. 2019;17(1):213. doi:10.1186/s12916-019-1453-z 31771596 PMC6880568

[128] Johnson EL, Beal JR. Impact of a comprehensive smoke-free law following a partial smoke-free law on incidence of heart attacks at a rural community hospital. Nicotine Tob Res. 2013;15(3):745-747. doi:10.1093/ntr/nts216 23024248

[129] Kabir Z, Daly S, Clarke V, Keogan S, Clancy L. Smoking ban and small-for-gestational age births in Ireland. PLoS One. 2013;8(3):e57441. doi:10.1371/journal.pone.0057441 23555561 PMC3608631

[130] Khuder SA, Milz S, Jordan T, Price J, Silvestri K, Butler P. The impact of a smoking ban on hospital admissions for coronary heart disease. Prev Med. 2007;45(1):3-8. doi:10.1016/j.ypmed.2007.03.011 17482249

[131] Kim J, Kwon HJ, Lee K, . Air quality, biomarker levels, and health effects on staff in Korean restaurants and pubs before and after a smoking ban. Nicotine Tob Res. 2015;17(11):1337-1346. doi:10.1093/ntr/ntv012 25649052

[132] Kong AY, Baggett CD, Gottfredson NC, Ribisl KM, Delamater PL, Golden SD. Associations of tobacco retailer availability with chronic obstructive pulmonary disease related hospital outcomes, United States, 2014. Health Place. 2021;67:102464. doi:10.1016/j.healthplace.2020.102464 33276261 PMC7854476

[133] Landers G. The impact of smoke-free laws on asthma discharges: a multistate analysis. Am J Public Health. 2014;104(2):e74-e79. doi:10.2105/AJPH.2013.301697 24328638 PMC3935700

[134] Lee SL, Wong WH, Lau YL. Smoke-free legislation reduces hospital admissions for childhood lower respiratory tract infection. Tob Control. 2016;25(e2):e90-e94. doi:10.1136/tobaccocontrol-2015-052541 26769122

[135] Li X, Gao J, Zhang Z, . Lessons from an evaluation of a provincial-level smoking control policy in Shanghai, China. PLoS One. 2013;8(9):e74306. doi:10.1371/journal.pone.0074306 24058544 PMC3769237

[136] Lippert WC, Gustat J. Clean indoor air acts reduce the burden of adverse cardiovascular outcomes. Public Health. 2012;126(4):279-285. doi:10.1016/j.puhe.2012.01.005 22342076

[137] Ma ZQ, Kuller LH, Fisher MA, Ostroff SM. Use of interrupted time-series method to evaluate the impact of cigarette excise tax increases in Pennsylvania, 2000-2009. Prev Chronic Dis. 2013;10:E169. doi:10.5888/pcd10.120268 24135393 PMC3804017

[138] MacCalman L, Semple S, Galea KS, . The relationship between workers’ self-reported changes in health and their attitudes towards a workplace intervention: lessons from smoke-free legislation across the UK hospitality industry. BMC Public Health. 2012;12:324. doi:10.1186/1471-2458-12-324 22551087 PMC3407478

[139] Mackay D, Haw S, Ayres JG, Fischbacher C, Pell JP. Smoke-free legislation and hospitalizations for childhood asthma. N Engl J Med. 2010;363(12):1139-1145. doi:10.1056/NEJMoa1002861 20843248

[140] Mackay DF, Haw S, Newby DE, . Impact of Scotland’s comprehensive, smoke-free legislation on stroke. PLoS One. 2013;8(5):e62597. doi:10.1371/journal.pone.0062597 23667497 PMC3648581

[141] Mackay DF, Nelson SM, Haw SJ, Pell JP. Impact of Scotland’s smoke-free legislation on pregnancy complications: retrospective cohort study. PLoS Med. 2012;9(3):e1001175. doi:10.1371/journal.pmed.1001175 22412353 PMC3295815

[142] Madureira J, Mendes A, Teixeira JP. Evaluation of a smoke-free law on indoor air quality and on workers’ health in Portuguese restaurants. J Occup Environ Hyg. 2014;11(4):201-209. doi:10.1080/15459624.2013.852279 24579749

[143] Mallma P, Carcamo C, Kaufman JS. The impact of anti-tobacco legislation on birth weight in Peru. Glob Health Res Policy. 2020;5:5. doi:10.1186/s41256-020-00136-5 32161814 PMC7048150

[144] Markowitz S. The effectiveness of cigarette regulations in reducing cases of sudden infant death syndrome. J Health Econ. 2008;27(1):106-133. doi:10.1016/j.jhealeco.2007.03.006 17498829

[145] Markowitz S, Adams EK, Dietz PM, Tong VT, Kannan V. Tobacco control policies, birth outcomes, and maternal human capital. J Hum Cap. 2013;7(2):130-160. doi:10.1086/671020

[146] Mayne SL, Jacobs DR Jr, Schreiner PJ, Widome R, Gordon-Larsen P, Kershaw KN. Associations of smoke-free policies in restaurants, bars, and workplaces with blood pressure changes in the CARDIA study. J Am Heart Assoc. 2018;7(23):e009829. doi:10.1161/JAHA.118.009829 30571595 PMC6405556

[147] McGhee SM, Wong CM, Schooling CM, . Smoke-free policies on population health outcomes. Hong Kong Med J. 2014;20(3)(suppl 3):36-41. 25001035

[148] Menzies D, Nair A, Williamson PA, . Respiratory symptoms, pulmonary function, and markers of inflammation among bar workers before and after a legislative ban on smoking in public places. JAMA. 2006;296(14):1742-1748. doi:10.1001/jama.296.14.1742 17032987

[149] Naiman A, Glazier RH, Moineddin R. Association of anti-smoking legislation with rates of hospital admission for cardiovascular and respiratory conditions. CMAJ. 2010;182(8):761-767. doi:10.1503/cmaj.091130 20385737 PMC2871198

[150] Ozierański K, Witkowska A, Wojtyniak B, . Smoking ban in public places and myocardial infarction hospitalizations in a European country with high cardiovascular risk: insights from the Polish nationwide AMI-PL database. Pol Arch Intern Med. 2019;129(6):386-391. doi:10.20452/pamw.14862 31169258

[151] Patrick SW, Warner KE, Pordes E, Davis MM. Cigarette tax increase and infant mortality. Pediatrics. 2016;137(1):e20152901. doi:10.1542/peds.2015-2901 26628730 PMC4702024

[152] Peelen MJ, Sheikh A, Kok M, . Tobacco control policies and perinatal health: a national quasi-experimental study. Sci Rep. 2016;6:23907. doi:10.1038/srep23907 27103591 PMC4840332

[153] Pell JP, Haw S, Cobbe S, . Smoke-free legislation and hospitalizations for acute coronary syndrome. N Engl J Med. 2008;359(5):482-491. doi:10.1056/NEJMsa0706740 18669427

[154] Rajkumar S, Schmidt-Trucksäss A, Wellenius GA, . The effect of workplace smoking bans on heart rate variability and pulse wave velocity of non-smoking hospitality workers. Int J Public Health. 2014;59(4):577-585. doi:10.1007/s00038-014-0545-y 24504155 PMC4179883

[155] Rodu B, Peiper N, Cole P. Acute myocardial infarction mortality before and after state-wide smoking bans. J Community Health. 2012;37(2):468-472. doi:10.1007/s10900-011-9464-5 21877107

[156] Sargent JD, Demidenko E, Malenka DJ, Li Z, Gohlke H, Hanewinkel R. Smoking restrictions and hospitalization for acute coronary events in Germany. Clin Res Cardiol. 2012;101(3):227-235. doi:10.1007/s00392-011-0385-1 22350716 PMC3719165

[157] Sargent RP, Shepard RM, Glantz SA. Reduced incidence of admissions for myocardial infarction associated with public smoking ban: before and after study. BMJ. 2004;328(7446):977-980. doi:10.1136/bmj.38055.715683.55 15066887 PMC404491

[158] Schoj V, Alderete M, Ruiz E, Hasdeu S, Linetzky B, Ferrante D. The impact of a 100% smoke-free law on the health of hospitality workers from the city of Neuquén, Argentina. Tob Control. 2010;19(2):134-137. doi:10.1136/tc.2009.032862 20378587 PMC2989166

[159] Sen A, Piérard E. Estimating the effects of cigarette taxes on birth outcomes. Can Public Policy. 2011;37(2):257-276. doi:10.3138/cpp.37.2.257 22073425

[160] Seo DC, Torabi MR. Reduced admissions for acute myocardial infarction associated with a public smoking ban: matched controlled study. J Drug Educ. 2007;37(3):217-226. doi:10.2190/DE.37.3.a 18047180

[161] Shelley D, Yerneni R, Hung D, Das D, Fahs M. The relative effect of household and workplace smoking restriction on health status among Chinese Americans living in New York City. J Urban Health. 2007;84(3):360-371. doi:10.1007/s11524-007-9190-6 17410472 PMC2231828

[162] Shetty KD, DeLeire T, White C, Bhattacharya J. Changes in U.S. hospitalization and mortality rates following smoking bans. J Policy Anal Manage. 2010;30(1):6-28. doi:10.1002/pam.20548 21465828

[163] Simón L, Pastor-Barriuso R, Boldo E, . Smoke-free legislation in Spain and prematurity. Pediatrics. 2017;139(6):e20162068. doi:10.1542/peds.2016-2068 28562257

[164] Sims M, Maxwell R, Bauld L, Gilmore A. Short term impact of smoke-free legislation in England: retrospective analysis of hospital admissions for myocardial infarction. BMJ. 2010;340:c2161. doi:10.1136/bmj.c2161 20530563 PMC2882555

[165] Thach TQ, McGhee SM, So JC, . The smoke-free legislation in Hong Kong: its impact on mortality. Tob Control. 2016;25(6):685-691. doi:10.1136/tobaccocontrol-2015-052496 26585706

[166] Trachsel LD, Kuhn MU, Reinhart WH, Schulzki T, Bonetti PO. Reduced incidence of acute myocardial infarction in the first year after implementation of a public smoking ban in Graubuenden, Switzerland. Swiss Med Wkly. 2010;140(9-10):133-138. 20069475 10.4414/smw.2010.12955

[167] Di Valentino M, Muzzarelli S, Limoni C, . Reduction of ST-elevation myocardial infarction in Canton Ticino (Switzerland) after smoking bans in enclosed public places—No Smoke Pub Study. Eur J Public Health. 2015;25(2):195-199. doi:10.1093/eurpub/cku067 24895081

[168] Vander Weg MW, Rosenthal GE, Vaughan Sarrazin M. Smoking bans linked to lower hospitalizations for heart attacks and lung disease among medicare beneficiaries. Health Aff (Millwood). 2012;31(12):2699-2707. doi:10.1377/hlthaff.2011.0385 23213154

[169] Vartiainen E. The North Karelia Project: cardiovascular disease prevention in Finland. Glob Cardiol Sci Pract. 2018;2018(2):13. doi:10.21542/gcsp.2018.13 30083543 PMC6062761

[170] Vicedo-Cabrera AM, Röösli M, Radovanovic D, . Cardiorespiratory hospitalisation and mortality reductions after smoking bans in Switzerland. Swiss Med Wkly. 2016;146:w14381. doi:10.4414/smw.2016.14381 28102874

[171] Vicedo-Cabrera AM, Schindler C, Radovanovic D, . Benefits of smoking bans on preterm and early-term births: a natural experimental design in Switzerland. Tob Control. 2016;25(e2):e135-e141. doi:10.1136/tobaccocontrol-2015-052739 27118814

[172] Weaver AM, Wang Y, Rupp K, Watson DP. Effects of smoke-free air law on acute myocardial infarction hospitalization in Indianapolis and Marion County, Indiana. BMC Public Health. 2018;18(1):232. doi:10.1186/s12889-018-5153-y 29426315 PMC5810184

[173] Wilson T, Shamo F, Boynton K, Kiley J. The impact of Michigan’s Dr Ron Davis smoke-free air law on levels of cotinine, tobacco-specific lung carcinogen and severity of self-reported respiratory symptoms among non-smoking bar employees. Tob Control. 2012;21(6):593-595. doi:10.1136/tobaccocontrol-2011-050328 22705599

[174] Wu Y, Wang Z, Zheng Y, . The impact of comprehensive tobacco control policies on cardiovascular diseases in Beijing, China. Addiction. 2021;116(8):2175-2184. doi:10.1111/add.15406 33404152

[175] Yan J. The effects of a minimum cigarette purchase age of 21 on prenatal smoking and infant health. East Econ J. 2014;40(3):289-308. doi:10.1057/eej.2013.42

[176] Yıldız F, Barış SA, Başyiğit I, Boyacı H, Aydınlık H, Sönmez PÖ. Role of smoke-free legislation on emergency department admissions for smoking-related diseases in Kocaeli, Turkey. East Mediterr Health J. 2015;20(12):774-780. doi:10.26719/2014.20.12.774 25664515

[177] Flor LS, Reitsma MB, Gupta V, Ng M, Gakidou E. The effects of tobacco control policies on global smoking prevalence. Nat Med. 2021;27(2):239-243. doi:10.1038/s41591-020-01210-8 33479500 PMC7884287

[178] Levy DT, Tam J, Kuo C, Fong GT, Chaloupka F. The impact of implementing tobacco control policies: the 2017 Tobacco Control Policy Scorecard. J Public Health Manag Pract. 2018;24(5):448-457. doi:10.1097/PHH.0000000000000780 29346189 PMC6050159

[179] Pechacek TF, Babb S. How acute and reversible are the cardiovascular risks of secondhand smoke? BMJ. 2004;328(7446):980-983. doi:10.1136/bmj.328.7446.980 15105323 PMC404492

[180] Otsuka R, Watanabe H, Hirata K, . Acute effects of passive smoking on the coronary circulation in healthy young adults. JAMA. 2001;286(4):436-441. doi:10.1001/jama.286.4.436 11466122

[181] Eisner MD, Iribarren C, Yelin EH, . The impact of SHS exposure on health status and exacerbations among patients with COPD. Int J Chron Obstruct Pulmon Dis. 2009;4:169-176. doi:10.2147/COPD.S4681 19516915 PMC2685143

[182] Eisner MD, Balmes J, Yelin EH, . Directly measured secondhand smoke exposure and COPD health outcomes. BMC Pulm Med. 2006;6:12. doi:10.1186/1471-2466-6-12 16756671 PMC1524811

[183] Faber T, Been JV, Reiss IK, Mackenbach JP, Sheikh A. Smoke-free legislation and child health. NPJ Prim Care Respir Med. 2016;26:16067. doi:10.1038/npjpcrm.2016.67 27853176 PMC5113157

[184] Goodwin RD. Impact of cannabis use on nicotine and tobacco use outcomes. Nicotine Tob Res. 2020;22(8):1257-1259. doi:10.1093/ntr/ntaa096 32480403 PMC7364827

