# Supplementary material for: Evaluation of Population-Level Tobacco Control Interventions and Health Outcomes: A Systematic Review and Meta-Analysis
Source: JAMA Netw Open. 2023 Jul 7;6(7):e2322341. doi: 10.1001/jamanetworkopen.2023.22341 (PMC10329215; doi:10.1001/jamanetworkopen.2023.22341)
Supplement: Supplement 1. — eTable 1. Synthesis Past Evidence eTable 2. PubMed Search Results eTable 3. EMBASE Search Results eTable 4. Web of Science Search Results eTable 5. CINAHL Search Results eTable 6. EconLit Search Results eAppendix 1. Data Extraction Form eAppendix 2. Study Quality Assessment eFigure 1. Study Selection Flowchart eTable 7. Study Characteristics eFigure 2. Forest Plot of Meta-analysis of the Studies Examining the Associations of Smoke-free Legislation With Cardiovascular Disease (CVD), CVD Hospitalization, Respiratory Disease Hospitalization, CVD Mortality, Cancer, Respiratory Symptoms, Birth Weight, and Gestational Age Based on Coefficient Results eFigure 3. Meta-analysis of the Association of Smoke-free Legislation With Perinatal Mortality and Adverse Birth Outcomes eFigure 4. Forest Plot of Meta-analysis of Studies Examining the Association of Tax or Price Increase With Hospitalization Due to Cardiovascular Disease or Respiratory Symptoms eFigure 5. Forest Plot of Meta-analysis of the Studies Examining the Association of Tax or Price Increase With Total Mortality, Cardiovascular Disease Mortality, Cancer Mortality, Respiratory Disease Mortality, Cardiovascular Disease Hospitalization, and Respiratory Disease Hospitalization Based on Coefficient Results eFigure 6. Forest Plot of Meta-analysis of Studies Examining the Synergistic Associations of Tobacco Control Program (Combination of Several Tobacco Control Laws) With Cardiovascular Disease Hospitalization, Cardiovascular Disease Mortality, and Cancer Based on Coefficient Results eAppendix 3. Sensitivity Analyses eFigure 7. Publication Bias Assessment for Cardiovascular Diseases eFigure 8. Publication Bias Assessment for Smoke-free Legislation and Respiratory Disease and Symptoms eFigure 9. Publication Bias Assessment for Smoke-free Legislation and Perinatal Mortality and Adverse Birth Outcomes eTable 8. Summary, Publication Bias, and Trim-and-Fill Estimates eAppendix 4. Stratified Analysis eTable 9. Stratified Analys [file jamanetwopen-e2322341-s001.pdf]

Supplemental Online Content

Akter S, Islam R, Rahman M, et al. Evaluation of population-level tobacco control interventions and health outcomes: a systematic review and meta-analysis. *JAMA Netw Open*. 2023;6(7):e2322341. doi:10.1001/jamanetworkopen.2023.22341

eTable 1. Synthesis Past Evidence
eTable 2. PubMed Search Results
eTable 3. EMBASE Search Results
eTable 4. Web of Science Search Results
eTable 5. CINAHL Search Results
eTable 6. EconLit Search Results
eAppendix 1. Data Extraction Form
eAppendix 2. Study Quality Assessment
eFigure 1. Study Selection Flowchart
eTable 7. Study Characteristics
eFigure 2. Forest Plot of Meta-analysis of the Studies Examining the Associations of Smoke-free Legislation With Cardiovascular Disease (CVD), CVD Hospitalization, Respiratory Disease Hospitalization, CVD Mortality, Cancer, Respiratory Symptoms, Birth Weight, and Gestational Age Based on Coefficient Results
eFigure 3. Meta-analysis of the Association of Smoke-free Legislation With Perinatal Mortality and Adverse Birth Outcomes
eFigure 4. Forest Plot of Meta-analysis of Studies Examining the Association of Tax or Price Increase With Hospitalization Due to Cardiovascular Disease or Respiratory Symptoms
eFigure 5. Forest Plot of Meta-analysis of the Studies Examining the Association of Tax or Price Increase With Total Mortality, Cardiovascular Disease Mortality, Cancer Mortality, Respiratory Disease Mortality, Cardiovascular Disease Hospitalization, and Respiratory Disease Hospitalization Based on Coefficient Results
eFigure 6. Forest Plot of Meta-analysis of Studies Examining the Synergistic Associations of Tobacco Control Program (Combination of Several Tobacco Control Laws) With Cardiovascular Disease Hospitalization, Cardiovascular Disease Mortality, and Cancer Based on Coefficient Results
eAppendix 3. Sensitivity Analyses
eFigure 7. Publication Bias Assessment for Cardiovascular Diseases
eFigure 8. Publication Bias Assessment for Smoke-free Legislation and Respiratory Disease and Symptoms
eFigure 9. Publication Bias Assessment for Smoke-free Legislation and Perinatal Mortality and Adverse Birth Outcomes
eTable 8. Summary, Publication Bias, and Trim-and-Fill Estimates
eAppendix 4. Stratified Analysis
eTable 9. Stratified Analysis According to Study Design, Country Income Category and Quality Score
eTable 10. Stratified Analysis According to Comprehensiveness of Smoke-free Legislation
eTable 11. Narrative Summary of Adverse Health Outcomes Following the Implementation of the Smoking Ban
eTable 12. Narrative Summary of Adverse Health Outcomes Following the Implementation of Tobacco Law
eTable 13. Narrative Summary of Adverse Health Outcomes Following the Implementation of an Increase in Cigarette Prices or Cigarette Taxes
eTable 14. Narrative Summary of Adverse Health Outcomes Following the Implementation of Miscellaneous Policies
eReferences.

This supplemental material has been provided by the authors to give readers additional information about their work.

© 2023 Akter S et al. *JAMA Network Open.*

eTable 1. Synthesis Past Evidence

Study	# studies	Participant	Policy	Impact
Been et al. (2014)	11 (SR & MA)	Children	SL	Preterm birth (↓), LBW (×), VLBW (×), SGA (×), very SGA (↓), asthma admissions (↓)
Dinno & Glantz (2007)	4 (MA)	All	SL	Coronary heart disease admissions (↓)
Faber et al. (2017)	41 (SR & MA)	Children	TCP	Stillbirth (×), gestational age (×), very preterm birth (↓), birthweight (↑), LBW (↓), VLBW (×), SGA (↓), very SGA (×), congenital anomalies (×), early neonatal mortality (×), infant mortality (×), wheezing/asthma (×), respiratory infections (×)
Frazer et al. (2016)	72 (SR)	All	SL	AMI (×), ACS (×), stroke (×), cerebral infarction (×), COPD (×), asthma (×) admissions, lung function (×), mortality rate (AMI, IHD, stroke, COPD) (×), LBW (×), VLBW (×), preterm birth (×), SGA (×)
Gao et al (2019)	11 (MA)	All	SL	AMI mortality (↓)
Jones et al (2014)	31 (SR & MA)	All	SL	ACE admissions (↓)
Lightwood & Glantz (2009)	9 (MA)	All	SL	AMI admissions (↓)
Lin et al. (2013)	18 (SR & MA)	All	SL	AMI admissions (↓)
Mackay et al. (2010)	17 (MA)	Adults	SL	ACE admissions (↓)
Meyers et al (2009)	10 (SR & MA)	All	SL	AMI admissions (↓)
Rando-Matos et al (2017)	50 (SR & MA)	All	SL	Respiratory symptoms (↓), sensory symptoms (↓)
Tan & Glantz (2012)	55 (MA)	All	SL	Coronary events (AMI, ACS, ACE, IHD) (↓), other heart diseases [angina, coronary heart disease, sudden cardiac death (not admission)] (↓), cerebrovascular accidents (stroke, transient ischemic attack) (↓), respiratory disease (COPD, asthma, lung infection, spontaneous pneumothorax) (↓) admissions

MA, meta-analysis; SR, systematic review.

SL, Smoke-free legislation; TCP, Tobacco control policies.

LBW, low birthweight; VLBW, very low birthweight; SGA, small for gestational age; AMI, acute myocardial infarction; ACS, acute coronary syndrome; ACE, acute coronary events; COPD, chronic obstructive pulmonary disease; IHD, ischemic heart disease.

↓ indicate decrease, ↑ increase, × no clear association

© 2023 Akter S et al. *JAMA Network Open.*

eTable 2. PubMed Search Results

Search	Query	12 Feb 2021	1 Mar 2022
#5	Search: #1 AND #2 AND #3 AND #4	2,817	4,392
#4	Search: "epidemiologic studies"[MeSH] OR “epidemiologic stud*” [tw] OR “case control stud*” [tw] OR “case-control stud*” [tw] OR "cross-sectional studies"[MeSH] OR “cross-sectional stud*” [tw] OR “cross sectional stud” [tw] OR "cohort studies"[MeSH] OR “cohort stud*” [tw] OR cohort analy* [tw] OR “follow up stud*” [tw] OR “longitudinal stud*” [tw] OR “retrospective stud*” [tw] OR “prospective stud*” [tw] OR “observational stud*” [tw] OR "interrupted time series analysis"[MeSH Terms] OR “interrupted time series” [tw] OR “correlational” [tw] OR “descriptive stud*” [tw] OR “controlled before and after” [tw] OR "before and after study" [tw] OR “quasi-experimental” [tw] OR “quasi experimental*” [tw]	30,34,528	3,340,737
#3	Search: polic* OR intervention [tw] OR lobb* OR influence [tw] OR public policy [tw] OR law [tw] OR strategy [tw] OR strategies [tw] OR initiatives [tw] OR regulation [tw] OR actions [tw] OR plan [tw] OR “action plan” [tw] OR effects [tw]	8,888,682	10,888,682
#2	Search: taxation [tw] OR tax [tw] OR taxes [tw] taxed [tw] taxing [tw] OR excise [tw] OR cost [tw] OR price [tw] OR prices [tw] OR pricing [tw] OR Price increase [tw] OR promotion [tw] OR discount [tw] OR campaigns [tw] OR campaign [tw] OR ban [tw] OR partial ban [tw] OR full ban [tw] OR advertising [tw] OR advertisement [tw] OR tobacco control law [tw] OR smoke free law [tw] OR mass media [tw] OR pictorial warning [tw] OR health warning [tw] OR pictorial health warning [tw] OR graphic health warning [tw] OR graphic warning [tw] OR legal age [tw]	249,818	843,589
#1	Search: smoking [MeSH] OR smoking [tw] OR tobacco [MeSH] OR tobacco [tw] OR “tobacco use” [tw] OR “tobacco use disorder” [MeSH] OR “tobacco use disorder” [tw] OR “tobacco dependence” [tw] OR “smoking dependence” [tw] OR cigarette [MeSH] OR cigarette [tw] OR nicotine [MeSH] OR nicotine dependence [tw]	376,433	397,924

© 2023 Akter S et al. *JAMA Network Open.*

eTable 3. EMBASE Search Results

No.	Query	12 Feb 2021
**#5**	#1 AND #2 AND #3 AND #4	134
**#4**	(('epidemiologic studies':ti,ab,kw OR 'epidemiologic study':ti,ab,kw OR 'case control study':ti,ab,kw OR 'case control studies':ti,ab,kw OR 'case-control study':ti,ab,kw OR 'cross-sectional studies':ti,ab,kw OR 'cross-sectional study':ti,ab,kw OR 'cross sectional stud':ti,ab,kw OR 'cohort studies':ti,ab,kw OR 'cohort analysis':ti,ab,kw OR 'cohort study':ti,ab,kw OR 'follow up study':ti,ab,kw OR 'longitudinal study':ti,ab,kw OR 'retrospective study':ti,ab,kw OR 'prospective study':ti,ab,kw OR 'observational study':ti,ab,kw OR 'interrupted time series analysis':ti,ab,kw OR 'interrupted time series':ti,ab,kw OR 'correlational':ti,ab,kw OR 'descriptive study':ti,ab,kw OR 'descriptive studies':ti,ab,kw OR 'controlled before':ti,ab,kw) AND after:ti,ab,kw OR before:ti,ab,kw) AND 'after study':ti,ab,kw OR 'quasi-experimental':ti,ab,kw OR 'quasi experimental':ti,ab,kw	22,095
**#3**	'policy':ti,ab,kw OR 'policies':ti,ab,kw OR 'intervention':ti,ab,kw OR 'lobbies':ti,ab,kw OR 'lobby':ti,ab,kw OR 'influence':ti,ab,kw OR 'public policy':ti,ab,kw OR 'law':ti,ab,kw OR 'strategy':ti,ab,kw OR 'strategies':ti,ab,kw OR 'initiatives':ti,ab,kw OR 'regulation':ti,ab,kw OR 'actions':ti,ab,kw OR 'plan':ti,ab,kw OR 'action plan':ti,ab,kw OR 'campaigns':ti,ab,kw OR 'campaign':ti,ab,kw	5,143,713
**#2**	'taxation':ti,ab,kw OR 'tax':ti,ab,kw OR 'taxes':ti,ab,kw OR 'taxed':ti,ab,kw OR 'taxing':ti,ab,kw OR 'excise':ti,ab,kw OR 'cost':ti,ab,kw OR 'price':ti,ab,kw OR 'prices':ti,ab,kw OR 'pricing':ti,ab,kw OR 'promotion':ti,ab,kw OR 'discount':ti,ab,kw OR 'campaigns':ti,ab,kw OR 'campaign':ti,ab,kw	844,341
**#1**	'smoking':ti,ab,kw OR 'tobacco':ti,ab,kw OR 'tobacco use':ti,ab,kw OR 'tobacco use disorder':ti,ab,kw OR 'tobacco dependence':ti,ab,kw OR 'smoking dependence':ti,ab,kw OR 'cigarette':ti,ab,kw OR 'nicotine':ti,ab,kw OR 'nicotine dependence':ti,ab,kw	454,265

© 2023 Akter S et al. *JAMA Network Open.*

eTable 4. Web of Science Search Results

Search	Query	12 Feb 2021	1 Mar 2022
#5	#1 AND #2 AND #3 AND #4	238	731
#4	TI= ("epidemiologic studies" OR “epidemiologic study” OR “case control study” OR “case control studies” OR “case-control study” OR "cross-sectional studies" OR “cross-sectional study” OR “cross sectional stud” OR "cohort studies" OR “cohort study” OR “cohort analysis” OR “cohort study” OR “follow up study” OR “longitudinal study” OR “retrospective study” OR “prospective study” OR “observational study” OR "interrupted time series analysis OR “interrupted time series” OR “correlational” OR “descriptive study” OR “descriptive studies” OR “controlled before and after” OR "before and after study" OR “quasi-experimental” OR “quasi experimental”) OR TS=("epidemiologic studies" OR “epidemiologic study” OR “case control study” OR “case control studies” OR “case-control study” OR "cross-sectional studies" OR “cross-sectional study” OR “cross sectional stud” OR "cohort studies" OR “cohort study” OR “cohort analysis” OR “cohort study” OR “follow up study” OR “longitudinal study” OR “retrospective study” OR “prospective study” OR “observational study” OR "interrupted time series analysis OR “interrupted time series” OR “correlational” OR “descriptive study” OR “descriptive studies” OR “controlled before and after” OR "before and after study" OR “quasi-experimental” OR “quasi experimental”)	332,885	455,709
#3	TI= (“policy” OR “policies” OR “intervention” OR “lobbies” OR “lobby” OR “influence” OR “public policy” OR “law” OR “strategy” OR “strategies” OR “initiatives” OR “regulation” OR “actions” OR “plan” OR “action plan” OR “campaigns” OR “campaign”) OR TS= (“policy” OR “policies” OR “intervention” OR “lobbies” OR “lobby” OR “influence” OR “public policy” OR “law” OR “strategy” OR “strategies” OR “initiatives” OR “regulation” OR “actions” OR “plan” OR “action plan” OR “campaigns” OR “campaign”)	8,386,130	14,864,078
#2	TI= (“taxation” OR “tax” OR “taxes” OR “taxed” OR “taxing” OR “excise” OR “cost” OR “price” OR “prices” OR “pricing” OR “promotion” OR “discount” OR “campaigns” OR “campaign” OR “ban” OR “partial ban” OR “full ban” OR “advertising” OR “advertisement” OR “tobacco control law” OR “smoke free law” OR “mass media” OR “pictorial warning” OR “health warning” OR “pictorial health warning” OR “graphic health warning” OR “graphic warning” OR “legal age”) OR TS= (“taxation” OR “tax” OR “taxes” OR “taxed” OR “taxing” OR “excise” OR “cost” OR “price” OR “prices” OR “pricing” OR “promotion” OR “discount” OR “campaigns” OR “campaign” OR “ban” OR “partial ban” OR “full ban” OR “advertising” OR “advertisement” OR “tobacco control law” OR “smoke free	1,953,848	7,848,532

© 2023 Akter S et al. *JAMA Network Open.*

	law" OR "mass media" OR "pictorial warning" OR "health warning" OR "pictorial health warning" OR "graphic health warning" OR "graphic warning" OR "legal age")		
#1	TI = ("smoking" OR "tobacco" OR "tobacco use" OR "tobacco use disorder" OR "tobacco dependence" OR "smoking dependence" OR "cigarette" OR "nicotine" OR "nicotine dependence") OR TS = ("smoking" OR "tobacco" OR "tobacco use" OR "tobacco use disorder" OR "tobacco dependence" OR "smoking dependence" OR "cigarette" OR "nicotine" OR "nicotine dependence")	406,906	1,078,961

eTable 5. CINAHL Search Results

Search	Query	12 Feb 2021	1 Mar 2022
#S5	#S1 AND #S2 AND #S3 AND #S4	111	145
#S4	TI ("epidemiologic studies" OR “epidemiologic study” OR “case control study” OR “case control studies” OR “case-control study” OR "cross-sectional studies" OR “cross-sectional study” OR “cross sectional stud” OR "cohort studies" OR “cohort study” OR “cohort analysis” OR “cohort study” OR “follow up study” OR “longitudinal study” OR “retrospective study” OR “prospective study” OR “observational study” OR "interrupted time series analysis OR “interrupted time series” OR “correlational” OR “descriptive study” OR “descriptive studies” OR “controlled before and after” OR "before and after study" OR “quasi-experimental” OR “quasi experimental”) OR AB ("epidemiologic studies" OR “epidemiologic study” OR “case control study” OR “case control studies” OR “case-control study” OR "cross-sectional studies" OR “cross-sectional study” OR “cross sectional stud” OR "cohort studies" OR “cohort study” OR “cohort analysis” OR “cohort study” OR “follow up study” OR “longitudinal study” OR “retrospective study” OR “prospective study” OR “observational study” OR "interrupted time series analysis OR “interrupted time series” OR “correlational” OR “descriptive study” OR “descriptive studies” OR “controlled before and after” OR "before and after study" OR “quasi-experimental” OR “quasi experimental”)	83,481	128,370
#S3	TI (“policy” OR “policies” OR “intervention” OR “lobbies” OR “lobby” OR “influence” OR “public policy” OR “law” OR “strategy” OR “strategies” OR “initiatives” OR “regulation” OR “actions” OR “plan” OR “action plan” OR “campaigns” OR “campaign”) OR AB (“policy” OR “policies” OR “intervention” OR “lobbies” OR “lobby” OR “influence” OR “public policy” OR “law” OR “strategy” OR “strategies” OR “initiatives” OR “regulation” OR “actions” OR “plan” OR “action plan” OR “campaigns” OR “campaign”)	110,649	1,020,980
#S2	TI (“taxation” OR “tax” OR “taxes” OR “taxed” OR “taxing” OR “excise” OR “cost” OR “price” OR “prices” OR “pricing” OR “promotion” OR “discount” OR “campaigns” OR “campaign” OR “ban” OR “partial ban” OR “full ban” OR “advertising” OR “advertisement” OR “tobacco control law” OR “smoke free law” OR “mass media” OR “pictorial warning” OR “health warning” OR “pictorial health warning” OR “graphic health warning” OR “graphic warning” OR “legal age”) OR AB (“taxation” OR “tax” OR “taxes” OR “taxed” OR “taxing” OR “excise” OR “cost” OR “price” OR “prices” OR “pricing” OR “promotion” OR “discount” OR “campaigns” OR “campaign OR “ban” OR “partial ban” OR “full ban” OR “advertising” OR “advertisement” OR “tobacco control law” OR “smoke free law”	950,868	214,516

© 2023 Akter S et al. *JAMA Network Open.*

	OR “mass media” OR “pictorial warning” OR “health warning” OR “pictorial health warning” OR “graphic health warning” OR “graphic warning” OR “legal age”)		
#S1	TI (“smoking” OR “tobacco” OR “tobacco use” OR “tobacco use disorder” OR “tobacco dependence” OR “smoking dependence” OR “cigarette” OR “nicotine” OR “nicotine dependence”) OR AB (“smoking” OR “tobacco” OR “tobacco use” OR “tobacco use disorder” OR “tobacco dependence” OR “smoking dependence” OR “cigarette” OR “nicotine” OR “nicotine dependence”)	204,259	108,308

© 2023 Akter S et al. *JAMA Network Open.*

eTable 6. EconLit Search Results

Search	Query	12 Feb 2021	1 Mar 2022
S4	#S1 AND #S2 AND #S3	175	209
S3	TI = (“policy” OR “policies” OR “intervention” OR “lobbies” OR “lobby” OR “influence” OR “public policy” OR “law” OR “strategy” OR “strategies” OR “initiatives” OR “regulation” OR “actions” OR “plan” OR “action plan” OR “campaigns” OR “campaign”) OR AB= (“policy” OR “policies” OR “intervention” OR “lobbies” OR “lobby” OR “influence” OR “public policy” OR “law” OR “strategy” OR “strategies” OR “initiatives” OR “regulation” OR “actions” OR “plan” OR “action plan” OR “campaigns” OR “campaign”)	175,254	182,988
S2	TI = (“taxation” OR “tax” OR “taxes” OR “taxed” OR “taxing” OR “excise” OR “cost” OR “price” OR “prices” OR “pricing” OR “promotion” OR “discount” OR “campaigns” OR “campaign” OR OR “ban” OR “partial ban” OR “full ban” OR “advertising” OR “advertisement” OR “tobacco control law” OR “smoke free law” OR “mass media” OR “pictorial warning” OR “health warning” OR “pictorial health warning” OR “graphic health warning” OR “graphic warning” OR “legal age”) OR AB = (“taxation” OR “tax” OR “taxes” OR “ ...	329,530	347,928
S1	T I= (“smoking” OR “tobacco” OR “tobacco use” OR “tobacco use disorder” OR “tobacco dependence” OR “smoking dependence” OR “cigarette” OR “nicotine” OR “nicotine dependence” OR) OR AB =(“smoking” OR “tobacco” OR “tobacco use” OR “tobacco use disorder” OR “tobacco dependence” OR “smoking dependence” OR “cigarette” OR “nicotine” OR “nicotine dependence”)	2,986	3,242

© 2023 Akter S et al. *JAMA Network Open.*

eAppendix 1. Data Extraction Form

Section 1: General and background information

Article ID	
Data extractor	
Article title	
Author names (Last name et al)	
Year of publication	
Country	
Place<br>*(National/Urban/Rural/others)*	
Region	
Study subjects<br>*(Adult/Elderly/children/All)*	
Gender<br>*(Both/Male/Female)*	
Age of the participants (Years)	
Mean or median age of participants	
Study design<br>*(Cross-section, cohort, case-control, longitudinal, control before and after, interrupted time series, others)*	
Survey start	
Survey end	
Sample size	
Sampling methods	
Please specify covariates adjusted in analysis	

© 2023 Akter S et al. *JAMA Network Open.*

Section 2: Please provide policy name /interventions with brief description or definition in the following tables.

No.	Name of policy	Implementation date	Description/Definition	Description of comparator
1				
2				
3				
4				
5				
6				
7				
8				

Section 3: Please provide outcomes *name* with brief description or definition in the following tables.

No.	Outcomes	Description/Definition
1		
2		
3		
4		
5		
6		
7		
8		

Section 4: Effect size by outcome and policy

Name of policy	Name of outcome	Name of effect size (OR/RR/HR/ coefficient/others)[a]	Please report values of OR/RR/coefficient	Please report SE, or 95% CI
Smoke-free legislation	Cardiovascular disease	Hazard ratio	0.58	0.33-0.99

Note: the effects size should be fill by policy and outcome. One example is provided for smoke-free legislation by outcome. Please use extra rows or drop rows if required

Name of effect size: odds ratio (OR), risk ratio (RR), hazard ratio (HR), coefficients, percentage change, mean difference, rate of changes.

© 2023 Akter S et al. *JAMA Network Open.*

Section 5: Please report briefly on each item in the following table

	Author reported
Objectives	
Participants selection	
Statistical methods	
Unit of analysis	
Results	
Conclusion	

© 2023 Akter S et al. *JAMA Network Open.*

eAppendix 2. Study Quality Assessment

Observational studies: We employed a specific checklist to assess the methodological quality of all included cohort studies using the Newcastle–Ottawa Scale (NOS) recommended by Wells and colleagues [1]. This system is based on a system of stars (*) awarded for each applicable criterion. Three major domain-based approaches were used for evaluating the observational study quality: selection, comparability, and outcome.

Cohort Study: The cohort studies used nine criteria: the representativeness of the exposed cohort, the selection of the non-exposed cohort, ascertainment of exposure, outcome of interest not present at start (maximum of four stars); comparability of the cohorts based on study design and analysis (maximum of two stars); and finally, the assessment of the outcome (maximum of three stars). All studies received a score based on these nine criteria, ranging from 0 to 9. Studies were defined as high quality, score ≥ 6; moderate quality, score 4-5; or low quality, score 0-3.

	**Selection**				**Comparability**		**Outcome**			
	1	2	3	4	5A	5B	6	7	8	
Study, year	Exposed cohort truly representative	Non-exposed cohort drawn from the same community	Ascertainment of exposure	Outcome of interest not present at start	Cohorts comparable on basis of age	Cohorts comparable on other factor(s)	Quality of outcome assessment	Follow-up long enough for outcomes to occur	Complete accounting for cohorts	Total Score

***Cross-sectional study**:* The cross-sectional studies used 10 criteria: the representativeness of the sample, sample size determination, non-response, ascertainment of exposure (maximum of five stars); comparability of the study on the basis of study design and analysis (maximum of two stars); and finally, the assessment of the outcome (maximum of three stars). All studies received a score on the basis of these nine criteria, ranging from 0 to 10. Studies were defined as high quality, score ≥ 6; moderate quality, score 4-5; or low quality, score 0-3.

	Selection (5 points)				Comparability (2 points)		Outcome (3 points)		Total Score
	1	2	3	4	5A	5B	6	7	
Study, year	Representativeness of the sample (*)	Sample size (*)	Non-respondents (*)	Ascertainment of the exposure (**)	Controls for age (*)	Control for any additional factor (*)	Assessment of the outcome (**)	Statistical test (*)	

© 2023 Akter S et al. *JAMA Network Open.*

Case-control study: The case-control studies used 9 criteria: definition of case, representativeness of the case, selection of controls, definition of controls (maximum 4 stars); controls for age, controls for any additional factors (maximum of two stars); ascertainment of exposure, ascertainment of case and controls (maximum of two stars). All studies received a score on the basis of these nine criteria, ranging from 0 to 10. Studies were defined as high quality, score ≥ 6; moderate quality, score 4-5; or low quality, score 0-3.

Study, year	**Selection (4 points)**				**Comparability (2 points)**		**Exposure (3 points)**		Total Score
	1	2	3	4	5A	5B	6	7	
	Definition of case (*)	Representativeness of the case (*)	Selection of controls (*)	Definition of controls (*)	Controls for age (*)	Control for any additional factor (*)	Ascertainment of exposure (*)	Ascertainment of case and controls (*)	

Experimental/quasi-experimental studies: Cochrane EPOC tools were used for assessing the risk of bias for quasi-experimental studies **such as c**ontrolled before and after, and interrupted time series [2]. Risk of bias was evaluated through the following items: confounding bias, detection bias (only in non-experimental studies- two domains), selection bias, attrition bias, reporting bias, other bias.
(1) Confounding bias: Comparability of groups for quasi-experimental studies. Such as, was the policy independent of other changes?
(2) Detection bias: Was the shape of the policy effect pre-specified? Was the policy unlikely to affect data collection?
(3) Selection bias: Sample representativeness.
(4) Attrition bias: Describe the completeness of outcome data for each main outcome, including attrition and exclusions from the analysis. - Reporting bias: State how the possibility of selective outcome reporting was examined by the review authors, and what was found.
(5) Other bias: State any important concerns about bias not addressed in the other domains in the tool.

**Study, year**	**Confounding bias**	**Detection Bias1**	**Detection Bias2**	**Selection Bias**	**Attrition Bias**	**Reporting Bias**	**Other Bias**	**Total Score**

We rated the overall methodological quality of the included studies as being at low, moderate, or high risk of bias. For non-experimental studies without control group, seven domains of bias were used. Articles with 5 or more subcategories of low bias are classified as "Low" in the summary risk of bias; articles with low bias subcategories between 3 and 4 are classified as "Moderate"; and those with subcategories between 1 and 2 classified as "High". For quasi-experimental studies with control group, five domains of bias were used. Articles with 4 or more subcategories with low bias are classified as "Low" in the summary risk of bias; the ones with 3 subcategories classified as "Moderate"; and thos with subcategories between 1 and 2 classified as "High".

For comparison with observational studies and to make a unique score, we change subcategory name from "High risk of bias" to "low quality study"; "Moderate risk of bias" to "moderate quality study", and "Low risk of bias" to "high-quality study". **NB:** Two reviewers independently assessed the study quality, which was then cross-checked by two other authors. Disagreements were resolved through discussion.

© 2023 Akter S et al. *JAMA Network Open.*

eFigure 1. Study Selection Flowchart

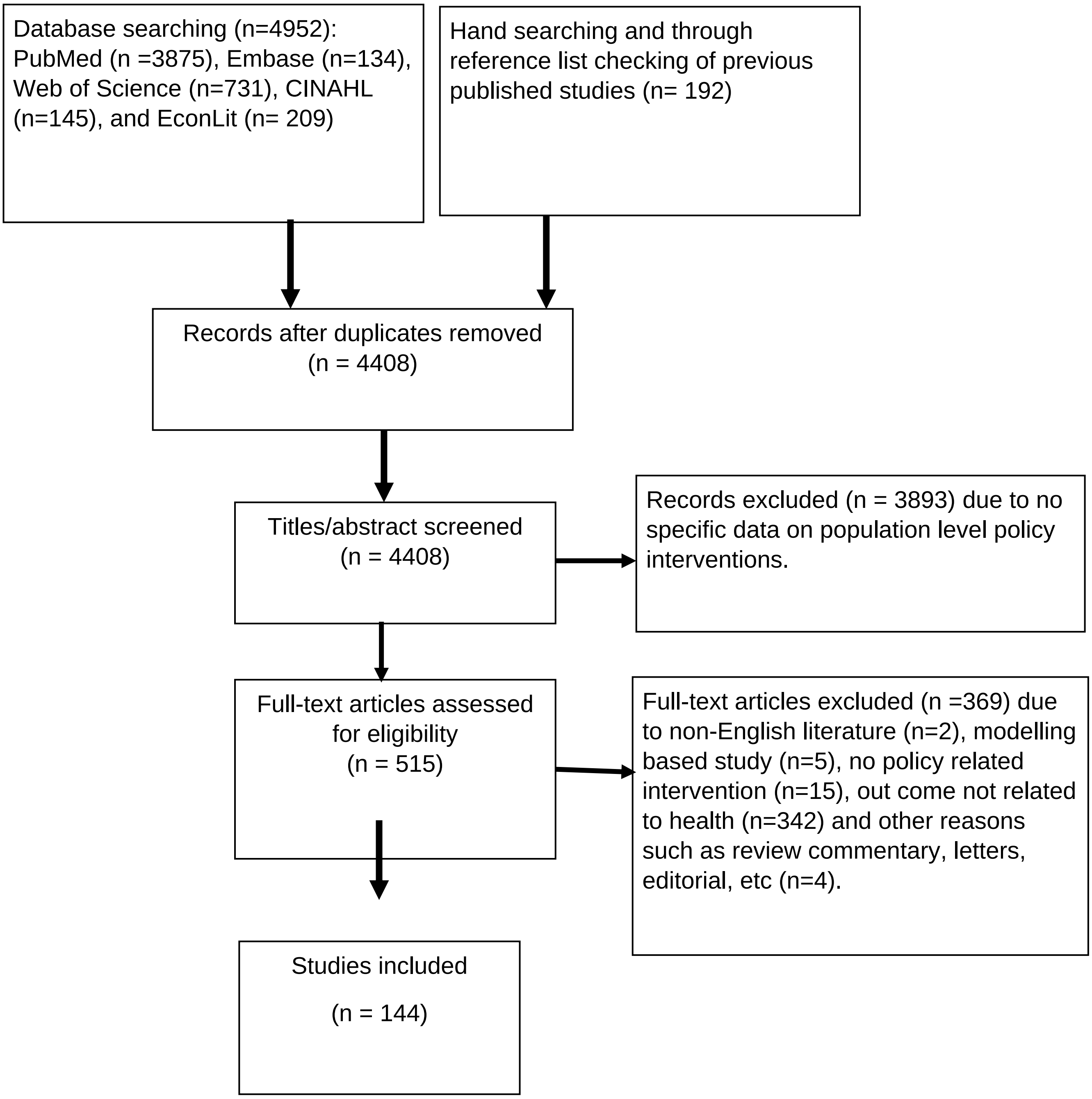

© 2023 Akter S et al. *JAMA Network Open.*

eTable 7. Study Characteristics

Study (publication year)	Study design (location)[1]	Participant (age, years)[2]	Policy start date	Name of policy[3]	Outcome[4]	Study quality
Abe et al. (2016)[3]	ITS (Brazil)	NA (NA)	2009	Smoke-free legislation	AMI ─ mortality rate, hospitalization rate	Moderate
Abreu et al. (2017)[4]	ITS (Portugal)	All (≥0)	2008	Smoke-free legislation	Acute coronary syndrome ─ hospitalization rate	Moderate
Adams et al. (2013)[5]	Cross-sectional(USA)	Female (<20 - ≥ 40)	1998	Smoking cessation service (enrollment)	Birth outcomes	Moderate
Aguero et al. (2013)[6]	ITS (Spain)	All (25-74)	2006	Smoke-free legislation	AMI ─ case fatality rate, mortality rate, incidence rate	Moderate
Allwright et al. (2005)[7]	CBA (Ireland)	All (20.9-54.9)	2004	Smoke-free legislation	Respiratory symptoms, sensory symptoms ─ prevalence	High
Alsever et al. (2009)[8]	CBA (USA)	All (NA)	2003	Smoke-free legislation	AMI ─ hospitalization rate	High
Amaral (2009)[9]	Cross-sectional (USA)	All (39.55 gestational weeks)	1995	Smoke-free legislation	Birth outcomes	High
Ayres et al. (2009)[10]	Longitudinal (Scotland)	All (≥15)	2006	Smoke-free legislation	Respiratory symptoms, sensory symptoms ─ prevalence	Moderate
Bakolis et al. (2016)[11]	ITS (UK)	All (24-44 gestational weeks)	2007	Smoke-free legislation	Birth outcomes	Moderate
Bannon et al. (2009)[12]	Cross-sectional (Ireland)	All (≥16)	2007	Smoke-free legislation	Respiratory symptoms, sensory symptoms ─ prevalence	Moderate
Barnett et al. (2009)[13]	ITS (New Zealand)	All (≥30)	2003	Smoke-free legislation	AMI ─ hospitalization rate	Moderate
Barnoya & Glantz. (2004)[14]	ITS (USA)	NA (NA)	1998	Tobacco control program	Lung cancer, bladder cancer ─ incidence rate	Moderate
Barone-Adesi et al. (2006)[15]	ITS (Italy)	All (≥0)	2005	Smoke-free legislation	Acute coronary events, AMI ─ hospitalization rate	Moderate
Barone-Adesi et al. (2011)[16]	ITS (Italy)	All (≥0)	2005	Smoke-free legislation	Ischemic heart disease ─ hospitalization rate	Moderate
Barr et al. (2012)[17]	Cohort (USA)	All (≥ 65)	1999	Smoke-free legislation	AMI ─ hospitalization rate	High
Barrio et al. (2019)[18]	Longitudinal (Spain)	All (≥25)	2006	Smoke-free legislation	Coronary heart disease, respiratory disease ─ mortality rate	High
Bartecchi et al. (2006)[19]	CBA (USA)	All (50.6-83.1)	2003	Smoke-free legislation	AMI ─ hospitalization rate	High

© 2023 Akter S et al. *JAMA Network Open.*

Bartholomew & Abouk (2016)[20]	Cross-sectional (USA)	Female (<18 - 21+); Both (38.78 gestational weeks)	1992	Smoke-free legislation	Birth outcomes	High
Basel et al. (2014)[21]	CBA (USA)	All (52.5-81.3)	2006	Smoke-free legislation	AMI ─ hospitalization rate	Low
Been et al. (a) (2015)[22]	ITS (UK)	All (0-14)	2007	Smoke-free legislation	Acute respiratory tract infections ─ hospitalization rate	High
Been et al. (b) (2015)[23]	ITS (UK)	All (0-12)	2006-2007	Smoke-free legislation	Respiratory disease ─ incidence rate	High
Been et al. (c) (2015)[24]	ITS (UK)	All (≤1)	2007	Smoke-free legislation	Early life outcomes	Moderate
Bharadwaj et al. (2014)[25]	Cross-sectional (Norway)	All (8-12 gestational weeks)	2004	Smoke-free legislation	Birth outcomes	High
Bianchi et al. (2011)[26]	Cross-sectional (Italy)	All (0-14)	2005	Smoke-free legislation	Asthma ─ hospitalization rate	Low
Bonetti et al. (2011)[27]	CBA (Switzerland)	NA (NA)	2008	Smoke-free legislation	AMI ─ hospitalization rate	Moderate
Bowser et al. (2016)[28]	Longitudinal (USA)	All (≥0)	1970-2005	Tobacco tax	Mortality rate	Moderate
Bruintjes et al. (2011)[29]	Cross-sectional (USA)	All (65-69)	2003	Smoke-free legislation	AMI ─ hospitalization rate	High
Carrión-Valero et al. (2020)[30]	Cross-sectional (Spain)	All (≥40)	2005, 2010	Smoke-free legislation	AMI ─ hospitalization rate	High
Carrión-Valero et al. (2020)[31]	Cohort (Spain)	All (≥20)	2005, 2010	Smoke-free legislation	Cardiovascular, respiratory, cancer disease ─ hospitalization rate	Low
Cesaroni et al. (2008)[32]	ITS (Italy)	All (35-84)	2005	Smoke-free legislation	Acute coronary events ─ hospitalization, mortality rate	Moderate
Ciaccio et al. (2016)[33]	ITS (USA)	All (<18)	2003	Smoke-free legislation	Asthma ─ emergency visits rate	High
Cox et al. (2014)[34]	ITS (Belgium)	All (≥30)	2006, 2007	Smoke-free legislation	AMI ─ mortality rate	Moderate
Croghan et al. (2015)[35]	Cohort (USA)	All (≥0)	2007	Smoke-free legislation	COPD, asthma ─ emergency visits rate	High
Cronin et al. (2012)[36]	Longitudinal (Ireland)	All (≥18)	2004	Smoke-free legislation	Acute coronary syndromes ─ hospitalization rate	Moderate
de Korte-de Boer et al. (2012)[37]	ITS (Netherlands)	All (20-75)	2004	Smoke-free legislation	Sudden circulatory arrest ─ incidence rate	Moderate

© 2023 Akter S et al. *JAMA Network Open.*

Dilley et al. (2012)[38]	ITS (USA)	NA (NA)	2000, 2005	Tobacco control program	Ischemic heart disease, cerebrovascular disease, chronic respiratory disease, esophageal cancer, larynx cancer, oral cancer, lung cancer ─ incidence rate	Low
Di Valentino et al. (2014)[39]	CBA (Switzerland)	All (≥0)	2007	Smoke-free legislation	ST-elevation myocardial infarction (STEMI) ─ hospitalization rate	High
Dove et al. (2010)[40]	Cross-sectional (USA)	All (≥35)	2004	Smoke-free legislation	AMI ─ mortality rate	High
Dove et al. (2011) [41]	ITS (USA)	All (3-15)	NA	Smoke-free legislation	Asthma ─ prevalence	Low
Durham et al. (2011)[42]	Cohort (Switzerland)	NA (NA)	2009	Smoke-free legislation	Respiratory symptoms ─ prevalence	Low
Eagan et al. (2006)[43]	Cohort (Norway)	All (≥15)	2004	Smoke-free legislation	Respiratory symptoms ─ prevalence	Low
Eisner et al. (1998)[44]	Cohort (USA)	NA (NA)	1998	Smoke-free legislation	Respiratory symptoms ─ prevalence	Moderate
Evans & Ringel (1999)[45]	Cross-sectional (USA)	Female (15-44)	1982	Tobacco tax	Birth outcomes	High
Farrelly (2005)[46]	Cohort (USA)	All (≥18)	2003	Smoke-free legislation	Respiratory symptoms, sensory symptoms ─ prevalence	Moderate
Fernandez (2009)[47]	Cohort (Spain)	Female (30.9-48.8)	2006	Smoke-free legislation	Respiratory symptoms ─ prevalence	High
Ferrante et al. (2011)[48]	ITS (Argentina)	NA (NA)	2006	Smoke-free legislation	Acute coronary syndrome, AMI ─ hospitalization rate	Low
Fichtenberg et al. (2000)[49]	Cross-sectional (USA)	All (≥25)	1989	Tobacco control program	Heart disease ─ mortality rate	High
Galán et al. (2015)[50]	ITS (Spain)	All (≥18)	2006	Smoke-free legislation	AMI, cerebrovascular disease, COPD, asthma ─ hospitalization rate	High
Galán et al. (2017)[51]	ITS (Spain)	All (<15- ≥ 65)	2006	Smoke-free legislation	Chronic respiratory diseases ─ hospitalization rate	High
Galán et al. (2018)[52]	ITS (Spain)	All (≥18)	2006, 2011	Smoke-free legislation	AMI, ischemic heart disease, cerebrovascular disease ─ hospitalization rate	Low
Gao & Baughman (2017)[53]	ITS (USA)	Female (14-45)	1995	Smoke-free legislation	Birth outcomes	Moderate

© 2023 Akter S et al. *JAMA Network Open.*

Gasparrini et al. (2009)[54]	ITS (Italy)	All (30-64)	2005	Smoke-free legislation	AMI ─ incidence rate	High
Gaudreau et al. (2013)[55]	ITS (Canada)	All (0-104)	2003	Smoke-free legislation	AMI, angina, stroke, COPD, appendicitis, pancreatitis, bowel obstruction, asthma ─ hospitalization rate	High
Goodman et al. (2007)[56]	CBA (Ireland)	Male (6-52)	2004	Smoke-free legislation	Respiratory symptoms, sensory symptoms ─ prevalence	Low
Gupta et al. (2011)[57]	ITS (USA)	All (≥18)	2004	Smoke-free legislation	Acute coronary syndrome ─ hospitalization rate	High
Hahn et al. (2011)[58]	ITS (USA)	All (≥35)	2004	Smoke-free legislation	AMI ─ hospitalization rate	High
Hajdu & Hajdu (2018)[59]	Cross-sectional (Hungary)	Female (NA)	2012	Smoke-free legislation	Pregnancy outcomes ─ prevalence	High
Hankins & Tarasenko (2016)[60]	ITS (USA)	Female (NA)	1991-2009	Smoke-free legislation	Neonatal outcomes ─ prevalence	High
Hatoun et al. (2018)[61]	Cross-sectional (USA)	All (6-19)	2006	Tobacco tax	Asthma severity	High
Hawkins et al. (2014)[62]	ITS (USA)	Female (18-50)	2000-2010	Smoke-free legislation, Tobacco tax	Birth outcomes	High
Hawkins et al. (2016)[63]	ITS (USA)	All (0-17)	2006	Smoke-free legislation	Asthma, ear infections, respiratory infections ─ emergency visits rate	High
Hawkins & Baum et al. (2019) [64]	ITS (USA)	Female (16-49)	2005-2015	Smoke-free legislation, Tobacco tax	Birth outcomes	High
Hawkins & Baum et al. (2019)[65]	ITS (USA)	Female (16-49)	2005-2015	Smoke-free legislation, Tobacco tax	Birth defects	High
Head et al. (2012)[66]	CBA (USA)	All (NA)	2006	Smoke-free legislation	AMI, stroke or cerebrovascular accident, transient ischemic attack, COPD, asthma ─ discharge rate	High
Herman & Walsh (2011)[67]	CBA (USA)	NA (NA)	2007	Smoke-free legislation	AMI, unstable angina, acute stroke, acute asthma ─ hospitalization rate	Moderate
Ho et al. (2017)[68]	CBA (USA)	All (≥18)	2001-2008	Smoke-free legislation, Tobacco tax	AMI, congestive heart failure, pneumonia ─ hospitalization rate	High

© 2023 Akter S et al. *JAMA Network Open.*

Holford et al. (2014)[69]	ITS (USA)	All (0-65)	1964	Tobacco control program	Mortality rate (smoking-attributable)	Moderate
Hone et al. (2020)[70]	CBA (Brazil)	All (0-1)	2014	Smoke-free legislation	Infant and Neonatal mortality rate	Moderate
Humair et al. (2014)[71]	Cross-sectional (Switzerland)	All (≥16)	2009	Smoke-free legislation	Acute COPD, pneumonia, acute asthma, acute coronary syndrome, ischemic stroke ─ hospitalization rate	High
Hurt et al. (2012)[72]	ITS (USA)	All (42-82)	2007	Smoke-free legislation	Myocardial infarction, sudden cardiac death ─ incidence rate	High
Jan et al. (2014)[73]	ITS (Panama)	All (30-98)	2008	Smoke-free legislation, Tobacco tax	AMI ─ hospitalization rate	High
Jarlenski et al. (2014)[74]	Cohort (USA)	Female (19-44)	2004-2010	Smoking cessation service (enrollment)	Birth outcomes	Low
Jiang et al. (2019)[75]	ITS (Australia)	All (≥15)	1960-1987	Reports/Media campaign on dangers of tobacco, Cigarette-, Tobacco-advertisement ban	All cancers, lung cancer ─ mortality rate	High
Johnson & Beal (2012)[76]	Cross-sectional (USA)	All (28-70)	2010	Smoke-free legislation	Heart attack ─ hospitalization rate, mortality rate	High
Juster et al. (2007)[77]	ITS (USA)	All (≥ 35)	2003	Smoke-free legislation	AMI ─ hospitalization rate	High
Kabir et al. (2009)[78]	Cross-sectional (Ireland)	Female (<20 - >34)	2004	Smoke-free legislation	Birth outcomes	High
Kabir et al. (2013)[79]	Cross-sectional (Ireland)	All (24-41 gestational weeks)	2004	Smoke-free legislation	Birth outcomes	High
Kalkhoran et al. (2014)[80]	ITS (Uruguay)	All (≥15)	2006	Smoke-free legislation	Bronchospasm ─ hospitalization rate, bronchospasm ─ emergency visits rate	High
Kent et al. (2012)[81]	Cross-sectional (Ireland)	All (20-70)	2004	Smoke-free legislation	Pulmonary disease, COPD, asthma, pneumonia, lower respiratory tract infection, spontaneous pneumothorax, acute coronary syndrome, myocardial infarction, unstable angina, cerebrovascular syndromes, stroke/cerebrovascular	High

© 2023 Akter S et al. *JAMA Network Open.*

					accident, transient ischemic attack ─ hospitalization rate	
Khuder et al. (2007)[82]	CBA (USA)	All (≥18)	2002	Smoke-free legislation	Coronary heart disease ─ hospitalization rate	High
Kim et al. (2015)[83]	Longitudinal (Korea)	All (35-50)	2013	Smoke-free legislation	Respiratory symptoms, sensory symptoms ─ prevalence	Moderate
Kong et al. (2021)[84]	Cross-sectional (USA)	Male (≥45)	NA	Tobacco retailer density	COPD ─ discharge rate	High
Landers (2014)[85]	CBA (USA)	All (NA)	2003-2008	Smoke-free legislation	Asthma ─ discharge rate	Moderate
Larsson et al. (2008)[86]	Longitudinal (Sweden)	All (18-65)	2005	Smoke-free legislation	Respiratory symptoms ─ prevalence	High
Lee et al. (2016)[87]	CBA (China)	All (≤18)	2007	Smoke-free legislation	Lower respiratory tract infection ─ hospitalization rate	High
Lemstra et al. (2008)[88]	CBA (Canada)	All (65-72)	2004	Smoke-free legislation	AMI ─ incidence rate	High
Li e t al. (2013)[89]	Longitudinal (China)	All (16-65)	2010	Smoke-free legislation	Respiratory symptoms, sensory symptoms ─ prevalence	Moderate
Lippert & Gustat (2012)[90]	Cross-sectional (USA)	All (≥18)	2008	Smoke-free legislation	Coronary heart disease, angina, AMI ─ prevalence	High
Liu et al. (2013)[91]	ITS (UK)	All (≥18)	2007	Smoke-free legislation	Coronary heart disease, myocardial infarction ─ hospitalization rate	High
Loomis et al. (2012)[92]	ITS (USA)	All (≥35)	2003	Smoke-free legislation	AMI, stroke ─ hospitalization rate	High
Ma ZQ et al. (2013)[93]	ITS (USA)	All (18 - ≥40)	2002-2004	Tobacco tax	AMI, asthma ─ hospitalization rate sudden cardiac death ─ mortality rate	High
MacCalman et al. (2012)[94]	Longitudinal (England and Scotland)	All (≥18)	2006, 2007	Smoke-free legislation	Respiratory symptoms, sensory symptoms ─ prevalence	Moderate
Mackay et al. (2010)[95]	Cross-sectional (Scotland)	All (< 15)	2006	Smoke-free legislation	Asthma ─ hospitalization rate	High
Mackay et al. (2012)[96]	Cohort (Scotland)	All (24-44 gestational weeks)	2006	Smoke-free legislation	Pregnancy complications ─ prevalence	High
Mackay et al. (2013)[97]	ITS (Scotland)	All (≥18)	2006	Smoke-free legislation	Cerebral infarction, stroke ─ incidence rate	High
Madureira et al. (2013)[98]	Longitudinal (Portugal)	All (≥18)	2008	Smoke-free legislation	Respiratory symptoms, sensory symptoms ─ prevalence	Moderate

© 2023 Akter S et al. *JAMA Network Open.*

Mallma et al. (2020)[99]	ITS (USA)	All (≤0)	2008	Tobacco control program	Birth outcomes	High
Markowitz (2008)[100]	ITS (USA)	All (<1)	2003	Smoke-free legislation, Cigarette price	Sudden infant death syndrome ─ incidence rate	Moderate
Markowitz (2013)[101]	CBA (USA)	All (≤1)	1998	Smoke-free legislation, Cigarette tax	Birth outcomes	High
Mayne et al. (2018)[102]	Longitudinal (USA)	All (18-30)	1998	Smoke-free legislation	Cardiovascular disease ─ incidence rate	High
Mayne et al. (2018)[103]	Longitudinal (USA)	All (33-45)	NA	Smoke-free legislation	Hypertension ─ incidence rate	High
McAlister et al. (2010)[104]	CBA (USA)	NA (NA)	2000	Tobacco control program	AMI ─ mortality rate	Moderate
McGhee et al. (2014)[105]	ITS (Hong-Kong)	All (0-65)	2006	Smoke-free legislation	Ischemic heart disease, AMI, cerebrovascular, cardiovascular, respiratory outcomes, lung cancer ─ hospitalization rate, mortality rate	High
McKinnon et al. (2015)[106]	CBA (Canada)	All (22-44 gestational weeks)	2006	Smoke-free legislation	Birth outcomes	High
Menzies et al. (2006)[107]	ITS (Scotland)	Male (22-48)	2006	Smoke-free legislation	Respiratory symptoms, sensory symptoms ─ prevalence	Moderate
Millett et al. (2013)[108]	ITS (England)	All (≤ 14)	2007	Smoke-free legislation	Asthma ─ hospitalization rate	Moderate
Moraros et al. (2010)[109]	ITS (USA)	All (≥18)	2002	Smoke-free legislation	AMI, Asthma – incidence rate	Moderate
Naiman et al. (2010)[110]	CBA (Canada)	All (≥45)	1999	Smoke-free legislation	AMI, angina, ischemic heart disease, asthma, COPD, Lung Infection ─ hospitalization rate	Moderate
Ozierański et al. (2019)[111]	ITS (Poland)	All (≥20)	2010	Smoke-free legislation	AMI ─ hospitalization rate	High
Page et al. (2012)[112]	ITS (USA)	Female (<18 - ≥ 35)	2003	Smoke-free legislation	Birth outcomes	High
Patanavanich et al. (2020)[113]	ITS (Thailand)	All (≥18)	1992	Smoke-free legislation, Tobacco tax	AMI ─ hospitalization rate	High
Patrick et al. (2016)[114]	ITS (USA)	All (<1)	NA	Tobacco tax, Cigarette price	Infant mortality rate	High

© 2023 Akter S et al. *JAMA Network Open.*

Peelen et al. (2016)[115]	ITS (Netherlands)	All (24-48 gestational weeks)	2004, 2008	Tobacco control program	Perinatal mortality, stillbirth ─ rate	High
Pell et al. (2008)[116]	Longitudinal (Scotland)	All (≤65)	2006	Smoke-free legislation	Acute coronary syndrome ─ hospitalization rate	High
Polus et al. (2021)[117]	ITS (Germany)	All (≤32 gestational weeks)	2008	Smoke-free legislation	Stillbirth rate	High
Rajkumar et al. (2014)[118]	Cohort (Switzerland)	All (18-65)	2010	Smoke-free legislation	Heart rate variability, pulse wave velocity	Low
Rodu et al. (2012)[119]	CBA (USA)	All (≥45)	1995	Smoke-free legislation	AMI ─ mortality rate	High
Sargent et al. (2004)[120]	CBA (USA)	All (≥18)	2002	Smoke-free legislation	AMI ─ hospitalization rate	Moderate
Sargent et al. (2012)[121]	Cohort (Germany)	All (≥30)	2007	Smoke-free legislation	Angina, AMI ─ hospitalization rate	High
Schoj et al. (2010)[122]	Longitudinal (Argentina)	All (21.8-46.3)	2007	Smoke-free legislation	Respiratory symptoms, sensory symptoms ─ prevalence	Moderate
Sebrie et al. (2013)[123]	ITS (Uruguay)	All (≥20)	2006	Smoke-free legislation	AMI ─ hospitalization rate	High
Seguret et al. (2014)[124]	ITS (France)	All (≥18)	2007	Smoke-free legislation	Acute coronary syndrome ─ hospitalization rate	High
Sen & Piérard (2011)[125]	CBA (Canada)	All (NA)	1994	Tobacco tax	Birth outcomes	High
Seo et al. (2007)[126]	Cross-sectional (USA)	NA (NA)	2003	Smoke-free legislation	AMI ─ hospitalization rate	High
Shelley et al. (2007)[127]	Cross-sectional (USA)	All (18-74)	2003	Smoke-free legislation	Health status (excellent or fair/poor)	High
Shetty et al. (2009)[128]	CBA (USA)	All (0-65)	1990	Smoke-free legislation	AMI ─ hospitalization rate, mortality rate	High
Simón et al. (2017)[129]	Cross-sectional (Spain)	All (26-42 gestational weeks)	2006-2011	Smoke-free legislation	Birth outcomes	High
Sims et al. (2010)[130]	ITS (England)	All (≥18)	2007	Smoke-free legislation	Myocardial infarction ─ hospitalization rate	High
Stallings-Smith et al. (2013)[131]	ITS (Ireland)	All (≥35)	2004	Smoke-free legislation	All, cardiovascular disease, Ischemic heart disease, AMI, stroke, and respiratory disease─ mortality risk	Low
Stallings-Smith et al. (2014)[132]	ITS (Ireland)	All (≥35)	2004	Smoke-free legislation	Ischemic heart disease, stroke, COPD ─ mortality rate	High

© 2023 Akter S et al. *JAMA Network Open.*

Thach et al. (2016)[133]	ITS (China)	All (≥0)	2007	Smoke-free legislation	Ischemic heart disease, AMI, cardiovascular disease, respiratory disease, lung cancer ─ mortality rate	Low
Trachsel et al. (2010)[134]	Cross-sectional (Switzerland)	All (≥0)	2008	Smoke-free legislation	AMI ─ hospitalization rate	High
Weg et al. (2012)[135]	CBA (USA)	All (≥65)	1991-2008	Smoke-free legislation	AMI ─ hospitalization rate	Low
Vicedo-Cabrera et al. (2016)[136]	ITS (Switzerland)	All (≥0)	2010	Smoke-free legislation	Cardiovascular, respiratory diseases ─ hospitalization rate, mortality rate	Low
Vicedo-Cabrera et al. (2016)[137]	ITS (Switzerland)	All (22-42 gestational weeks)	2010	Smoke-free legislation	Birth outcomes	High
Villalbi et al. (2011)[138]	Cross-sectional (Spain)	All (≥0)	2012	Smoke-free legislation	AMI ─ mortality rate	High
Weaver et al. (2018)[139]	ITS (USA)	All (≥0)	2012	Smoke-free legislation	AMI ─ hospitalization rate	Low
Wilson et al. (2012)[140]	Longitudinal (USA)	All (21-73)	2010	Smoke-free legislation	Respiratory symptoms ─ prevalence	Moderate
Wu et al. (2021)[141]	ITS (China)	All (≥18)	2015	MPOWER	Cardiovascular disease ─ hospitalization rate	High
Xiao et al. (2020)[142]	ITS (China)	All (≥35)	2013	Smoke-free legislation	AMI, stroke ─ mortality rate	Moderate
Xiao et al. (2020)[143]	ITS (China)	All (≥0)	2013	Smoke-free legislation	AMI, stroke ─ mortality rate	Moderate
Yan (2014)[144]	CBA (USA)	All (≤1)	1992	Minimum cigarette purchase age	Birth outcomes	Low
Yang et al. (2017)[145]	ITS (Taiwan)	All (≥45)	2009	Smoke-free legislation	Ischemic heart disease ─ hospitalization rate	High
Yildiz et al. (2014)[146]	Cross-sectional (Turkey)	NA (NA)	2009	Smoke-free legislation	Smoking-related diseases ─ emergency visits rate	High

[1]Based on study design (Newcastle-Ottawa Scale for observational studies and EPOC risk of bias for non-randomized studies)
CBA, controlled before and after study; ITS, interrupted time series; QE, quasi experimental studies
[2]NA, not available
[3]Policies are described in the original studies; Smoke-free legislations = "clean indoor air acts", i.e., laws banning or restricting smoking in shared spaces such as public place, workplace, restaurants, and bars; MPOWER = all six WHO-recommended tobacco control policies, i.e., monitoring tobacco use and prevention policies, protecting people from tobacco smoke, offering help to quit tobacco use, warning about the dangers of tobacco, enforcing bans on tobacco advertising and sponsorship, and raising taxes on tobacco; Tobacco control programs are composed of multiple interventions such as smoking ban, tax increase, mass media campaign, smoking cessation service, community and school programs, etc; Tobacco retailer density = number of tobacco retailers per population
[4]Outcomes are described in the original studies; AMI = Acute Myocardial Infarction; COPD = Chronic Obstructive Pulmonary Diseases

© 2023 Akter S et al. *JAMA Network Open.*

eFigure 2. Forest Plot of Meta-analysis of the Studies Examining the Associations of Smoke-free Legislation With Cardiovascular Disease (CVD), CVD Hospitalization, Respiratory Disease Hospitalization, CVD Mortality, Cancer, Respiratory Symptoms, Birth Weight, and Gestational Age Based on Coefficient Results

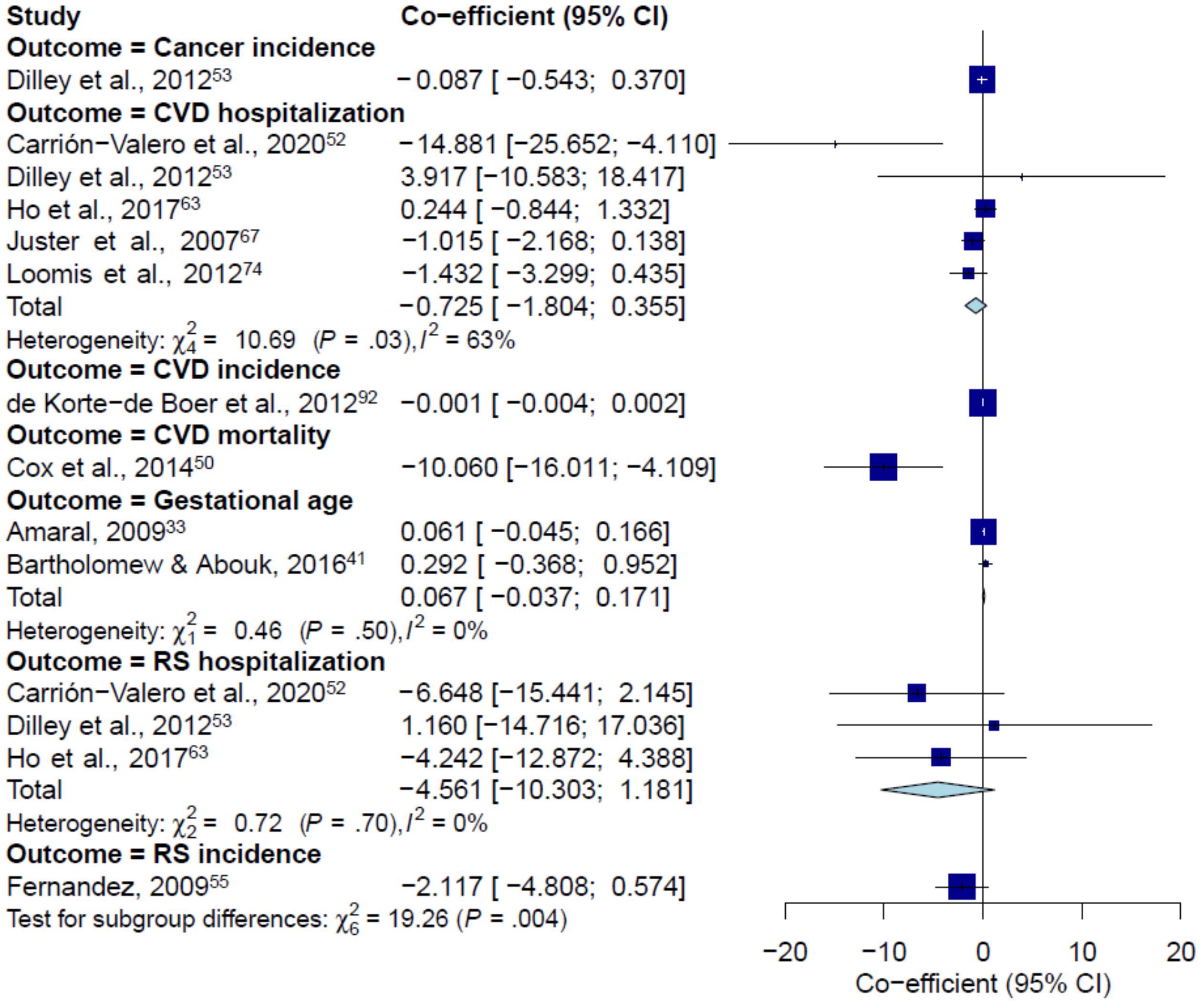

© 2023 Akter S et al. *JAMA Network Open.*

eFigure 3. Meta-analysis of the Association of Smoke-free Legislation With Perinatal Mortality and Adverse Birth Outcomes

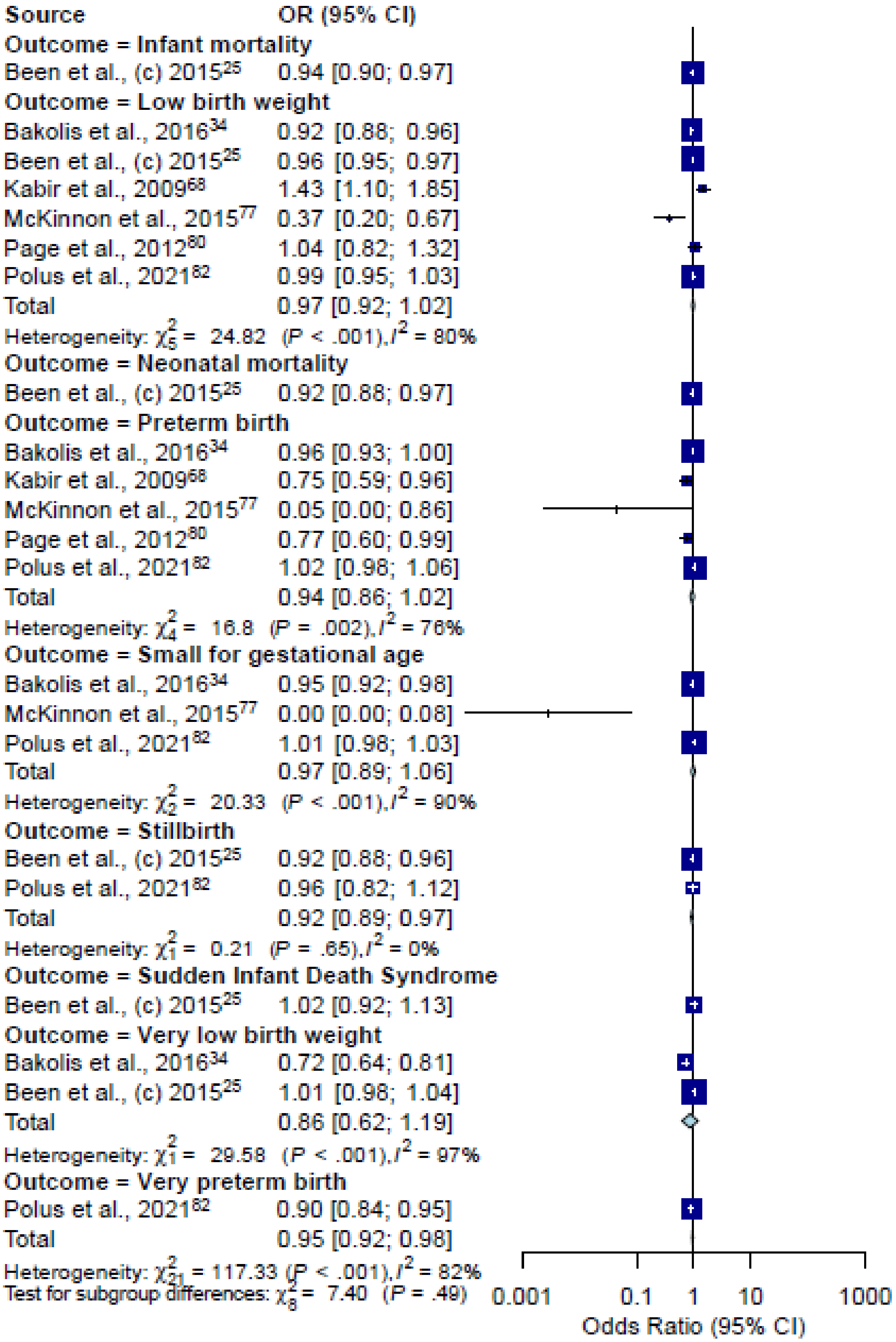

© 2023 Akter S et al. *JAMA Network Open.*

eFigure 4. Forest Plot of Meta-analysis of Studies Examining the Association of Tax or Price Increase With Hospitalization Due to Cardiovascular Disease or Respiratory Symptoms

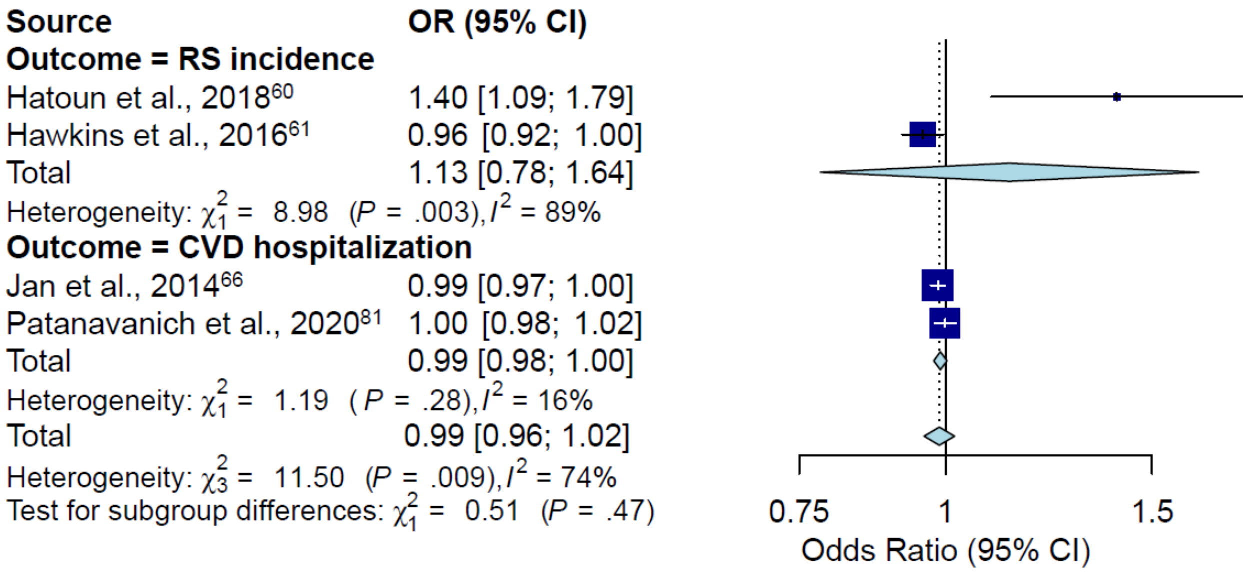

© 2023 Akter S et al. *JAMA Network Open.*

eFigure 5. Forest Plot of Meta-analysis of the Studies Examining the Association of Tax or Price Increase With Total Mortality, Cardiovascular Disease Mortality, Cancer Mortality, Respiratory Disease Mortality, Cardiovascular Disease Hospitalization, and Respiratory Disease Hospitalization Based on Coefficient Results

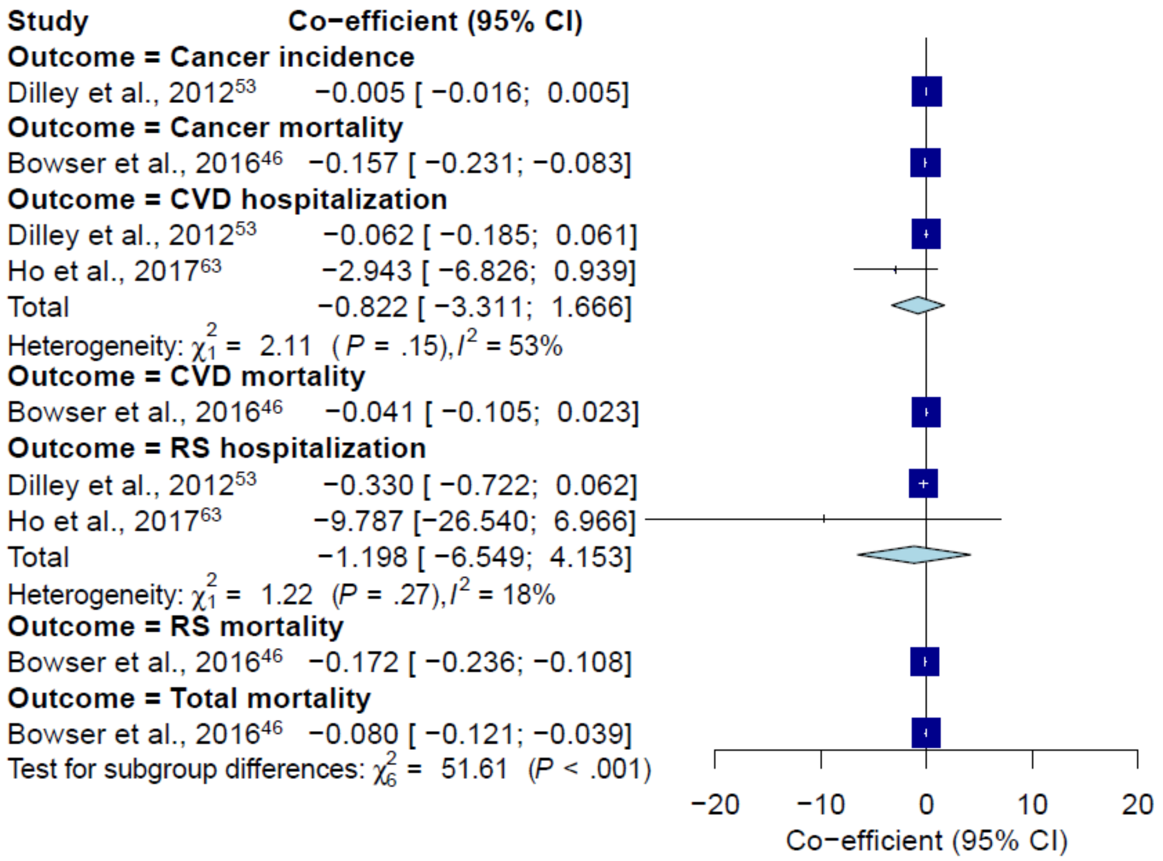

© 2023 Akter S et al. *JAMA Network Open.*

eFigure 6. Forest Plot of Meta-analysis of Studies Examining the Synergistic Associations of Tobacco Control Program (Combination of Several Tobacco Control Laws) With Cardiovascular Disease Hospitalization, Cardiovascular Disease Mortality, and Cancer Based on Coefficient Results

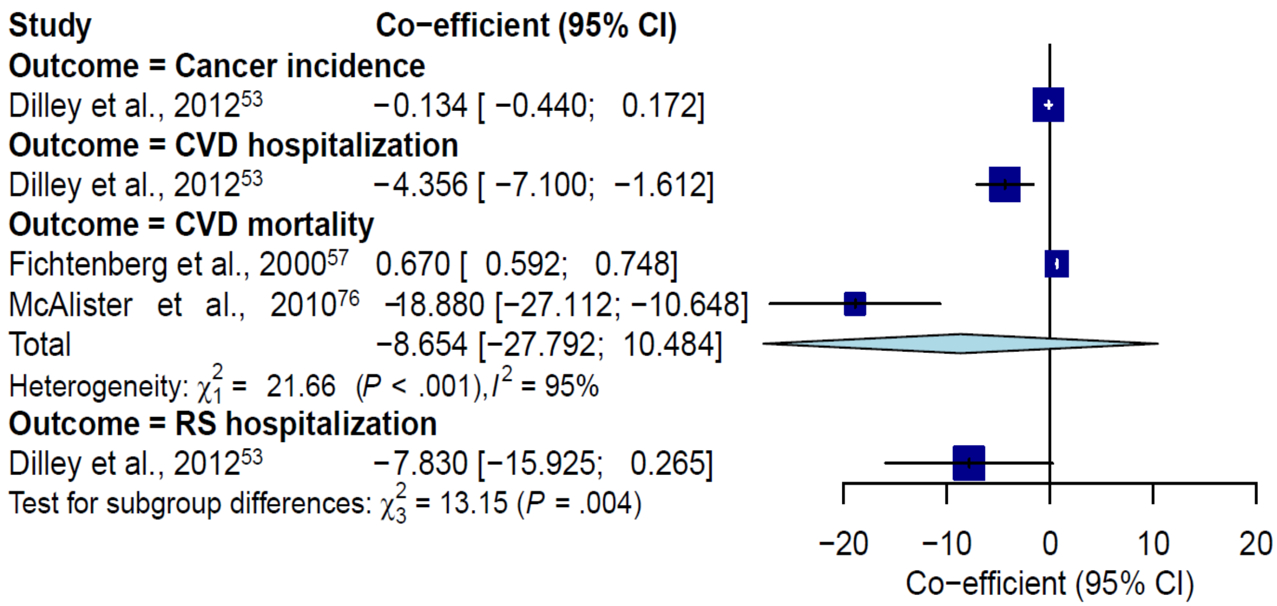

© 2023 Akter S et al. *JAMA Network Open.*

eAppendix 3. Sensitivity Analyses

Visual inspection of funnel plots indicated asymmetries in studies reporting the impact of smoke-free legislation on CVD events, respiratory diseases, birth outcomes, and CVD- and respiratory disease-related hospitalisations, which raises the possibility of publication bias (eFigures 7-9). To account for any publication bias, we performed sensitivity analyses using the trim-and-fill method (eFigures 7-9 and eTable 12). Results including the hypothetical studies showed a statistically significant negative association between smoke-free legislation and the occurrence of CVD events (OR, 0.92; 95% CI, 0.89-0.97), respiratory disease/symptom events (OR, 0.77; 95% CI, 0.67-0.89), adverse birth outcomes (OR, 95; 95% CI, 0.91-0.98), and hospitalisations due to respiratory disease/symptoms (OR, 0.91; 95% CI, 0.89-0.93). After dropping two highly influential studies (large sample size and small standard error), the smoke-free legislation remained significantly associated with reduced risks of CVD events, respiratory disease events, and hospitalisations due to CVD and respiratory diseases.

© 2023 Akter S et al. *JAMA Network Open.*

eFigure 7. Publication Bias Assessment for Cardiovascular Diseases

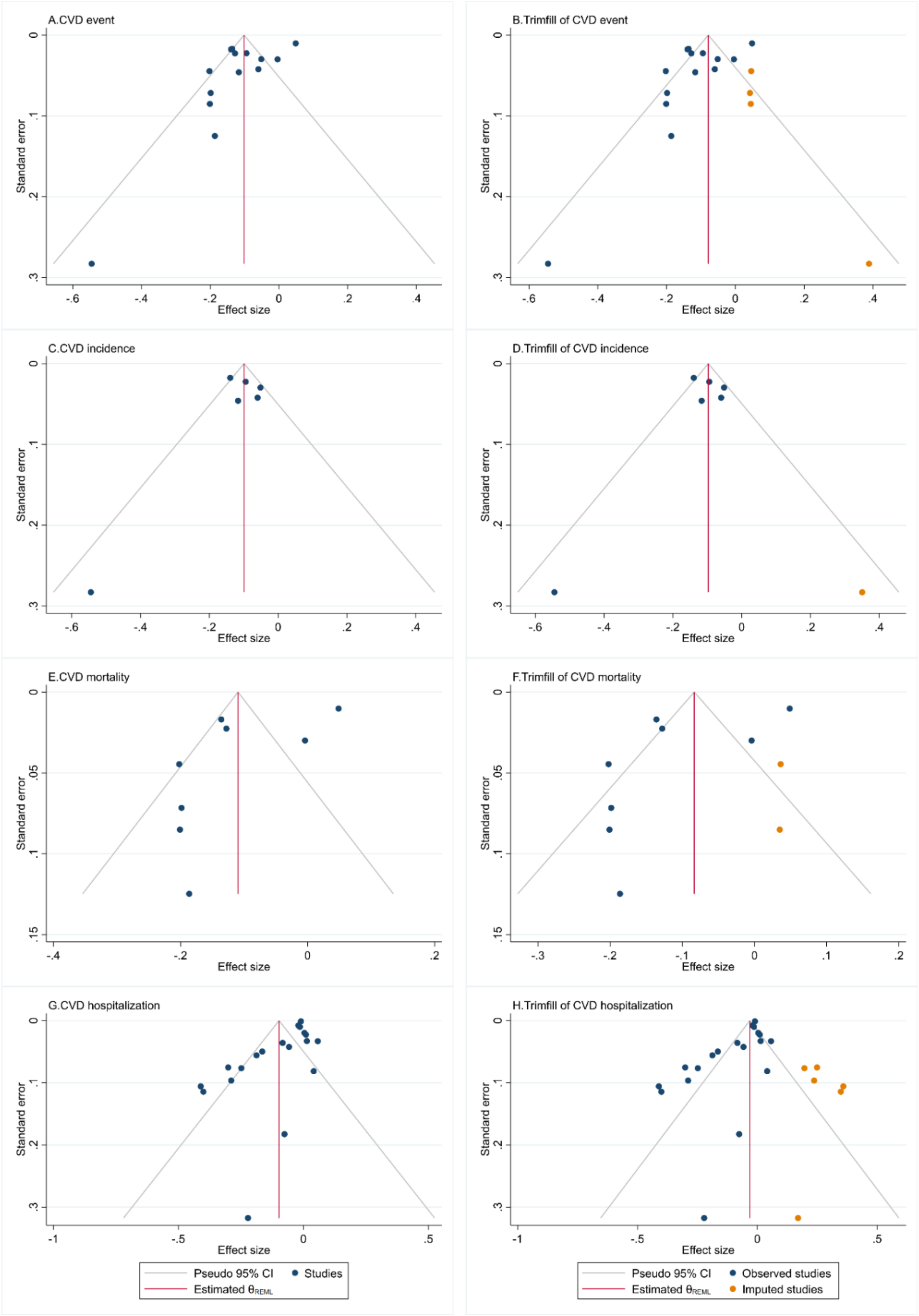

© 2023 Akter S et al. *JAMA Network Open.*

eFigure 8. Publication Bias Assessment for Smoke-free Legislation and Respiratory Disease and Symptoms

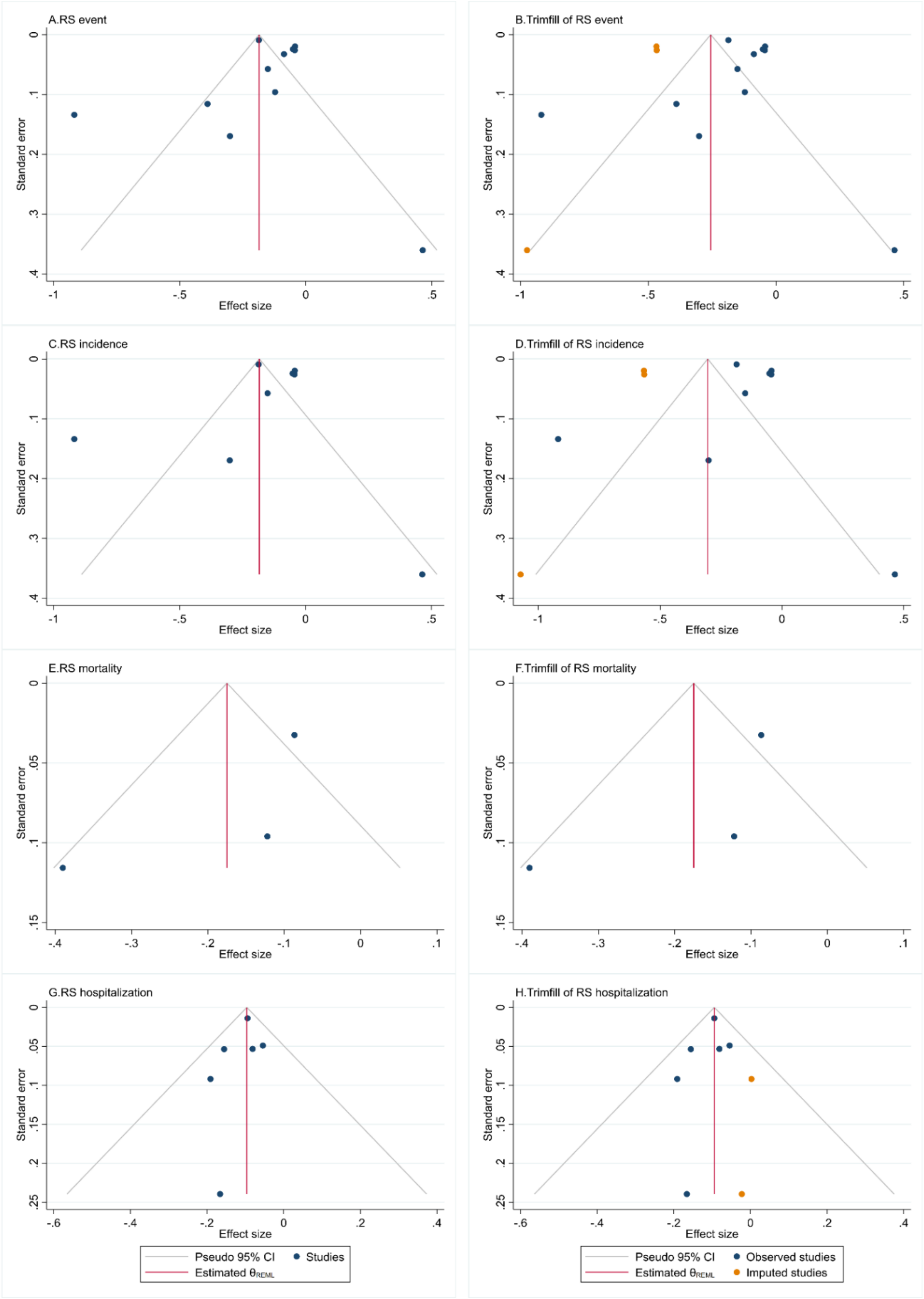

© 2023 Akter S et al. *JAMA Network Open.*

eFigure 9. Publication Bias Assessment for Smoke-free Legislation and Perinatal Mortality and Adverse Birth Outcomes

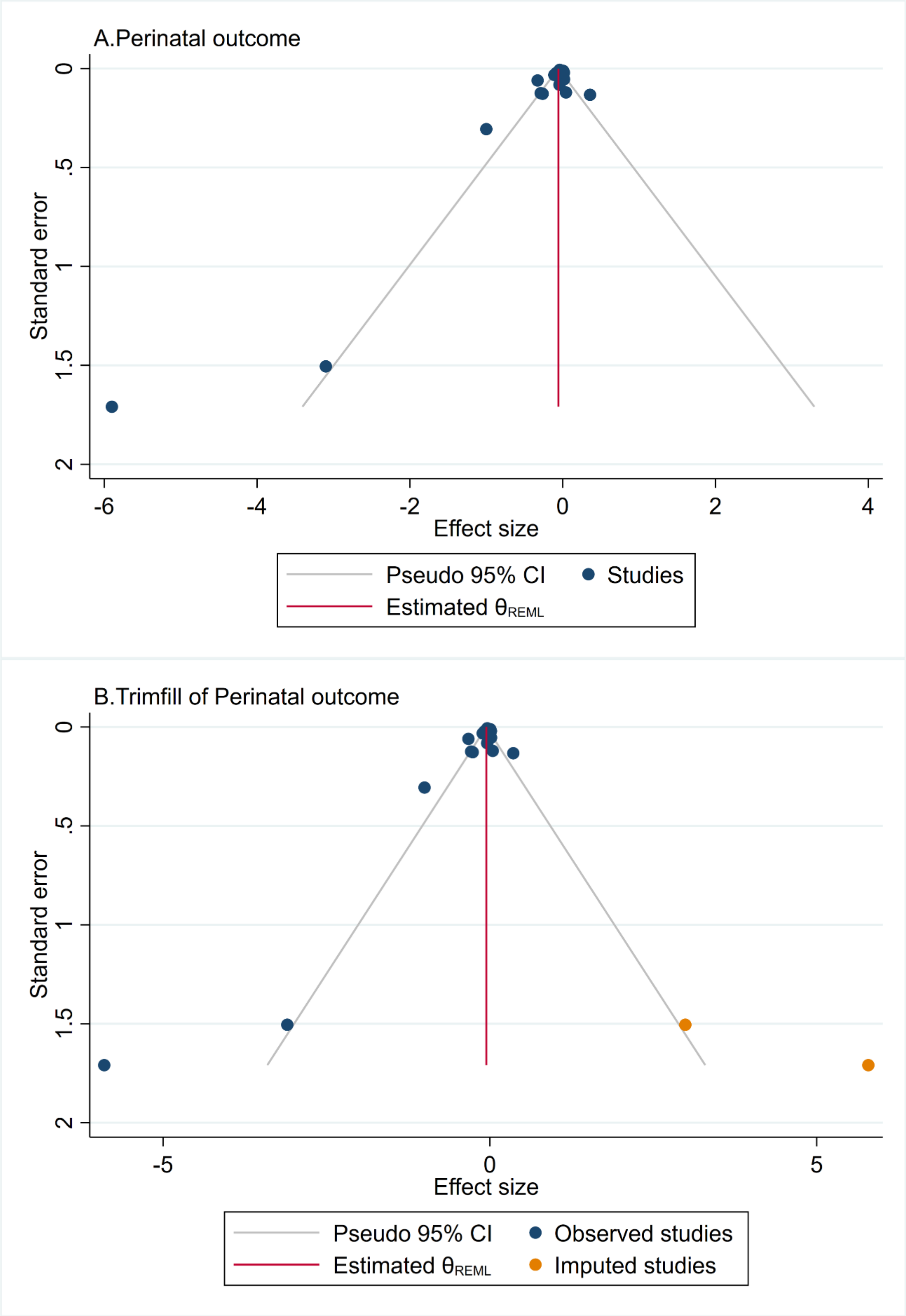

© 2023 Akter S et al. *JAMA Network Open.*

eTable 8. Summary, Publication Bias, and Trim-and-Fill Estimates

Outcome by smoke-free legislation	Summary estimates[a]			Sensitivity analysis[b]	Trim- and-fill estimates[c]	
	No. of studies	OR (95% CI)	*P*-bias test	OR (95% CI)	OR (95% CI)	N[d]
**Cardiovascular diseases**						
Incidence/Prevalence	6	0.91 (0.87-0.94)	0.291	0.93 (0.89-0.97)	0.91 (0.87-0.94)	1
Mortality	8	0.9 (0.83-0.97)	0.072	0.91 (0.87-0.94)	0.92 (0.86-0.98)	2
All (incidence/prevalence /mortality)	14	0.9 (0.86-0.94)	0.015	0.89 (0.86-0.92)	0.92 (0.89-0.97)	4
**Respiratory symptom**						
Incidence/Prevalence	8	0.83 (0.72-0.96)	0.861	0.81 (0.59-1.11)	0.74 (0.6-0.9)	3
Mortality	3	0.84 (0.71-0.99)	0.232	0.78 (0.6-1.01)	0.84 (0.71-0.99)	0
All (incidence/prevalence /mortality)	11	0.83 (0.72-0.96)	0.730	0.81 (0.67-0.99)	0.77 (0.67-0.89)	3
**Hospitalization**						
Cardiovascular diseases	19	0.91 (0.85-0.96)	0.000	0.89 (0.83-0.96)	0.97 (0.9-1.05)	6
Respiratory symptom	6	0.91 (0.89-0.93)	0.488	0.88 (0.82-0.94)	0.91 (0.89-0.93)	2
**Perinatal/birth outcome**	12	0.95 (0.91-0.98)	0.0001	0.86 (0.72-1.01)	0.95 (0.91-0.98)	2

[a]Pooled OR based on all studies

[b]Pooled OR based on after dropping two highly influential studies

[c]Trim-and-fill method simulated studies that were likely to be missing from the literature because of publication or other forms of bias. Trim-and-fill RRs estimate what the pooled RRs would be if the missing studies were included in the analysis.
[d]Missing studies

CI, confidence interval; PI, prediction interval

© 2023 Akter S et al. *JAMA Network Open.*

eAppendix 4. Stratified Analysis

We found evidence of large heterogeneity in the association between smoke-free legislation and CVD events, respiratory disease/symptom events, hospitalisation due to CVD and respiratory disease/symptoms, and adverse birth outcomes (eTable 9). In the stratified analysis, the ORs differed across country-income categories. The reduced risk of CVD events and hospitalisations due to CVD appeared more pronounced when smoke-free legislations were implemented in high-income countries. Subgroup analysis by quality score showed that smoke-free legislation was significantly associated with reduced risks of CVD events and CVD-related hospitalisations in high-quality studies only. However, neither the study design nor the variation in quality scores was found to explain the heterogeneity.

As it is suggested that health benefits vary according to the comprehensiveness of smoke-free legislation, we performed an additional subgroup analysis for comprehensive legislation (banning smoking in designated public facilities[147]) and partial legislation. We found a similar association in studies with comprehensive and partial smoke-free legislations (eTable 10).

© 2023 Akter S et al. *JAMA Network Open.*

eTable 9. Stratified Analysis According to Study Design, Country Income Category and Quality Score (For studies on smoke-free legislation policies).

Characteristic			P	
	No. of studies	Pooled RR (95% CI)	Heterogeneity, I^2(P value)	Meta regression
**CVD event**				
**Study design**				
CBA	1	0.87 (0.84-0.90)	NA	0.75
Cross-sectional	1	0.88 (0.84-0.92)	NA	
Interrupted Time-series	2	0.78 (0.55-1.12)	0.00	
Longitudinal	11	0.91 (0.87-0.96)	0.15	
**Country income category**				
Low and middle income	2	1.03 (0.98-1.08)	0.10	0.00
High income	12	0.88 (0.86-0.91)	0.07	
**Quality score**				
Low	NA			0.03
Moderate	6	0.94 (0.88-1.01)	0.00	
High	8	0.88 (0.85-0.91)	0.07	
**CVD mortality**				
**Study design**				
Cross-sectional	1	0.88 (0.84-0.92)	NA	0.96
Longitudinal	1	0.87 (0.84-0.9)	NA	
Interrupted Time-series	6	0.90 (0.81-1)	0.00	
**Country income category**				
Low and middle income	2	1.03 (0.98-1.08)	0.10	0.00
High income	6	0.87 (0.85-0.89)	0.61	
**Quality score**				
Low	NA			0.004
Moderate	3	0.96 (0.84-1.1)	0.00	
High	5	0.9 (0.83-0.97)	0.57	
**CVD hospitalization**				
**Study design**				
Cross-sectional	3	0.86 (0.77-0.97)	0.05	0.12
CBA	4	0.81 (0.66-0.99)	0.00	
Interrupted Time-series	12	0.99 (0.98-0.99)	0.00	
**Country income category**				

© 2023 Akter S et al. *JAMA Network Open.*

Low and middle income	2	1.01 (0.97-1.04)	0.66	0.18
High income	17	0.89 (0.83-0.95)	0.00	
**Quality score**				
Low	2	0.92 (0.68-1.24)	0.00	0.30
Moderate	4	0.98 (0.95-1.01)	0.14	
High	13	0.87 (0.8-0.95)	0.00	
**Respiratory disease event**				
**Study design**				
Cross-sectional	2	1.02 (0.49-2.12)	0.05	0.47
Longitudinal	2	0.61 (0.27-1.38)	0.00	
Interrupted Time-series	6	0.90 (0.83-0.97)	0.00	
Retrospective cohort	1	0.86 (0.77-0.96)	NA	
**Quality score**				
Low	1	0.68 (0.54-0.85)		0.35
Moderate	2	1.08 (0.7-1.69)	0.15	
High	8	0.81 (0.68-0.97)	0.00	
**Respiratory disease incidence**				
**Study design**				
Cross-sectional	2	1.02 (0.49-2.12)	0.05	0.00
Longitudinal	1	0.40 (0.31-0.52)	NA	
Interrupted Time-series	4	0.92 (0.86-0.99)	0.00	
Retrospective cohort	1	0.86 (0.77-0.96)	NA	
**Quality score**				
Low	NA			
Moderate	2	1.08 (0.70-1.69)	0.15	0.21
High	6	0.78 (0.60-0.99)	0.00	

© 2023 Akter S et al. *JAMA Network Open.*

eTable 10. Stratified Analysis According to Comprehensiveness of Smoke-free Legislation

Characteristic			P	
	**No. of studies**	**Pooled RR (95% CI)**	**Heterogeneity, I^2(P value)**	**Meta regression**
**CVD event**				
Comprehensive ban	7	0.91 (0.84-0.99)	0.00	0.48
Limited ban	7	0.89 (0.86-0.92)	0.05	
**Respiratory disease event**				
Comprehensive ban	3	0.85 (0.7-1.03)	0.01	0.95
Limited ban	8	0.83 (0.67-1.02)	0.00	
**Hospitalization due to CVD**				
Comprehensive ban	12	0.91 (0.84-0.98)	0.00	0.97
Limited ban	5	0.91 (0.81-1.01)	0.00	
**Hospitalization due to respiratory disease**				
Comprehensive ban	3	0.91 (0.88-0.93)	0.52	0.82
Limited ban	3	0.9 (0.81-1.01)	0.40	

© 2023 Akter S et al. *JAMA Network Open.*

eTable 11. Narrative Summary of Adverse Health Outcomes Following the Implementation of the Smoking Ban

Outcome	Study	Participants (Country)	Findings	Conclusion
**Cardiovascular Disease**				
**Acute Myocardial Infarction (AMI)**				
Hospitalization rate, mortality rate	Abe et al. 2017	NA (Brazil)	After implementation of the smoking ban, there was a reduction in myocardial infarction mortality rate (-11.9% after 17 months, p<0.001) and hospital admission rate (-5.4% after 3 months, p=0.022).	
Hospitalization rate	Barr et al. 2012	Adults (USA)	An immediate effect of the comprehensive smoking ban was a decrease in AMI hospitalization rate (-5.4%; 95% CI: -8.2 to -2.5)	
Hospitalization rate	Bonetti 2011	AMI patients (Switzerland)	In Graubünden, the number of patients with AMI significantly decreased two years after the implementation of the smoking ban (-21%; p<0.05).	
Hospitalization rate	Carrión-Valero et al. 2020	Adults (Spain)	After two successive smoking ban implementations in 2005 and 2010, there was an overall marked drop by 27% in hospital admission rates for AMI.	
Mortality rate	Dove et al. 2010	Adults (USA)	The AMI mortality rate significantly decreased by 7.4% (95% CI: 3.3% to 11.4%) after the implementation of the state smoking ban (p<0.001).	
Hospitalization rate	Galán et al. 2015	NA (Spain)	In Barcelona, a 6.3% (95% CI: -2.9 to 14.7) in hospital admissions for AMI following implementation of a partial smoking ban was not non-significant.	
Hospitalization rate	Galán et al. 2018	Adults (Spain)	There was no significant change in hospitalizations for AMI among patients aged ≥18years following implementation of partial (-18%; 95% CI: -6.7 to 3.5) and comprehensive (-2.3%; 95% CI: -5.2 to 0.6), p>0.05, smoking bans in Spain.	
Hospitalization rate	Gaudreau et al. 2013	NA (Canada)	After the smoking ban, the mean rate of admissions due to AMI significantly decreased by 5.92 cases per 100,000 person-months (95% CI: -0.39 to 11.44) (p=0.04), representing a 13.6% (p=0.03) immediately drop.	
Hospitalization rate	Herman & Welsh 2011	Adults (USA)	After statewide smoking ban implementation, there was a 13% (p=0.01) reduction in AMI hospital admissions. This estimate is based on drops in counties with only a statewide ban in place, compared to those with both statewide and county bans.	

© 2023 Akter S et al. *JAMA Network Open.*

Incidence rate	Johnson & Beal 2013	Hospitalized patients (USA)	Pre- to post- implementation of a comprehensive smoke-free law, the heart attack incidence rate significantly decreased, i.e., from 0.5% to 0.3% (p=0.023), respectively.	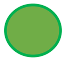
Mortality rate	Johnson & Beal 2013	Patients admitted for heart attacks (USA)	Pre- to post- implementation of a comprehensive smoke-free law, the heart attack mortality rate significantly decreased, i.e., from 2.5% to 1.8% (p<0.001), respectively.	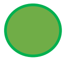
Prevalence	Lippert & Gustat 2012	Adults (USA)	Compared to the baseline estimates, seven states/territories recorded significant decreases in prevalence of AMI after implementation of smoking bans. Drops ranged between -1.4 and -0.4% (p<0.05).	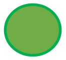
Hospitalization rate	Ozierański et al, 2019	Adults (Poland)	Following the introduction of the smoking ban, there were drops in AMI hospitalizations. Short-term changes ranged between -20.6 cases/100,000 among women aged ≥65 years to -0.4 cases/100,000 among women aged 20−64 years.	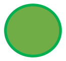
Mortality rate	Rodu et al. 2012	Adults (USA)	Following statewide smoking bans, two out five states recorded significant drops in AMI mortality in comparison with earlier projections. A 9% (p=0.04) decline in Florida and a 12% (p<0.0002) decline in New York.	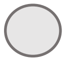
Hospitalization rate	Sargent et al. 2004	All patient (USA)	Six months after implementation of a local smoking bans, the number of AMI hospital admissions fell significantly (-16%, 95% CI: -31.7 to -0.3).	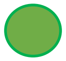
AMI, angina hospitalization rate	Sargent et al. 2012	Adults (Germany)	The law restricting smoking in the public and hospitality sectors was associated with a 13.3% (95% CI: 8.2 to 18.4) decline in angina pectoris and an 8.6% (95% CI: 5.0 to 12.2) decline in AMI after 1 year.	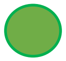
Hospitalization rate	Seo et al. 2007	Hospital-admitted patients (USA)	After the implementation of the smoking ban in public places, there was a significant drop in hospital admissions for AMI (-12%; 95% CI: -21.19 to -2.81) among nonsmoking patients.	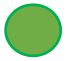
Hospitalization rate, mortality rate	Shetty et al. 2010	Hospital-admitted patients (USA)	Smoking bans were not associated with statistically significant short-term declines in neither mortality (-0.3%, 95% CI: -1.6 to 0.9, p<0.37) nor hospital admissions for AMI or other diseases (-1.5%, 95% CI: -4.8 to 1.8, p<0.37).	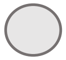
Hospitalization rate	Sims et al. 2010	Adults (UK)	There was a small but significant reduction in the number of emergency admissions for AMI after the implementation of the smoking ban (-2.4%, 95% CI: -4.06 to -0.66, p=0.007).	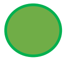
Hospitalization rate	Trachsel et al. 2010	Admitted patients (Switzerland)	Post implementation of the smoking ban, the number of AMI patients dropped to 183 (*p* <0.05), representing a 22% drop in the AMI incidence within the first year of enactment.	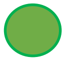
Hospitalization rate	Valentino et al. 2015	Adults (Switzerland)	The mean incidence of STEMI admissions during the 3 pre-ban years (123.7) was significantly higher than the incidence	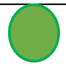

© 2023 Akter S et al. *JAMA Network Open.*

			of admissions in each of the 3 post-ban years (92.9, 101.6 and 89.6 respectively; p<0.024).	
Hospitalization rate	Weaver et al. 2018	Adults (USA)	After the enactment of the comprehensive smoke-free air law, monthly AMI admissions significantly declined by 20% (95% CI: 14 to 25) in Marion County and 25% (95% CI: 20 to 29) in Indianapolis.	
Hospitalization rate	Weg et al. 2012	Adults (USA)	Risk-adjusted hospital admission rates for acute myocardial infarction fell 20%–21% thirty-six months following implementation of new restaurant, bar, and workplace smoking bans.	
**Acute Coronary Syndrome (ACS)**				
Hospitalization rate	Abreu et al. 2017	Patients (Portugal)	There was a significant drop of -5.8% (p<0.001) in the crude rate of ACS hospital admissions after the smoking ban was rolled-out.	
Hospitalization rate	Cronin et al. 2012	Adults (Ireland)	In the year following implementation of the ban, there was a significant 12% reduction in ACS hospital admissions (p=0.002)	
Hospitalization rate	Ferrante et al. 2012	Adults (Argentina)	An immediate decrease in ACS admissions was observed after implementation of 100% smoke-free legislation in Santa Fe (-2.5 admissions per 100 000, p=0.03; 13% reduction)	
Hospitalization rate	Gupta et al. 2011	Adults (USA)	After the smoking ban, there was statistically insignificant rise in ACS hospital admission rates by 0.02% (95% CI: −0.08 to 0.11; p=0.12).	
Hospitalization rate	Pell et al. 2008	Hospital-admitted patients (Scotland)	Overall, after the enactment of the smoking ban in Scotland, the hospital admission rate for ACS decreased to 17% (95% CI: 16 to 18) compared to only a 4% drop in England which has no such legislation.	
**Coronary Heart Disease (CHD)**				
Hospitalization rate	Khuder et al. 2007	Adults (USA)	Hospital admission rates for CHD decreased significantly by 39% (95% CI: 33 to 45) after 1 year and by 47% (95% CI: 41 to 55) after 3 years following the implementation of the smoking ban (p=0.04).	
CHD, angina prevalence	Lippert & Gustat 2012	Adults (USA)	Compared to the baseline estimates, seven states/territories recorded significant decreases in prevalence of CHD or angina after implementation of smoking bans. Drops ranged between -1.4 and 0.6% (p<0.01).	
**Ischemic Heart disease (IHD)**				

© 2023 Akter S et al. *JAMA Network Open.*

Cerebrovascular disease hospitalization rate	Galán et al. 2015	NA (Spain)	Following implementation of the Spanish smoking ban, hospital admissions for cerebrovascular disease significantly decreased by 10.2% (95% CI: 3.8 to 16.1) in Barcelona, but not in Madrid.	
IHD hospitalization rate	Galán et al. 2018	Adults (Spain)	There was no significant change in hospitalizations for IHD among patients aged ≥18years following implementation of partial (0.1%; 95% CI: -3.6 to 4.0) and comprehensive (-2.6%; 95% CI: -5.6 to 0.5), p>0.05, smoking bans in Spain.	
Cerebrovascular disease hospitalization rate	Galán et al. 2018	Adults (Spain)	Neither partial (1.0%; 95% CI: -2.2 to 4.4) nor comprehensive (-0.8%; 95% CI: -2.9 to 1.4) smoking bans in Spain significantly reduce hospitalizations for cerebrovascular disease among patients ≥18years of age (p>0.05).	
Angina hospitalization rate	Gaudreau et al. 2013	NA (Canada)	After the smoking ban, there was a statistically insignificant change in the mean rate of admissions due to angina of 3.39% (95% CI: -12.85 to 19.63; p=0.68).	
Stroke hospitalization rate	Gaudreau et al. 2013	NA (Canada)	After the smoking ban, there was a statistically insignificant change in the mean rate of admissions due to stroke of 3.04% (95% CI: -7.06 to 13.14; p=0.56).	
Angina hospitalization rate	Herman & Welsh 2011	Adults (USA)	After statewide smoking ban implementation, there was a 33% (p=0.014) reduction in hospital admissions for unstable angina. This estimate is based on drops in counties with only a statewide ban in place, compared to those with both statewide and county bans.	
Stroke hospitalization rate	Herman & Welsh 2011	Adults (USA)	After statewide smoking ban implementation, there was a 14% (p=0.014) reduction in hospital admissions for acute stroke. This estimate is based on drops in counties with only a statewide ban in place, compared to those with both statewide and county bans.	
Stroke incidence rate	Mackay et al. 2013	Patients admitted to acute hospitals (Scotland)	Following smoke-free legislation, there was an 8.90% (95% CI: 4.85 to 12.77, p<0.001) stepwise reduction in cerebral infarction, but no visible effect was observed for other types of stroke.	
CVD incidence rate	Mayne et al. 2018	Young adults (USA)	Participants living in an area with a restaurant, bar, or workplace smoke-free policy had lower risk of incident cardiovascular disease (HR: 0.75, 95% CI: 0.49 to 1.15; HR: 0.76, 95% CI: 0.47 to 1.24; HR: 0.54, 95% CI: 0.34 to 0.86, respectively).	
IHD hospitalization rate	McGhee et al. 2014	Adult (Hong Kong)	After the introduction of the smoke-free law, the annual proportional change in hospital admissions for IHD in all ages dropped by 9% (95% CI: -13.59 to -4.17, p<0.05).	

© 2023 Akter S et al. *JAMA Network Open.*

CVD, ischemic stroke, AMI, angina hospitalization rate	Naiman et al. 2010	NA (Canada)	After a ban on smoking in restaurants came into effect, there was significant decrease of 39% (95% CI: 38 to 40; p<0.05) in hospital admissions due to cardiovascular conditions. 17% (95% CI: 14 to 19) of the decrease was due to AMI. The drops in ischemic stroke and angina were not statistically significant.	
Heart rate variability, pulse wave velocity	Rajkumar et al. 2014	Hospitality workers (Switzerland)	With the smoking ban, a one cigarette equivalents/day decrease was associated with a 2.3% (95% CI: 0.2, 4.4; p=0.031) higher root mean square of successive differences, a 5.7 % (95% CI: 0.9 to 10.2; p=0.02) higher high frequency component and a 0.72% (95 % CI: 0.40 to 1.05; p<0.001) lower pulse wave velocity.	
CVD, IHD, AMI, respiratory disease mortality rate	Thach et al. 2016	NA (China)	After the smoking ban in public and workplaces in Hong Kong, there was a significant decline in mortality due to IHD, AMI, CVD, and RD among all ages by 9.3%, 12.6%, 5.7%, and 5.4%, respectively (p<0.05).	
CVD hospitalization rate	Vicedo-Cabrera et al. 2016	NA (Switzerland)	Total hospitalization rates due to CVD did not significantly change (0.02%, CI: -2.71 to 2.82, p= 0.990) after the introduction of the smoking ban in public and workplaces.	
CVD mortality rate	Vicedo-Cabrera et al. 2016	NA (Switzerland)	Total mortality due to CVD did not significantly change (-0.72%, 95% CI: -3.67 to 2.31) after implementation of the smoking ban in public and workplaces.	
**Hypertension**				
Hypertension incidence rate	Mayne et al 2018	Adult (USA)	Hypertension rate did not significantly decreased after implementation of the smoking ban in bars, restaurants, and workplaces (18%, 7%, and 9% respectively)	
**Lung diseases**				
Respiratory symptoms (prevalence)	Allwright et al. 2005	Bar staff (Ireland)	Among bar staff who did not smoke, self-reported prevalence of any respiratory symptoms significantly declined (-16.7%, 95% CI: -26.1 to -7.3; p=0.001) after legislation for smoke-free workplaces	
Respiratory symptoms (prevalence)	Ayres et al. 2009	Bar workers (Scotland)	Self-reported respiratory symptoms among bar workers significantly reduced one year after their working environment became smoke-free from 69 % to 57% (-12%; p=0.02).	
Asthma hospitalization rate	Bianchi et al. 2011	Children (Italy)	Passage of the smoking ban was associated with a statistically significant decline of 30.7% (95% CI: 22.8 to 38.6) in the hospital admission rates for childhood asthma	

© 2023 Akter S et al. *JAMA Network Open.*

Respiratory symptoms (prevalence)	Durham et al. 2011	Hospitality workers (Switzerland)	The prevalence of chest oppression and shortness of breath decreased after the smoking ban (-3.85% and -1.96%, respectively); wheezing showed no change. The decrease in symptoms was, however, not statistically significant.	
Respiratory symptoms (prevalence)	Eagan et al. 2006	Hospitality workers (Norway)	Among non- and former-smokers, there were significant declines in reports of any respiratory symptoms (-11.9% and 33.3%, respectively, p<0.001) after the public smoking ban was passed.	
Respiratory symptoms (prevalence)	Eisner et al. 1998	Bartenders (USA)	After the smoking ban, the self-reported prevalence of any respiratory symptom significantly reduced among bartenders (-42%; p<0.001)	
Respiratory symptoms (prevalence)	Farrelly et al. 2005	Hospitality workers (USA)	After the smoking ban, there was no significant change in the prevalence of overall respiratory symptoms (-37%; p=0.117).	
COPD hospitalization rate	Galán et al. 2015	NA (Spain)	In Barcelona, hospital admissions for COPD were significantly reduced by 16% (95% CI: 7.0 to 24.1) following adoption of the smoking ban.	
Asthma hospitalization rate	Galán et al. 2015	NA (Spain)	In Barcelona, a 15.4% (95% CI: 8.4 to 34) drop in hospital admissions for asthma following adoption of the smoking ban was not statistically significant.	
COPD hospitalization rate	Galán et al. 2017	Adults (Spain)	The partial smoking ban was associated with an immediate drop of -14.7% (95% CI: -23.4 to -5.0) in COPD-related admissions, and was sustained over one-year (-13.6%; 95% CI: -23.1 to -2.9). The comprehensive ban showed no effect.	
Asthma hospitalization rate	Galán et al. 2017	Adults (Spain)	There was no immediate significant change in hospitalizations for asthma following implementation of partial (12.1%; 95% CI: -3.2 to 29.8) and comprehensive (-7.4%; 95% CI: -14.2 to -0.2) smoking bans. Long-term effects were also not statistically significant.	
COPD hospitalization rate	Gaudreau et al. 2013	NA (Canada)	After the smoking ban, the mean rate of admissions due to COPD significantly decreased by 6.6% (95% CI: -10.64 to 23.97; p=0.45).	
Asthma hospitalization rate (pediatric)	Gaudreau et al. 2013	NA (Canada)	After the smoking ban, a recorded increase of 1.11% (95% CI: 0.63 to 1.95; p=0.71) in the mean rate of admissions due to pediatric asthma was not statistically significant.	
Asthma hospitalization rate (adult)	Gaudreau et al. 2013	NA (Canada)	After the smoking ban, a recorded increase of 1.48% (95% CI: 0.90 to 2.41; p=0.12) in the mean rate of admissions due to adult asthma was not statistically significant.	
Respiratory symptoms (prevalence)	Goodman et al. 2007	Bar workers (Ireland)	There were significant reductions in self-reported respiratory symptoms after the smoking ban (-28%, p<0.01).	

© 2023 Akter S et al. *JAMA Network Open.*

Asthma hospitalization rate	Herman & Welsh 2011	Adults (USA)	After statewide smoking ban implementation, there was a 22% (p<0.001) reduction in hospital admissions for acute asthma. This estimate is based on drops in counties with only a statewide ban in place, compared to those with both statewide and county bans.	
Respiratory symptoms (prevalence)	Kim et al. 2015	Restaurant and pub workers (Korea)	Self-reported prevalence of respiratory symptoms did not significantly differ among staff in in Korean restaurants and pubs before and after the smoking ban (p>0.05).	
Asthma discharge rate	Landers 2014	Adult and Children (USA)	After the implementation of county smoke-free laws, reductions of asthma discharges were statistically significant (For Adult: b = -2.44; p<0.05; For Child: b = -1.32; p<0.05)	
Asthma discharge rate	Landers 2014	Adult and Children (USA)	After the implementation of state smoke-free laws, reductions in asthma discharges were not statistically significant (For Adult: b = 0.29; p>0.05; For Child: b = 0.12; p>0.05).	
Lower respiratory tract infection (LRTI) hospitalization rate	Lee 2016	Children (Hong Kong)	Implementation of comprehensive smoke-free legislation was associated with a significant reduction in hospital admissions for childhood LRTI (immediate effect: -33.5%; 95% CI: -36.4 to -30.5; trend effect: -13.9%; 95% CI: -16.0 to -11.7).	
Respiratory symptoms (prevalence)	Li et al. 2013	Occupational employees (China)	After the smoking ban, there was an overall statistically significant decrease in the prevalence of all respiratory symptoms (p<0.05). Drops ranged between -8.8% (p<0.01) for phlegm and 2.5% (p<0.05) for morning cough.	
Respiratory symptoms (prevalence)	MacCalman et al. 2012	Bar workers (UK)	There was a 15% and 23% reduction in any respiratory symptoms among bar workers after the implementation of smoke-free legislation in England and Scotland, respectively, (p<0.05).	
Asthma hospitalization rate	Mackay et al. 2010	Admitted children (Scotland)	The introduction of a comprehensive smoke-free legislation in all wholly or partially enclosed public spaces was associated with significant reductions in preterm delivery (-11.72%; 95% CI: -15.87 to -7.35; p<0.001).	
Respiratory symptoms (prevalence)	Madureira et al. 2014	Restaurant workers (Portugal)	There was a significant marked reduction in nasal problems (-88%, p<0.01), sore or dry throat, cough, and wheezing (-89%, p=0.07) after the implementation of the smoking ban.	
Sudden infant death syndrome (SIDS) incidence rate	Markowitz 2008	NA (USA)	Estimated effect by number of bans shows that each additional smoking ban is associated with an average reduction of 1.46 SIDS deaths.	
Respiratory problems hospitalization rate	McGhee et al. 2014	Adults (Hong Kong)	After the introduction of the smoke-free law, the annual proportional change in hospital admissions for respiratory problems for all ages increased by 2.55% (95% CI: 0.01 to 5.16; p<0.05).	

© 2023 Akter S et al. *JAMA Network Open.*

Lung cancer hospitalization rate	McGhee et al. 2014	Adult (Hong Kong)	After the introduction of the smoke-free law, the annual proportional change in hospital admissions for lung cancer for all ages increased by 14.33% (95% CI: 5.81 to 23.53; p<0.05).	
Lung cancer mortality rate	McGhee et al.2014	Adult (Hong Kong)	For lung cancer mortality, there was an immediate rate drop of -5.7% (95% CI: -9.73 to -1.39; p<0.05) after the smoking ban.	
Respiratory symptoms (prevalence)	Menzies et al. 2006	Adults (Scotland)	The percentage of bar workers with respiratory symptoms decreased from 79.2% before the smoke-free policy to 53.2% after the ban (total change, -26%; 95% CI: -13.8 to -38.1; p<0.001).	
Respiratory conditions (COPD, lung infection) hospitalization rate	Naiman et al. 2010	NA (Canada)	After a ban on smoking in restaurants came into effect, there was significant decrease of 33% (95% CI: 32 to 34; p<0.05) in hospital admissions due to respiratory conditions.	
Respiratory symptoms (prevalence)	Schoj et al. 2010	Adults (Argentina)	After the enactment of the 100% smoke-free law in the hospitality sector, there was a reduction in respiratory symptoms, from a pre-ban level of 57.5% to a post-ban level of 28.8% (p<0.001).	
Lung cancer, cerebrovascular disease mortality rate	Thach et al. 2016	NA (China)	After the smoking ban in public and workplaces in Hong Kong, there was no significant decline in mortality due to cerebrovascular disease (-3.4%; 95% CI: -8.4 to 1.8) and lung cancer (-3.4%; 95% CI: -8.2 to 1.8).	
Respiratory diseases (COPD, asthma, pneumonia) hospitalization rate	Vicedo-Cabrera et al. 2016	NA (Switzerland)	Total hospitalization rates due to respiratory diseases did not significantly change (0.03%; 95% CI: -5.67 to 6.06; p= 0.993) after the introduction of the smoking ban in public and workplaces.	
Respiratory diseases mortality rate	Vicedo-Cabrera et al. 2016	NA (Switzerland)	Total mortality due to respiratory diseases significantly decreased (-8.2%; 95% CI: -15.2 to -0.6; p<0.03) after the implementation of the smoking ban in public and workplaces.	
Hospitalization rate	Weg et al. 2012	Adults (USA)	Admission rates for COPD fell 11% where workplace smoking bans were in place and 15% where bar smoking bans were present.	
Respiratory symptoms (prevalence)	Wilson et al. 2012	Adults (USA)	After the adoption Michigan's Dr. Ron Davis smoke-free air law, there were significant decreases in respiratory symptoms. These ranged between -1 point (p<0.001) for allergic symptoms to -0.2 point (p<0.05) for wheezing based on a five-point likert-type scale.	
Bronchitis, lower respiratory tract infections emergency visits rate	Yildiz et al. 2014	NA (Turkey)	Emergency admissions decreased significantly by 39.8% (p<0.05) for bronchitis and 4.4% (p<0.01) for lower respiratory tract infections after the enactment of smoke-free legislation.	
COPD, myocardial infarction emergency visits rate	Yildiz et al. 2014	NA (Turkey)	A 21.2% and 2.4% decrease recorded in emergency unit admissions due to COPD and myocardial infarction,	

© 2023 Akter S et al. *JAMA Network Open.*

			respectively, after a legislation banning smoking in all indoor public places was not statistically significant (p>0.05).	
Asthma emergency visits rate	Yildiz et al. 2015	NA (Turkey)	After a legislation banning smoking in all indoor public places, a 16.0% increase in emergency unit admissions due to asthma was not statistically significant (p>0.05).	
**Birth outcomes**				
Birth weight (mean), low birth weight, very low birth weight prevalence	Amaral 2009	Infants (USA)	Estimated changes in birth weight, low birth weight, very low birth weight of -0.34 grams, 0.06 percentage points (%p), and -0.04 %p, respectively, after local smoking bans, and 2.78 grams, 0.04 %p, and -0.03 %p, respectively, after statewide smoking bans were not statistically significant (p>0.05).	
Low birth weight, very low birth weight prevalence	Bakolis et al. 2016	Pregnant women (UK)	The risk of low birth weight decreased between 8% (95% CI: 4 to 12) and 14% (95% CI: 5 to 23), and very low birth weight between 28% (95% CI: 19 to 36) and 32% (95% CI: 21 to 41) following the introduction of the smoke-free legislation in England, for those entering their third trimester.	
Birth weight (mean), low birth weight, very low birth weight, preterm birth prevalence	Bharadwaj et al. 2014	Female hospitality workers (Norway)	Children of female workers in restaurants and bars born after the smoking ban saw significantly lower rates (1.8 percentage point (%p); p<0.05) of babies born below the very low birth weight threshold. Changes in birth weight (+54.9 grams), low birth weight (+0.01 %p), and preterm birth (+2.5 %p) was not statistically significant.	
Birth weight (mean), low birth weight, very low birth weight prevalence	Gao & Baughman 2017	NA (USA)	Smoking bans were not associated with small improvements in low birth weight for babies born to mothers aged 20–24 years (1.46%) and very low birth weight for babies born to mothers aged 14–19 years (4.94%) and 20–24 years (2.52%), p<0.05. Effects on birth weight were not statistically significant.	
Birth weight, low birth weight, very low birth weight, preterm birth, very preterm birth prevalence, infant mortality rate	Hajdu & Hajdu 2018	Mothers (Hungary)	Following the smoking ban, mean birth weight of newborns increased by 1.7% or 55.5g, while low birth weight, very low birth weight, preterm birth, very preterm birth and infant mortality rate decreased by 2.2 percentage point (%p), 1.2 %p, 1.9 %p, 0.9 %p and 0.5 %p, respectively (p<0.05).	
Low birth weight, very low birth weight, preterm birth prevalence	Hankins & Tarasenko 2016	NA (USA)	Smoking bans had no statistically significant effects either birth outcomes. Among smoking mothers, slight decreases were seen in low birth weight for restaurant bans (-0.05%), in very low birth weight for workplace bans (-0.02%), and preterm births (-0.21%), p>0.05. Nonsmoking mothers	

© 2023 Akter S et al. *JAMA Network Open.*

			recorded decreases in very low birth weight for workplace bans (-0.01%) and preterm births (-0.02%), p>0.05.	
Birth weight, low birth weight, preterm birth, small for gestational age	Hawkins et al. 2014	Mothers (USA)	Smoke-free restaurant legislation was not associated with adverse birth outcomes.	
Birth defect, Cleft lip with or without cleft palate, cleft palate alone, limb reduction defect, cyanotic congenital heart disease, gastroschisis, or any other birth defects	Hawkins and Baum, 2019	Mothers (USA)	Enactment of smoke-free legislation was not associated with birth defects (p>0.05).	
Birth weight, low birth weight, preterm birth, small for gestational age	Hawkins and Baum, 2019	Mothers (USA)	No evidence was found for associations between the enactment of smoke-free legislation and all birth outcomes, except preterm birth, such that smoke-free legislation was associated with a 5.60 percentage point increase in preterm birth (3.70, 7.50; p<0.01).	
Infant, neonatal mortality rate	Hone et al. 2020	Infant (Brazil)	Comprehensive smoke-free legislation implementation was associated with -5.2 % (95% CI: -8.3 to -2.1, p<0.001) and -3.4 % (95% CI: -6.7 to -0.1, p<0.05) step reductions in infant and neonatal mortality, respectively.	
Small-for-gestational-age (SGA) prevalence	Kabir et al. 2013	Women with singleton livebirths (Ireland)	There was a significant immediate decline in SGA rates after the smoking ban (-0.45%; 95% CI: -0.7 to -0.19, p<0.0007), which was sustained over time with a gradual effects estimated at -0.02% (p<0.0001).	
Preterm delivery, small for gestational age	Mackay et al. 2012	Women with singleton births (Scotland)	Three months prior to the legislation, there were significant decreases in small for gestational age (−4.52%, 95% CI= −8.28, −0.60, p=0.024) and overall preterm delivery (−11.72%, 95% CI= −15.87, −7.35, p<0.001).	
Birth weight (mean), small-for-gestational-age (SGA), premature birth prevalence	Mallma et al. 2020	Women with singleton births (Peru)	After implementation of the tobacco control laws, there statistically significant reduction in prevalence of premature births by 30 cases per 10,000 live births. Decreases of -3.10 grams in birthweight and 6 cases per 10,000 live births in SGA were statistically insignificant.	
Birth weight (mean), low birth weight, very low birth weight prevalence	Markowitz et al. 2013	Women with singleton births (USA)	Complete smoking bans in restaurant showed no statistically significant effects on birth outcomes. There were increases ranging between 2.8 and 6.6 %p for birth weight, slight drops between -0.3 and -0.4 %p for low birth weight, and 0.1 %p each for very low birth weight among women aged ≥20 years (p>0.05). All indicators moved in the adverse direction for women aged <20 years.	

© 2023 Akter S et al. *JAMA Network Open.*

Preterm birth, low birth weight prevalence, small-for-gestational-age (SGA)	Simón et al. 2017	Mothers (Spain)	Comprehensive smoking ban was associated with immediate and one year after implementation reductions in preterm birth (4.5% vs 4.1%) and low birth weight (2.3% vs 3.5%) rates. Partial smoking ban reduced SGA immediately (4.9%) and one year post-implementation (4.2%).	
Preterm, early-term births prevalence	Vicedo-Cabrera et al. 2016	Newborn Children (Switzerland)	After the implementation of the smoking ban in public places and workplaces in Switzerland, the risk of preterm birth and early term birth was significantly reduced [preterm birth: -3.6% (95% CI: -9.3 to 2.5); early-term birth: -5.0% (95% CI: -7.5 to -2.5).	
**Health status**				
Self-reported health status	Shelley et al. 2007	Adult Chinese American (USA)	Smokers who live under a total household smoking ban only or both a total household and total workplace ban were, respectively, 1.90 (CI: 0.99 to 3.67, $p<0.05$) and 2.61 (CI: 1.22 to 4.08, $p<0.01$) times more likely to report better health status compared with those who reported no smoking ban at work or home.	

Policy: Smoke-free legislations = “clean indoor air acts”, i.e., laws banning restricting smoking in shared spaces such as public place, workplace, restaurants, bars
Outcomes: AMI, Acute Myocardial Infarction; ACS, Acute Coronary Syndrome; IHD, Ischemic Heart Disease; CVD, Cardiovascular disease; STEMI, ST-elevation myocardial infarctions; COPD, Chronic Obstructive Pulmonary Disease; LBW, Low birth weight; VLBW, Very low birth weight; SGA, Small-for-gestational-age
Metrics: CI, confidence interval; IRR, Incidence Rate Ratio; OR, odds ratio; RR, risk ratio/relative risk
Note:
Green: Positive effect of tobacco policy, i.e., statistically significant increase or decrease in targeted outcome favoring the policy
Red: Negative effect of tobacco policy, i.e., statistically significant increase or decrease in targeted outcome favoring control
Grey: No effect of tobacco policy, i.e., statistically non-significant increase or decrease in targeted outcome

© 2023 Akter S et al. *JAMA Network Open.*

eTable 12. Narrative Summary of Adverse Health Outcomes Following the Implementation of Tobacco Law[1]

Outcome	Study	Participants (Country)	Impact	Conclusion
**Mortality**				
Smoking-attributable mortality rate	Holford et al. 2014	NA (USA)	Estimated smoking-attributable deaths were reduced after tobacco control measures[1] (-31%; 95% CI: -32 to -29).	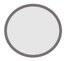
Cancer mortality rate	Jiang et al. 2019	Youth and adults (Australia)	The release of UK and US public health reports on tobacco in 1962 and 1964 and the ban on cigarette ads on TV and radio in 1976 were found to have been associated with a reduction in mortality from all cancer types except liver cancer.	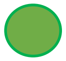
**Heart Diseases**				
CVD hospitalization rate	Wu et al. 2021	Adults (China)	Hospital admission rates for all CVDs decreased significantly, immediately after implementing the comprehensive tobacco policy[2], (-4%; 95% CI: -5.3 to -2.7).	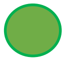
**Lung Diseases**				
Lung cancer	Barnoya and Glantz, 2004	NA (USA)	A comprehensive tobacco control program is associated with a lower incidence of lung cancer (p=0.001).	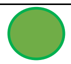
**Birth Outcomes**				
Small-for-gestational age (SGA) prevalence	Peelen et al. 2016	Infant (singleton birth) (Netherland)	The smoking ban in bars and restaurants, mass media campaign, and tobacco tax increase was associated with a statistically significant decrease in SGA (-4.4%; 95% CI: -2.4 to -6.4; p<0.001)	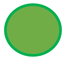
Perinatal mortality rate, preterm birth, stillbirth, early neonatal mortality, very preterm birth, low birth weight, very low birth weight	Peelen et al. 2016	Infant (singleton birth) (Netherland)	The 2004 and 2008 policies of the smoking ban in workplaces, mass media campaigns, and tobacco tax increase were not associated with statistically significant changes in the odds of developing perinatal mortality, preterm birth, stillbirth, early neonatal mortality, very preterm birth, low birth weight, and very low birth weight.	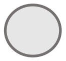

[1]Tobacco control measures include—education on smoking's dangers, increases in cigarette taxes, smoke-free air laws, media campaigns, marketing, and sales restrictions, lawsuits, and cessation treatment programs
[2]Comprehensive tobacco policy package: combining a complete ban on smoking in indoor public places, cessation support, more comprehensive bans on advertising, and tax rises
Outcomes: CVD, Cardiovascular disease; SGA, Small-for-gestational-age. Metrics: CI, confidence interval
Note:
Green: Positive effect of tobacco policy, i.e., statistically significant increase or decrease in targeted outcome favoring the policy
Red: Negative effect of tobacco policy, i.e., statistically significant increase or decrease in targeted outcome favoring control
Grey: No effect of tobacco policy, i.e., statistically non-significant increase or decrease in targeted outcome

© 2023 Akter S et al. *JAMA Network Open.*

eTable 13. Narrative Summary of Adverse Health Outcomes Following the Implementation of an Increase in Cigarette Prices or Cigarette Taxes

**Outcome**	**Study**	**Participants (Country)**	**Impact**	**Conclusion**
**Lung and heart diseases**				
Sudden infant death syndrome (SIDS) prevalence	Markowitz 2008	NA (USA)	Higher cigarette prices and state excise taxes are negatively and significantly associated with a reduction in SIDS deaths. A 10% increase in the real price of cigarettes reduces SIDS deaths by 6.7%-7.4%. Every 10% increase in the real taxes on cigarettes reduces SIDS deaths by 1.56%-1.79%.	
AMI, asthma, sudden cardiac death (SCD) hospitalization rate	Ma et al. 2013	Adult (USA)	An increase in the price of cigarettes to more than $4 per 20-cigarette pack was associated with a significant decrease in age-adjusted asthma hospitalization (p<0.001) and age-adjusted AMI hospitalization (p<0.05), and SCD rates (p=0.001).	
**Birth outcome**				
Birth weight (mean)	Evans and Ringel, 1999	Women with live births (USA)	A one-cent increase in the state tax rate on cigarettes increases average birth weight by 0.16 g (p<0.05).	
Birth weight, low birth weight, preterm birth, small for gestational age	Hawkins et al. 2014	Mothers (USA)	Increases in cigarette taxes were associated with increased birth weight and a reduced number of adverse birth outcomes (low birth weight, preterm birth and small for gestational age).	
Birth defect, Cleft lip with or without cleft palate, cleft palate alone, limb reduction defect, cyanotic congenital heart disease, gastroschisis, or any other birth defects	Hawkins and Baum, 2019	Mothers (USA)	Every $1.00 increase in cigarette taxes reduced the risk of infants having any birth defect by 0.0023 percentage points (p=0.02) and also reduced the risks of cyanotic heart defects (p=0.04), cleft palate (p=0.04), gastroschisis (p=0.02), and limb reduction (p=0.02).	
Birth weight, low birth weight, preterm birth, small for gestational age	Hawkins and Baum, 2019	Mothers (USA)	Among white mothers, every $1.00 increase in cigarette taxes increased their infants' birth weight by 4.19 g (p=0.003) and decreased the likelihood of having a baby born low birth weight by -0.05 percentage points (p=0.01), preterm by -0.04 percentage points (p=0.02), or small for gestational age by -0.18 percentage points (p=0.008).	
Birth weight (mean), Low birth weight, Very low birth weight prevalence	Markowitz et al. 2013	Women with singleton births (USA)	Cigarettes taxes showed no statistically significant effects on birth outcomes. With each $1 increase in the real tax on cigarettes, there were increases ranging between 1.0 and 5.0 %p for birth weight, drops between -0.1 and -0.6 %p for low birth weight, and between -0.2 and -0.3 %p for very low birth weight among women aged <20	

© 2023 Akter S et al. *JAMA Network Open.*

			to 34 years (p>0.05). All indicators moved in the adverse direction for women aged ≥35 years.	
Infant mortality rate	Patrick et al. 2016	Infant (USA)	With every $1 increase in cigarette price per pack, there was an overall change in infant mortality rates of -0.19% (95% CI: -0.30 to -0.09).	●
Fetal deaths, low birth weight, infant mortality	Sen and Pierard, 2011	NA (Canada)	Higher taxes are significantly correlated with lower infant mortalities and higher fetal deaths.	●

Outcomes: SIDS, Sudden Infant Death Syndrome; Asthma, SCD, Sudden cardiac death. Metrics: CI, confidence interval

Note:

Green: Positive effect of tobacco policy, i.e., statistically significant increase or decrease in targeted outcome favoring the policy
Red: Negative effect of tobacco policy, i.e., statistically significant increase or decrease in targeted outcome favoring control
Grey: No effect of tobacco policy, i.e., statistically non-significant increase or decrease in targeted outcome

© 2023 Akter S et al. *JAMA Network Open.*

eTable 14. Narrative Summary of Adverse Health Outcomes Following the Implementation of Miscellaneous Policies

**Outcome**	**Study**	**Participants (Country)**	**Impact**	**Conclusion**
Minimum Cigarette Purchase Age				
**Birth outcome**				
Birth weight (mean), low birth weight, preterm birth incidence	Yan 2014	Women who conceived between ages 19 to 21 years (USA)	Pregnant women subject to the minimum cigarette purchase age of 21 years (MCPA 21) policy were 1.5 percentage points (%p) ($p<0.05$) less likely to have low birth weight babies. Increases in birth weight of 11.4 gram and preterm terms of 1.2 %p given MCPA 21 were not statistically significant ($p>0.05$).	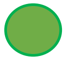
Nicotine Replacement Therapy (NRT)				
**Birth outcome**				
Birth weight, gestational age at delivery	Adams et al. 2013	Women with live births (USA)	Coverage for NRT, medications and cessation counseling during pregnancy was associated with a small increase (<1 day) in infant gestation ($p<0.05$).	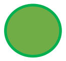
Preterm birth, small for gestational age	Jarlenski et al. 2014	Mothers (USA)	The comprehensive smoking cessation coverage of pharmacotherapy (any form of NRT or bupropion for smoking cessation) and counseling did not have a significant effect on the probability of having a preterm birth or an infant small for gestational age.	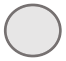
Tobacco Retailer Density				
**Lung Diseases**				
COPD-related inpatient hospital discharge outcomes	Kong et al. 2021	NA (USA)	Higher retailer density was associated with a 19% (IRR=1.19; 95% CI=1.12–1.27) higher COPD-related hospital discharge rate and 30% (IRR=1.30; 95% CI=1.21–1.39) higher total COPD-related hospital costs per population.	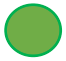

Note:Outcome: COPD, Chronic Obstructive Pulmonary Disease
Green: Positive effect of tobacco policy, i.e., statistically significant increase or decrease in targeted outcome favoring the policy
Red: Negative effect of tobacco policy, i.e., statistically significant increase or decrease in targeted outcome favoring control
Grey: No effect of tobacco policy, i.e., statistically non-significant increase or decrease in targeted outcome.

After implementation of free/discounted nicotine replacement therapy, 1 out of 2 studies reported a significant improvement in birth outcomes. The only study evaluating the impact of the minimum cigarette purchase age law found that it was associated with a lower prevalence of low birth weight, while another study evaluating the impact of tobacco retailer density found that higher retailer density was associated with higher discharge rates for chronic obstructive pulmonary disease (COPD)

© 2023 Akter S et al. *JAMA Network Open.*

eReferences

1. Wells G SB, O'Connell D, Peterson J, Welch V, Losos M, et al. The Newcastle-Ottawa Scale (NOS) for assessing the quality of nonrandomised studies in meta-analyses. *https://wwwohrica/programs/clinical_epidemiology/oxfordasp* 2015.
2. Effective Practice and Organization of Care (EPOC): EPOC resources for review authors. *https://epoccochraneorg/resources/epoc-resources-review-authors*.
3. Abe TMO, Scholz J, de Masi E, Nobre MRC, Filho RK. Decrease in mortality rate and hospital admissions for acute myocardial infarction after the enactment of the smoking ban law in São Paulo city, Brazil. *Tob Control.* 2017; **26**(6): 656-662.
4. Abreu D, Sousa P, Matias-Dias C, Pinto FJ. Longitudinal Impact of the Smoking Ban Legislation in Acute Coronary Syndrome Admissions. *Biomed Res Int.* 2017; **2017**: 6956941.
5. Adams EK, Markowitz S, Dietz PM, Tong VT. Expansion of Medicaid covered smoking cessation services: maternal smoking and birth outcomes. *Medicare Medicaid Res Rev*. 2013; **3**(3).
6. Aguero F, Degano IR, Subirana I, et al. Impact of a partial smoke-free legislation on myocardial infarction incidence, mortality and case-fatality in a population-based registry: the REGICOR Study. *PLoS One.* 2013; **8**(1): e53722.
7. Allwright S, Paul G, Greiner B, et al. Legislation for smoke-free workplaces and health of bar workers in Ireland: before and after study. *BMJ.* 2005; **331**(7525): 1117.
8. Alsever RN, Thomas, W. M., Nevin-Woods, C et al. Reduced hospitalizations for acute myocardial infarction after implementation of a smoke-free ordinance--City of Pueblo, Colorado, 2002-2006. *MMWR Morb Mortal Wkly Rep.* 2009; **57**(51): 1373-1373.
9. Amaral M. The effect of local smoking ordinances on fetal development: evidence from California. Accessed June 2, 2023.
10. Ayres JG, Semple S, MacCalman L, et al. Bar workers' health and environmental tobacco smoke exposure (BHETSE): symptomatic improvement in bar staff following smoke-free legislation in Scotland. *Occupl Environ Med.* 2009; **66**(5): 339-346.
11. Bakolis I, Kelly R, Fecht D, et al. Protective effects of smoke-free legislation on birth outcomes in England: A regression discontinuity design. *Epidemiology.* 2016; **27**(6): 810-818.
12. Bannon F, Devlin A, McElwee G, Gavin A. Greater gains from smoke-free legislation for non-smoking bar staff in Belfast. *Eur J of Public Health.* 2009; **19**(6): 638-643.
13. Barnett R, Pearce J, Moon G, Elliott J, Barnett P. Assessing the effects of the introduction of the New Zealand Smokefree Environment Act 2003 on acute myocardial infarction hospital admissions in Christchurch, New Zealand. *Aust N Z J Public Health.* 2009; **33**(6): 515-520.
14. Barnoya J, Glantz S. Association of the California tobacco control program with declines in lung cancer incidence. *Cancer causes & control : CCC* 2004; **15**(7): 689-95.
15. Barone-Adesi F, Vizzini L, Merletti F, Richiardi L. Short-term effects of Italian smoking regulation on rates of hospital admission for acute myocardial infarction. *Eur Heart J.* 2006; **27**(20): 2468-2472.
16. Barone-Adesi F, Gasparrini A, Vizzini L, Merletti F, Richiardi L. Effects of Italian smoking regulation on rates of hospital admission for acute coronary events: a country-wide study. *PLoS One* 2011; **6**(3): e17419.
17. Barr CD, Diez DM, Wang Y, Dominici F, Samet JM. Comprehensive smoking bans and acute myocardial infarction among Medicare enrollees in 387 US counties: 1999-2008. *Am J Epidemiol.* 2012; **176**(7): 642-648.
18. Barrio G, Belza MJ, Carmona R, Hoyos J, Ronda E, Regidor E. The limits of single-group interrupted time series analysis in assessing the impact of smoke-free laws on short-term mortality. *Int J Drug Policy.* 2019; **73**: 112-120.

19. Bartecchi C, Alsever RN, Nevin-Woods C, et al. Reduction in the incidence of acute myocardial infarction associated with a citywide smoking ordinance. *Circulation.* 2006; **114**(14): 1490-1496.
20. Bartholomew KS, Abouk R. The effect of local smokefree regulations on birth outcomes and prenatal smoking. *Mater Child Health J.* 2016; **20**(7): 1526-1538.
21. Basel P, Bartelson BB, Le Lait MC, Krantz MJ. The effect of a statewide smoking ordinance on acute myocardial infarction rates. *Am J Med.* 2014; **127**(1): 94 e1-94.e6.
22. Been JV, Millett C, Lee JT, van Schayck CP, Sheikh A. Smoke-free legislation and childhood hospitalisations for respiratory tract infections. *Eur Respir J.* 2015; **46**(3): 697-706.
23. Been JV, Szatkowski L, van Staa TP, et al. Smoke-free legislation and the incidence of paediatric respiratory infections and wheezing/asthma: interrupted time series analyses in the four UK nations. *Sci Rep.* 2015; **5**: 15246.
24. Been JV, Mackay DF, Millett C, Pell JP, van Schayck OC, Sheikh A. Impact of smoke-free legislation on perinatal and infant mortality: a national quasi-experimental study. *Sci Rep.* 2015; **5**: 13020.
25. Bharadwaj P, Johnsen JV, Løken KV. Smoking bans, maternal smoking and birth outcomes. *J Public Econ.* 2014; **115**: 72-93.
26. Bianchi M, Campi R, Bonati M. Smoke-free legislation and asthma. *N Engl J Med.* 2011; **364**(1): 87-88.
27. Bonetti PO, Trachsel LD, Kuhn MU, et al. Incidence of acute myocardial infarction after implementation of a public smoking ban in Graubünden, Switzerland: two year follow-up. *Swiss Med Wkly.* 2011; **141**: w13206.
28. Bowser D, Canning D, Okunogbe A. The impact of tobacco taxes on mortality in the USA, 1970-2005. *Tob Control.* 2016; **25**(1): 52-59.
29. Bruintjes G, Bartelson BB, Hurst P, Levinson AH, Hokanson JE, Krantz MJ. Reduction in acute myocardial infarction hospitalization after implementation of a smoking ordinance. *Am J Med.* 2011; **124**(7): 647-654.
30. Carrión-Valero F, Quiles-Izquierdo J, González-Monte C, et al. Association between a comprehensive smoking ban and hospitalization for acute myocardial infarction: an observational study in the Autonomous Community of Valencia, Spain. *Rev Port Cardiol (Engl Ed).* 2020; **39**(2): 77-84.
31. Carrión-Valero F, Quiles-Izquierdo J, González-Monte C, et al. Impact of the 2005 and 2010 Spanish smoking laws on hospital admissions for tobacco-related diseases in Valencia, Spain. *Public Health.* 2020; **180**: 29-37.
32. Cesaroni G, Forastiere F, Agabiti N, Valente P, Zuccaro P, Perucci CA. Effect of the Italian smoking ban on population rates of acute coronary events. *Circulation.* 2008; **117**(9): 1183-8.
33. Ciaccio CE, Gurley-Calvez T, Shireman TI. Indoor tobacco legislation is associated with fewer emergency department visits for asthma exacerbation in children. *Ann Allergy Asthma Immunol.* 2016; **117**(6): 641-645.
34. Cox B, Vangronsveld J, Nawrot TS. Impact of stepwise introduction of smoke-free legislation on population rates of acute myocardial infarction deaths in Flanders, Belgium. *Heart.* 2014; **100**(18): 1430-1435.
35. Croghan IT, Ebbert JO, Hays JT, et al. Impact of a countywide smoke-free workplace law on emergency department visits for respiratory diseases: a retrospective cohort study. *BMC Pulm Med.* 2015; **15**: 6.
36. Cronin EM, Kearney PM, Kearney PP, Sullivan P, Perry IJ. Impact of a national smoking ban on hospital admission for acute coronary syndromes: a longitudinal study. *Clin Cardiol.* 2012; **35**(4): 205-209.
37. de Korte-de Boer D, Kotz D, Viechtbauer W, et al. Effect of smoke-free legislation on the incidence of sudden circulatory arrest in the Netherlands. *Heart.* 2012; **98**(13): 995-9.

© 2023 Akter S et al. *JAMA Network Open.*

38. Dilley JA, Harris JR, Boysun MJ, Reid TR. Program, policy, and price interventions for tobacco control: quantifying the return on investment of a state tobacco control program. *Am J Public Health.* 2012; **102**(2): e22-8.
39. Di Valentino M, Muzzarelli S, Limoni C, et al. Reduction of ST-elevation myocardial infarction in Canton Ticino (Switzerland) after smoking bans in enclosed public places--No Smoke Pub Study. *Eur J Public Health.* 2015; **25**(2): 195-199.
40. Dove MS, Dockery DW, Mittleman MA, et al. The impact of Massachusetts' smoke-free workplace laws on acute myocardial infarction deaths. *Am J Public Health.* 2010; **100**(11): 2206-12.
41. Dove MS, Dockery DW, Connolly GN. Smoke-free air laws and asthma prevalence, symptoms, and severity among nonsmoking youth. *Pediatrics.* 2011; **127**(1): 102-9.
42. Durham AD, Bergier S, Morisod X, et al. Improved health of hospitality workers after a Swiss cantonal smoking ban. *Swiss Med Wkly.* 2011; **141**: w13317.
43. Eagan TM, Hetland J, Aarø LE. Decline in respiratory symptoms in service workers five months after a public smoking ban. *Tob Control.* 2006; **15**(3): 242-6.
44. Eisner MD, Smith AK, Blanc PD. Bartenders' respiratory health after establishment of smoke-free bars and taverns. *JAMA.* 1998; **280**(22): 1909-1914.
45. Evans WN, Ringel JS. Can higher cigarette taxes improve birth outcomes? *J Public Econ.* 1999; **72**(1): 135-54.
46. Farrelly MC, Nonnemaker JM, Chou R, Hyland A, Peterson KK, Bauer UE. Changes in hospitality workers' exposure to secondhand smoke following the implementation of New York's smoke-free law. *Tob Control.* 2005; **14**(4): 236-241.
47. Fernández E, Fu M, Pascual JA, et al. Impact of the Spanish smoking law on exposure to second-hand smoke and respiratory health in hospitality workers: a cohort study. *PLoS One.* 2009; **4**(1): e4244.
48. Ferrante D, Linetzky B, Virgolini M, Schoj V, Apelberg B. Reduction in hospital admissions for acute coronary syndrome after the successful implementation of 100% smoke-free legislation in Argentina: a comparison with partial smoking restrictions. *Tob Control.* 2012; **21**(4): 402-406.
49. Fichtenberg CM, Glantz SA. Association of the California Tobacco Control Program with declines in cigarette consumption and mortality from heart disease. *N Engl J Med.* 2000; **343**(24): 1772-1777.
50. Galán I, Simón L, Flores V, et al. Assessing the effects of the Spanish partial smoking ban on cardiovascular and respiratory diseases: methodological issues. *BMJ Open.* 2015; **5**(12): e008892.
51. Galán I, Simón L, Boldo E, et al. Changes in hospitalizations for chronic respiratory diseases after two successive smoking bans in Spain. *PLoS One.* 2017; **12**(5): e0177979.
52. Galán I, Simón L, Boldo E, et al. Impact of 2 Successive Smoking Bans on Hospital Admissions for Cardiovascular Diseases in Spain. *Rev Esp Cardiol (Eng Ed).* 2018; **71**(9): 726-734.
53. Gao J, Baughman RA. Do Smoking Bans Improve Infant Health? Evidence from U.S. Births: 1995–2009. *East Econ J.* 2017; **43**(3): 472-495.
54. Gasparrini A, Gorini G, Barchielli A. On the relationship between smoking bans and incidence of acute myocardial infarction. *Eur J Epidemiol.* 2009; **24**(10): 597-602.
55. Gaudreau K, Sanford CJ, Cheverie C, McClure C. The effect of a smoking ban on hospitalization rates for cardiovascular and respiratory conditions in Prince Edward Island, Canada. *PLoS One.* 2013; **8**(3): e56102.
56. Goodman P, Agnew M, McCaffrey M, Paul G, Clancy L. Effects of the Irish smoking ban on respiratory health of bar workers and air quality in Dublin pubs. *Am J Respir Crit Care Med*. 2007; **175**(8): 840-5.
57. Gupta R, Luo J, Anderson RH, Ray A. Clean indoor air regulation and incidence of hospital admissions for acute coronary syndrome in Kanawha County, West Virginia. *Prev Chronic Dis*. 2011; **8**(4): A77.

© 2023 Akter S et al. *JAMA Network Open.*

58. Hahn EJ, Rayens MK, Burkhart PV, Moser DK. Smoke-free laws, gender, and reduction in hospitalizations for acute myocardial infarction. *Public Health Rep.* 2011; **126**(6): 826-833.
59. Hajdu T, Hajdu G. Smoking ban and health at birth: Evidence from Hungary. *Econ Hum Biol*. 2018; **30**: 37-47.
60. Hankins S, Tarasenko Y. Do smoking bans improve neonatal health? *Health Serv Res*. 2016; **51**(5): 1858-78.
61. Hatoun J, Davis-Plourde K, Penti B, Cabral H, Kazis L. Tobacco control laws and pediatric asthma. *Pediatrics.* 2018; **141**(Suppl 1): S130-s6.
62. Hawkins SS, Baum CF, Oken E, Gillman MW. Associations of tobacco control policies with birth outcomes. *JAMA Pediatr.* 2014; **168**(11): e142365.
63. Hawkins SS, Hristakeva S, Gottlieb M, Baum CF. Reduction in emergency department visits for children's asthma, ear infections, and respiratory infections after the introduction of state smoke-free legislation. *Prev Med.* 2016; **89**: 278-85.
64. Hawkins SS, Baum CF. The downstream effects of state tobacco control policies on maternal smoking during pregnancy and birth outcomes. *Drug Alcohol Depend.* 2019; **205**: 107634.
65. Hawkins SS, Baum CF. Impact of state tobacco control policies on birth defects. *Prev Med.* 2019; **127**: 105791.
66. Head P, Jackson BE, Bae S, Cherry D. Hospital discharge rates before and after implementation of a city-wide smoking ban in a Texas city, 2004-2008. *Prev Chronic Dis.* 2012; **9**: E179.
67. Herman PM, Walsh ME. Hospital admissions for acute myocardial infarction, angina, stroke, and asthma after implementation of Arizona's comprehensive statewide smoking ban. *Am J Public Health*. 2011; **101**(3): 491-6.
68. Ho V, Ross JS, Steiner CA, et al. A nationwide assessment of the association of smoking bans and cigarette taxes with hospitalizations for acute myocardial infarction, heart failure, and pneumonia. *Med Care Res Rev.* 2017; **74**(6): 687-704.
69. Holford TR, Meza R, Warner KE, et al. Tobacco control and the reduction in smoking-related premature deaths in the United States, 1964-2012. *JAMA*. 2014; **311**(2): 164-71.
70. Hone T, Szklo AS, Filippidis FT, et al. Smoke-free legislation and neonatal and infant mortality in Brazil: longitudinal quasi-experimental study. *Tob Control*. 2020; **29**(3): 312-9.
71. Humair JP, Garin N, Gerstel E, et al. Acute respiratory and cardiovascular admissions after a public smoking ban in Geneva, Switzerland. *PLoS One.* 2014; **9**(3): e90417.
72. Hurt RD, Weston SA, Ebbert JO, et al. Myocardial infarction and sudden cardiac death in Olmsted County, Minnesota, before and after smoke-free workplace laws. *Arch Intern Med.* 2012; **172**(21): 1635-41.
73. Jan C, Lee M, Roa R, Herrera V, Politis M, Motta J. The association of tobacco control policies and the risk of acute myocardial infarction using hospital admissions data. *PLoS One.* 2014; **9**(2): e88784.
74. Jarlenski M, Bleich SN, Bennett WL, Stuart EA, Barry CL. Medicaid enrollment policy increased smoking cessation among pregnant women but had no impact on birth outcomes. *Health Aff (Millwood)*. 2014; **33**(6): 997-1005.
75. Jiang H, Livingston M, Room R, Gan Y, English D, Chenhall R. Can public health policies on alcohol and tobacco reduce a cancer epidemic? Australia's experience. *BMC Med.* 2019; **17**(1): 213.
76. Johnson EL, Beal JR. Impact of a comprehensive smoke-free law following a partial smoke-free law on incidence of heart attacks at a rural community hospital. *Nicotine Tob Res*. 2013; **15**(3): 745-747.
77. Juster HR, Loomis BR, Hinman TM, et al. Declines in hospital admissions for acute myocardial infarction in New York state after implementation of a comprehensive smoking ban. *Am J Public Health*. 2007; **97**(11): 2035-2039.
78. Kabir Z, Clarke V, Conroy R, McNamee E, Daly S, Clancy L. Low birthweight and preterm birth rates 1 year before and after the Irish workplace smoking ban. *BJOG.* 2009; **116**(13): 1782-1787.

79. Kabir Z, Daly S, Clarke V, Keogan S, Clancy L. Smoking ban and small-for-gestational age births in Ireland. *PLoS One.* 2013; **8**(3): e57441.
80. Kalkhoran S, Sebrié EM, Sandoya E, Glantz SA. Effect of Uruguay's National 100% Smokefree Law on Emergency Visits for Bronchospasm. *Am J Prev Med*. 2015; **49**(1): 85-8.
81. Kent BD, Sulaiman I, Nicholson TT, Lane SJ, Moloney ED. Acute pulmonary admissions following implementation of a national workplace smoking ban. *Chest.* 2012; **142**(3): 673-679.
82. Khuder SA, Milz S, Jordan T, Price J, Silvestri K, Butler P. The impact of a smoking ban on hospital admissions for coronary heart disease. *Prev Med.* 2007; **45**(1): 3-8.
83. Kim J, Kwon HJ, Lee K, et al. Air Quality, Biomarker Levels, and Health Effects on Staff in Korean Restaurants and Pubs Before and After a Smoking Ban. *Nicotine Tob Res*. 2015; **17**(11): 1337-1346.
84. Kong AY, Baggett CD, Gottfredson NC, Ribisl KM, Delamater PL, Golden SD. Associations of tobacco retailer availability with chronic obstructive pulmonary disease related hospital outcomes, United States, 2014. *Health Place.* 2021; **67**: 102464.
85. Landers G. The impact of smoke-free laws on asthma discharges: a multistate analysis Am J Public Health. 2014; **104**(2): e74-e79.
86. Larsson M, Boëthius G, Axelsson S, Montgomery SM. Exposure to environmental tobacco smoke and health effects among hospitality workers in Sweden--before and after the implementation of a smoke-free law. *Scand J Work Environ Health*. 2008; **34**(4): 267-77.
87. Lee SL, Wong WH, Lau YL. Smoke-free legislation reduces hospital admissions for childhood lower respiratory tract infection. *Tob Control.* 2016; **25**(e2): e90-e4.
88. Lemstra M, Neudorf C, Opondo J. Implications of a public smoking ban. *Can J Public Health.* 2008; **99**(1): 62-65.
89. Li X, Gao J, Zhang Z, et al. Lessons from an evaluation of a provincial-level smoking control policy in Shanghai, China. *PLoS One.* 2013; **8**(9): e74306.
90. Lippert WC, Gustat J. Clean Indoor Air Acts reduce the burden of adverse cardiovascular outcomes. *Public Health.* 2012; **126**(4): 279-85.
91. Liu A, Guzman Castillo M, Capewell S, Lucy J, O'Flaherty M. Reduction in myocardial infarction admissions in Liverpool after the smoking ban: potential socioeconomic implications for policymaking. *BMJ Open.* 2013; **3**(11): e003307.
92. Loomis BR, Juster HR. Association of indoor smoke-free air laws with hospital admissions for acute myocardial infarction and stroke in three states. *J Environ Public Health*. 2012; **2012**: 589018.
93. Ma ZQ, Kuller LH, Fisher MA, Ostroff SM. Use of interrupted time-series method to evaluate the impact of cigarette excise tax increases in Pennsylvania, 2000-2009. *Prev Chronic Dis*. 2013; **10**: E169.
94. MacCalman L, Semple S, Galea KS, et al. The relationship between workers' self-reported changes in health and their attitudes towards a workplace intervention: lessons from smoke-free legislation across the UK hospitality industry. *BMC Public Health.* 2012; **12**: 324.
95. Mackay D, Haw S, Ayres JG, Fischbacher C, Pell JP. Smoke-free legislation and hospitalizations for childhood asthma. *N Engl J Med.* 2010; **363**(12): 1139-1145.
96. Mackay DF, Nelson SM, Haw SJ, Pell JP. Impact of Scotland's smoke-free legislation on pregnancy complications: retrospective cohort study. *PLoS medicine.* 2012; **9**(3): e1001175.
97. Mackay DF, Haw S, Newby DE, et al. Impact of Scotland's comprehensive, smoke-free legislation on stroke. *PLoS One.* 2013; **8**(5): e62597.
98. Madureira J, Mendes A, Teixeira JP. Evaluation of a smoke-free law on indoor air quality and on workers' health in Portuguese restaurants. *J Occup Environ Hyg.* 2014; **11**(4): 201-209.
99. Mallma P, Carcamo C, Kaufman JS. The impact of anti-tobacco legislation on birth weight in Peru. *Glob Health Res Policy*. 2020; **5**: 5.
100. Markowitz S. The effectiveness of cigarette regulations in reducing cases of Sudden Infant Death Syndrome. *J Health Econ*. 2008; **27**(1): 106-133.

© 2023 Akter S et al. *JAMA Network Open.*

101. Markowitz S, Adams, E. K., Dietz, P. M., Tong, V. T., & Kannan, V. Tobacco control policies, birth outcomes, and maternal human capital. *J Hum Cap.* 2013; **7**(2): 130–160.
102. Mayne SL, Widome R, Carroll AJ, et al. Longitudinal associations of smoke-free policies and incident cardiovascular disease: CARDIA study. *Circulation.* 2018; **138**(6): 557-66.
103. Mayne SL, Jacobs DR, Jr., Schreiner PJ, Widome R, Gordon-Larsen P, Kershaw KN. Associations of smoke-free policies in restaurants, bars, and workplaces with blood pressure changes in the CARDIA study. *J Am Heart Assoc.* 2018; **7**(23): e009829.
104. McAlister AL, Huang P, Ramirez AG, Harrist RB, Fonseca VP. Reductions in cigarette smoking and acute myocardial infarction mortality in Jefferson County, Texas. *Am J Public Health*. 2010; **100**(12): 2391-2392.
105. McGhee SM, Wong CM, Schooling CM, et al. Smoke-free policies on population health outcomes. *Hong Kong Med J*. 2014; **20**(3 Suppl 3): 36-41.
106. McKinnon B, Auger N, Kaufman JS. The impact of smoke-free legislation on educational differences in birth outcomes. *J Epidemiol Community Health.* 2015; **69**(10): 937-43.
107. Menzies D, Nair A, Williamson PA, et al. Respiratory symptoms, pulmonary function, and markers of inflammation among bar workers before and after a legislative ban on smoking in public places. *JAMA*. 2006; **296**(14): 1742-1748.
108. Millett C, Lee JT, Laverty AA, Glantz SA, Majeed A. Hospital admissions for childhood asthma after smoke-free legislation in England. *Pediatrics.* 2013; **131**(2): e495-501.
109. Moraros J, Bird Y, Chen S, et al. The impact of the 2002 Delaware smoking ordinance on heart attack and asthma. *Int J Environ Res Public Health.* 2010; **7**(12): 4169-4178.
110. Naiman A, Glazier RH, Moineddin R. Association of anti-smoking legislation with rates of hospital admission for cardiovascular and respiratory conditions. *CMAJ.* 2010; **182**(8): 761-767.
111. Ozierański K, Witkowska A, Wojtyniak B, et al. Smoking ban in public places and myocardial infarction hospitalizations in a European country with high cardiovascular risk: insights from the Polish nationwide AMI-PL database. *Pol Arch Intern Med*. 2019; **129**(6): 386-391.
112. Page RL, 2nd, Slejko JF, Libby AM. A citywide smoking ban reduced maternal smoking and risk for preterm births: a Colorado natural experiment. *J Womens Health (Larchmt)*. 2012; **21**(6): 621-627.
113. Patanavanich R, Glantz SA. Association between tobacco control policies and hospital admissions for acute myocardial infarction in Thailand, 2006-2017: A time series analysis. *PLoS One.* 2020; **15**(12): e0242570.
114. Patrick SW, Warner KE, Pordes E, Davis MM. Cigarette Tax Increase and Infant Mortality. *Pediatrics.* 2016; **137**(1):e20152901.
115. Peelen MJ, Sheikh A, Kok M, et al. Tobacco control policies and perinatal health: a national quasi-experimental study. *Sci Rep*. 2016; **6**: 23907.
116. Pell JP, Haw S, Cobbe S, et al. Smoke-free legislation and hospitalizations for acute coronary syndrome. *N Engl J Med.* 2008; **359**(5): 482-491.
117. Polus S, Burns J, Hoffmann S, et al. Interrupted time series study found mixed effects of the impact of the Bavarian smoke-free legislation on pregnancy outcomes. *Sci Rep*. 2021; **11**(1): 4209.
118. Rajkumar S, Schmidt-Trucksäss A, Wellenius GA, et al. The effect of workplace smoking bans on heart rate variability and pulse wave velocity of non-smoking hospitality workers. *Int J Public Health.* 2014; **59**(4): 577-585.
119. Rodu B, Peiper N, Cole P. Acute myocardial infarction mortality before and after state-wide smoking bans. *J Community Health*. 2012; **37**(2): 468-472.
120. Sargent RP, Shepard RM, Glantz SA. Reduced incidence of admissions for myocardial infarction associated with public smoking ban: before and after study. *BMJ.* 2004; **328**(7446): 977-980.
121. Sargent JD, Demidenko E, Malenka DJ, Li Z, Gohlke H, Hanewinkel R. Smoking restrictions and hospitalization for acute coronary events in Germany. *Clin Res Cardiol.* 2012; **101**(3): 227-235.

© 2023 Akter S et al. *JAMA Network Open.*

122. Schoj V, Alderete M, Ruiz E, Hasdeu S, Linetzky B, Ferrante D. The impact of a 100% smoke-free law on the health of hospitality workers from the city of Neuquén, Argentina. *Tob Control*. 2010; **19**(2): 134-137.
123. Sebrié EM, Sandoya E, Hyland A, Bianco E, Glantz SA, Cummings KM. Hospital admissions for acute myocardial infarction before and after implementation of a comprehensive smoke-free policy in Uruguay. *Tob Control*. 2013; **22**(e1): e16-e20.
124. Séguret F, Ferreira C, Cambou JP, Carrière I, Thomas D. Changes in hospitalization rates for acute coronary syndrome after a two-phase comprehensive smoking ban. *Eur J Prev Cardiol*. 2014; **21**(12): 1575-1582.
125. Sen A, Piérard E. Estimating the effects of cigarette taxes on birth outcomes. *Can Public Policy*. 2011; **37**(2): 257-276.
126. Seo DC, Torabi MR. Reduced admissions for acute myocardial infarction associated with a public smoking ban: matched controlled study. *J Drug Educ*. 2007; **37**(3): 217-226.
127. Shelley D, Yerneni R, Hung D, Das D, Fahs M. The relative effect of household and workplace smoking restriction on health status among Chinese Americans living in New York City. *J Urban Health*. 2007; **84**(3): 360-371.
128. Shetty KD, DeLeire T, White C, Bhattacharya J. Changes in U.S. hospitalization and mortality rates following smoking bans. *J Policy Anal Manage.* 2010; **30**(1): 6-28.
129. Simón L, Pastor-Barriuso R, Boldo E, et al. Smoke-Free Legislation in Spain and Prematurity. *Pediatrics.* 2017; **139**(6).
130. Sims M, Maxwell R, Bauld L, Gilmore A. Short term impact of smoke-free legislation in England: retrospective analysis of hospital admissions for myocardial infarction. *BMJ.* 2010; **340**: c2161.
131. Stallings-Smith S, Zeka A, Goodman P, Kabir Z, Clancy L. Reductions in cardiovascular, cerebrovascular, and respiratory mortality following the national irish smoking ban: interrupted time-series analysis. *PLoS One.* 2013; **8**(4): e62063.
132. Stallings-Smith S, Goodman P, Kabir Z, Clancy L, Zeka A. Socioeconomic differentials in the immediate mortality effects of the national Irish smoking ban. *PLoS One.* 2014; **9**(6): e98617.
133. Thach TQ, McGhee SM, So JC, et al. The smoke-free legislation in Hong Kong: its impact on mortality. *Tob Control*. 2016; **25**(6): 685-691.
134. Trachsel LD, Kuhn MU, Reinhart WH, Schulzki T, Bonetti PO. Reduced incidence of acute myocardial infarction in the first year after implementation of a public smoking ban in Graubuenden, Switzerland. *Swiss Med Wkly*. 2010; **140**(9-10): 133-8.
135. Vander Weg MW, Rosenthal GE, Vaughan Sarrazin M. Smoking bans linked to lower hospitalizations for heart attacks and lung disease among medicare beneficiaries. *Health Aff (Millwood)*. 2012; **31**(12): 2699-2707.
136. Vicedo-Cabrera AM, Schindler C, Radovanovic D, et al. Benefits of smoking bans on preterm and early-term births: a natural experimental design in Switzerland. *Tob Control.* 2016; **25**(e2): e135-e41.
137. Vicedo-Cabrera AM, Röösli M, Radovanovic D, et al. Cardiorespiratory hospitalisation and mortality reductions after smoking bans in Switzerland. *Swiss Med Wkly*. 2016; **146**: w14381.
138. Villalbí JR, Sánchez E, Benet J, et al. The extension of smoke-free areas and acute myocardial infarction mortality: before and after study. *BMJ Open.* 2011; **1**(1): e000067.
139. Weaver AM, Wang Y, Rupp K, Watson DP. Effects of smoke-free air law on acute myocardial infarction hospitalization in Indianapolis and Marion County, Indiana. *BMC Public Health.* 2018; **18**(1): 232.
140. Wilson T, Shamo F, Boynton K, Kiley J. The impact of Michigan's Dr Ron Davis smoke-free air law on levels of cotinine, tobacco-specific lung carcinogen and severity of self-reported respiratory symptoms among non-smoking bar employees. *Tob Control.* 2012; **21**(6): 593-595.

141. Wu Y, Wang Z, Zheng Y, et al. The impact of comprehensive tobacco control policies on cardiovascular diseases in Beijing, China. *Addiction.* 2021; **116**(8): 2175-2184.
142. Xiao H, Zhang H, Wang D, et al. Impact of smoke-free legislation on acute myocardial infarction and stroke mortality: Tianjin, China, 2007-2015. *Tob Control*. 2020; **29**(1): 61-67.
143. Xiao H, Qi F, Jia X, et al. Impact of Qingdao's smoke-free legislation on hospitalizations and mortality from acute myocardial infarction and stroke: an interrupted time-series analysis. *Addiction (Abingdon, England)* 2020; **115**(8): 1561-1570.
144. Yan J. The Effects of a Minimum Cigarette Purchase Age of 21 on Prenatal Smoking and Infant Health. *East Econ J*. 2014; **40**(3): 289-308.
145. Yang YN, Huang YT, Yang CY. Effects of a national smoking ban on hospital admissions for cardiovascular diseases: a time-series analysis in Taiwan. *J Toxicol Environ Health A*. 2017; **80**(10-12): 562-568.
146. Yıldız F, Barış SA, Başyiğit I, Boyacı H, Aydınlık H, Sönmez P. Role of smoke-free legislation on emergency department admissions for smoking-related diseases in Kocaeli, Turkey. *East Mediterr Health J*. 2015; **20**(12): 774-780.
147. WHO. WHO report on the global tobacco epidemic, 2017. *Monitoring tobacco use and prevention policies* Geneva:World Health Organization, 2017.

© 2023 Akter S et al. *JAMA Network Open.*
