# Supplementary material for: Evaluation of Population-Level Tobacco Control Interventions and Health Outcomes: A Systematic Review and Meta-Analysis
Source: JAMA Netw Open. 2023 Jul 7;6(7):e2322341. doi: 10.1001/jamanetworkopen.2023.22341 (PMC10329215; doi:10.1001/jamanetworkopen.2023.22341)
Supplement: Supplement 2. — Data Sharing Statement [file jamanetwopen-e2322341-s002.pdf]

Data Sharing Statement

Akter. Evaluation of Population-Level Tobacco Control Interventions and Health Outcomes. *JAMA Netw Open*. Published July 07, 2023. doi:10.1001/jamanetworkopen.2023.22341

Data
Data available: No

Additional Information
Explanation for why data not available: The present study is a systematic review and meta-analysis, and we have no additional data to share.
